# A Water Compartment Cell Culture Lid Enables Stable Longitudinal Recording of Neuronal Networks In Vitro

**DOI:** 10.1002/advs.78463

**Published:** 2026-11-03

**Authors:** Benedikt Maurer, Fabio Fischer, Giulia Amos, Vaiva Vasiliauskaitė, János Vörös

**Affiliations:** ^1^ Laboratory of Biosensors and Bioelectronics Institute for Biomedical Engineering University and ETH Zurich Zurich Switzerland

**Keywords:** development, *in vitro* neuroscience, microelectrode arrays, neuronal networks

## Abstract

Longitudinal electrophysiological recordings of neuronal networks are essential for studying network maturation, plasticity, and pharmacological responses. Yet, current microelectrode array (MEA) approaches are limited by evaporation‐induced drift in culture conditions, exacerbated by heat dissipation from active recording electronics on CMOS‐based high‐density MEAs. We present a cell culture lid featuring a water compartment at its interface that eliminates evaporation whilst maintaining gas exchange. Combined with a custom incubator that uses independent temperature control of the MEA to prevent condensation, the system enables stable, uninterrupted recordings for weeks. We show that perturbations in firing rate and functional connectivity following medium exchange are significantly reduced by suppressing evaporation. We demonstrate continuous 35‐day recordings of patterned human iPSC‐derived neuronal networks with a single half‐medium exchange, revealing multiple spatiotemporal firing patterns that are held concurrently and evolve over weeks. All design files are provided, enabling adoption across culturing platforms for longitudinal studies of development within individual cultures, and establishing the conditions required for studies of plasticity and chronic pharmacology.

## Graphical abstract

A cell culture lid with an integrated water compartment eliminates evaporation from microelectrode arrays while preserving gas exchange. Freed from the osmotic drift and repeated medium exchanges that disturb conventional recordings, patterned human iPSC‐derived neuronal networks are recorded continuously for five weeks. Single‐spike resolution reveals independent firing patterns that emerge and evolve as the cultures mature.

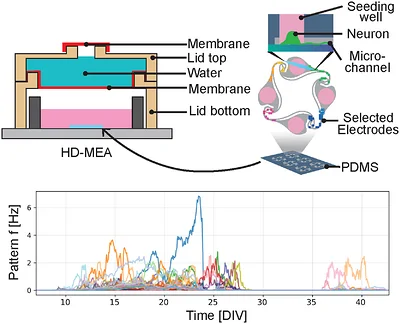

## 1 Introduction

Microelectrode array (MEA) recordings of neuronal cultures enable non‐invasive, long‐term electrophysiological readout, making them indispensable for drug screening, disease modeling, and fundamental neuroscience [1, 2, 3, 4]. Longitudinal MEA studies typically rely on periodic recording snapshots interleaved with regular partial medium exchanges to maintain culture conditions [5, 6]. However, each exchange transiently perturbs network activity, retains a fraction of altered medium, and poses contamination risks [7, 8]. At the low cell densities typical of MEA cultures, the dominant driver of medium deterioration is not metabolic waste accumulation but evaporation‐induced changes in ionic concentration [9, 10, 11], which is amplified in the case of partial medium exchanges (see Figure S14). As neuronal cells are highly sensitive to osmolarity shifts [12, 13], particularly in specialized neuronal media with reduced ionic buffering capacity [14], even modest evaporation compromises recording stability and culture health. Eliminating evaporation would allow the interval between medium exchanges to exceed the timescales of long‐term plasticity and network maturation — the very processes longitudinal MEA recordings aim to capture.

Existing mitigation strategies face a fundamental trade‐off between suppressing water vapor loss and maintaining CO_2_ and O_2_ exchange required for pH buffering and cell viability [9, 15]. Diffusion barriers such as sheet membranes [10, 11, 16] or silicone oil overlays [17] reduce but cannot eliminate evaporation, and the latter raises concerns regarding cytotoxicity and media integrity [18, 19]. Active humidity control systems can achieve higher suppression, but remain expensive, require dedicated equipment per recording station, and risk condensation damage to sensitive electronics [16, 20]. All diffusion‐limiting approaches only attenuate evaporation and therefore still require standard half‐medium exchange protocols, which lead to elevated, unphysiological ion concentrations as the affected medium remains partially inside the well.

Directly counteracting evaporation by adding purified water requires precise volume feedback control and introduces contamination risk [10, 13]. Continuous perfusion systems eliminate ionic drift through constant medium exchange [21, 22, 23, 24], but at the cost of complex microfluidic infrastructure, high medium consumption, and shear stress on cells — effectively substituting one source of perturbation for another.

Here, we present a fundamentally different approach: a culture lid with an integrated water compartment positioned above the liquid–air interface, which fully eliminates evaporation at its source by removing the humidity gradient, while preserving gas exchange. Combined with a temperature control system, this design maintains stable culture conditions with a strongly reduced number of medium exchanges and without active humidity regulation or perfusion infrastructure, and is readily scalable to parallel multi‐chip recordings with minimal modification to standard workflows.

We show that cultures under the water compartment lid exhibit significantly reduced transient perturbations in firing rate and functional connectivity following medium exchange compared to conventional membrane lids. By suppressing evaporation and its downstream confounds, we achieve what is, to our knowledge, the longest continuous, unperturbed recording of patterned neuronal networks on a high‐density MEA — over 35 days with only a single half‐medium exchange. The resulting dataset captures network development at unprecedented spatiotemporal resolution, revealing multiple firing patterns that are held concurrently and evolve over five weeks. These dynamics are invisible to conventional periodic recordings. All design files are provided to facilitate adoption across culturing platforms. Our platform enables uninterrupted longitudinal interfacing with network dynamics, allowing developmental trajectories to be followed continuously within a single culture and establishing the conditions, namely repeated intervention without an activity perturbation, that studies of plasticity and chronic pharmacology in individual cultures require.

## 2 Results

### 2.1 Water Compartment and Temperature Control Eliminate Evaporation While Permitting Gas Exchange

As described by Fick's law of diffusion, evaporation is driven by the gradient in water vapor concentration (or partial pressure). In a cell culture system with an exposed air‐liquid interface, the relative humidity of the air above the medium thus determines the evaporation rate, with dynamics governed by the diffusion barrier. Temperature gradients can further cause condensation on colder surrounding surfaces. Complementary metal–oxide–semiconductor (CMOS) based HD‐MEA systems are particularly susceptible to these effects, as active readout electronics on the chip dissipate power and heat up the culture medium. These mechanisms are summarized schematically in Figure 1a.

**FIGURE 1:**
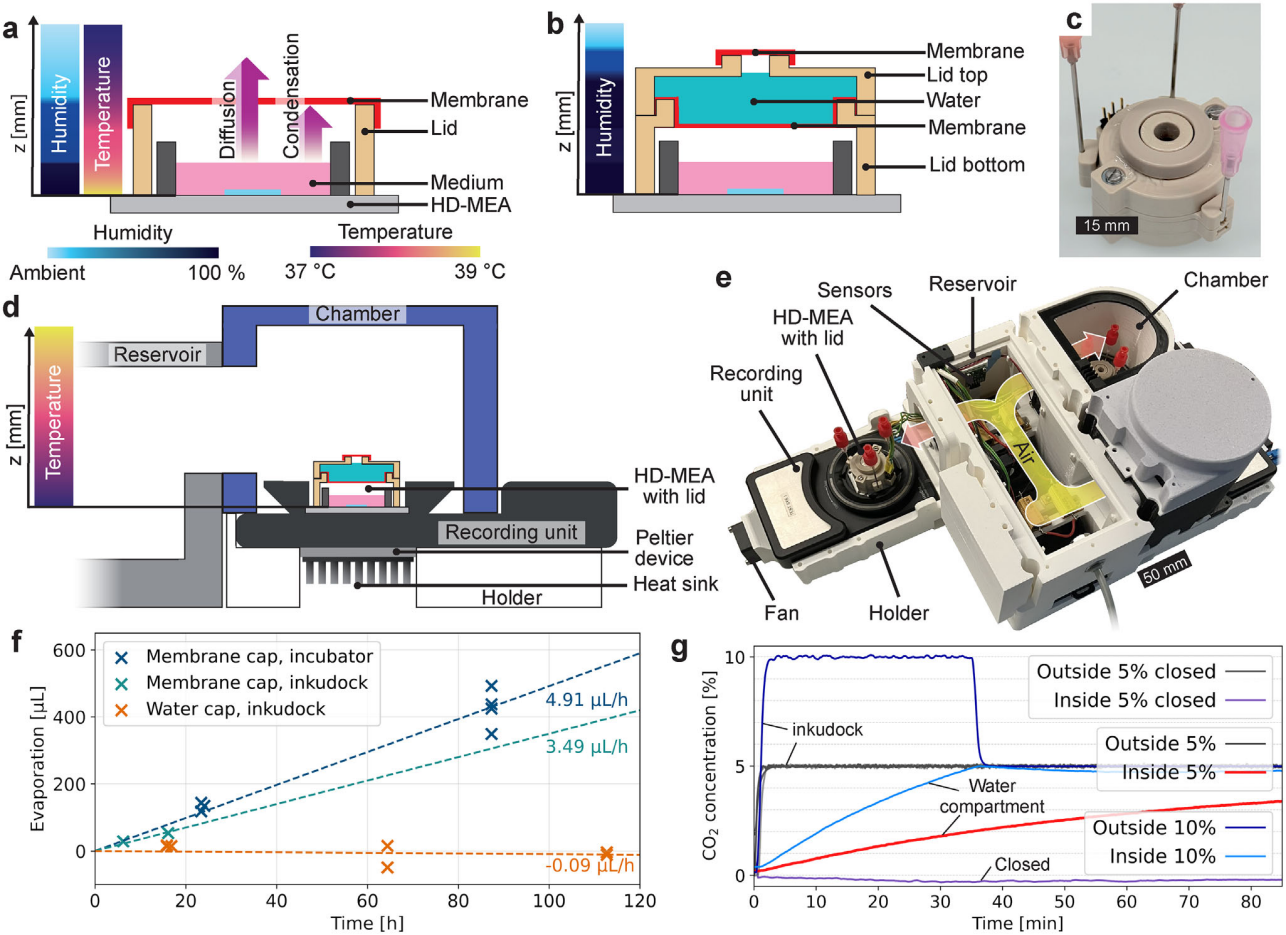
Addressing humidity and temperature gradients eliminates evaporation whilst preserving gas exchange.(a) Cell culture medium constantly evaporates from dishes due to humidity gradients. In the presence of recording systems, ambient humidity is further limited to prevent damaging electrical components. Additionally, temperature gradients, caused by incubators or power dissipation of active HD‐MEAs, lead to an elevated chip surface temperature with respect to its culturing environment. This raises evaporation levels and leads to condensation when common plastic or gas‐permeable membrane lids are employed, which enclose the chip to reduce evaporation and contamination risk. (b) Cross‐section of a culture lid with 2 gas‐permeable membranes (red) that enclose a water compartment (cyan), eliminating humidity gradients. (c) Depiction of the manufactured lid with a top part fixed with screws. The lower air compartment and the water compartment are hermetically sealed with three O‐rings. (d) A custom incubator setup inkudock, that controls the chip temperature to prevent condensation. Cross‐section of the design. The central reservoir resembles a standard incubator. A chamber is attached and encloses the chip with the lid. The recording unit is exposed to the ambient environment, therefore enabling easy temperature control by heating and cooling with a thermoelectric Peltier device. (e) A photo showing inkudock. Up to four chambers can be attached to one reservoir, distributing the airflow to all attached chips. (f) Evaporation from HD‐MEAs during recording is quantified in different setups by weighing the chips before and after. A linear fit is applied to determine the evaporation rate. When the temperature gradient is reversed by placing the chip with a single membrane lid (membrane cap) in inkudock, evaporation is reduced by about a third. Applying the water compartment lid (water cap) effectively eliminates the evaporation. (g) A sensor dummy that matches the dimensions of an HD‐MEA but carries a sensor board for humidity, ambient temperature, and ${\mathrm{CO}}_{2}$ concentration (see Section A.3.1) is used to measure the gas exchange. A lid where the lower membrane of the water compartment is replaced with solid material shows no increase in ${\mathrm{CO}}_{2}$ after a step to 5% in the inkudock, proving airtightness of the design. In contrast, the water cap shows ${\mathrm{CO}}_{2}$ transmission with a τ of about 75 min, averaged over 3 measurements. The time required for saturation at 5% can be shortened by stepping the ambient ${\mathrm{CO}}_{2}$ concentration to 10% for 35 min.

The proposed system introduces a water compartment positioned above a vapor‐permeable membrane, creating an interface with 100% humidity on the upper side. This compartment thereby serves as the buffer interface to the low‐humidity surroundings, isolating the culture medium below. To prevent depletion of the water compartment itself, it is sealed with a second membrane. The water layer creates a hermetically closed system. The working principle is illustrated in cross‐section in Figure 1b, with a manufactured prototype shown in Figure 1c.

To eliminate condensation, the temperature gradient must be balanced or reversed such that the medium and chip surface are cooler than the lid surfaces. In a conventional recording setup this condition is not met, since the culture is heated from below by the power dissipated in the CMOS chip and the recording unit (Figures S15 and S16). We present a custom incubator (‘inkudock’, Figure 1d,e), where a central reservoir serves as a standard incubator and individual chambers are attached that enclose an HD‐MEA mounted in the commercial recording unit (MaxOne+ and MaxOne Recording Unit, Maxwell Biosystems AG, Zurich, Switzerland). This seals the inside of the incubator from ambient conditions whilst keeping the bulk of the recording unit exposed to the outside, which enables independent temperature control. As the chip is strongly thermally coupled to the recording unit, the lid can be heated via the reservoir temperature without strongly affecting the chip temperature. Alternatively, the water compartment can be actively heated to invert the temperature gradient when placed in a standard commercial incubator (see Section A.1.3).

To validate the system's performance, we first quantified evaporation by weighing HD‐MEAs before and after continuous recording under different configurations (Figure 1f). To achieve physiological conditions for the cells in inkudock, the HD‐MEA surface temperature was set to 38.5 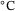, resulting in a medium temperature of 35.5 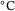 at a chamber temperature of about 43 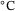 (for calibration procedure see Section B.3). The temperature in a commercial incubator with a passive water bath (CB170, BINDER GmbH, Tuttlingen, Germany) was set to 35 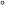 C. Placing a chip with a common single‐membrane lid (see Methods) in inkudock reduced evaporation by approximately 30% compared to the incubator. Evaporation rate without recording in the incubator ranges between 0.8 (see Figure S18) and 2.5 μL/h [16]. The water compartment lid effectively eliminated evaporation, with one negative weight change attributed to condensation on the culture medium due to suboptimal temperature sensor placement. Evaporation from the water compartment itself was tracked by weighing the lid over the same experiments (Figure S19). A linear fit gives 1.11 μL/h, in the same range as the 0.8–1.0 μL/h measured for MEA wells under a membrane lid in commercial incubators (Figure S18), as expected given the comparable dimensions and the absence of thermal coupling to the recording unit. Since the vapor barrier relies on a continuous water layer between the two membranes, the compartment is refilled before gaps can form in that layer rather than when it approaches depletion, which in all experiments reported here meant a refill at least every 10 days.

Gas exchange through the water compartment was tested using a validation chip with dimensions matching the employed HD‐MEAs, but equipped with a sensor board for humidity, temperature, and ${\mathrm{CO}}_{2}$ concentration (see Figure S12). Figure 1g shows the ${\mathrm{CO}}_{2}$ concentration step response. A control measurement with the lower membrane replaced by solid material confirmed hermetic sealing of the remaining design. With the water compartment, ${\mathrm{CO}}_{2}$ transmission exhibited a time constant τ of approximately 75 min with no liquid in the well. The equilibration time is shortened with lower filling volume, which can be seen in the additional data in Figure S20. To accelerate saturation, we applied an initial step to 10% ${\mathrm{CO}}_{2}$ for 35 min, before returning to 5%. Additional tests with ${\mathrm{CO}}_{2}$‐saturated water indicated that transmission from the water compartment to the lower air gap dominates the saturation dynamics. At the cell densities typical of MEA cultures, these ${\mathrm{CO}}_{2}$ equilibration dynamics are expected to maintain pH buffering.

### 2.2 Transient Changes in Network Activity Caused by Medium Exchange are Suppressed Through Evaporation‐Suppressing Lid

Patterning neuronal networks in microfluidic devices prevents cell migration and thus forms an ideal platform for longitudinal recordings [26, 27]. We used patterned networks to assess the effects of reduced evaporation on electrophysiology and network dynamics. PDMS membranes confine cell bodies in seeding wells, which are connected through microchannels. We applied them to HD‐MEAs and selected the microchannel regions for continuous recording of action potentials (overview in Figure 2a, for details see Methods). The impact of medium exchange on the firing rate has been documented in literature [6, 28, 29] and is accounted for in MEA recording protocols [30]. To investigate whether evaporation‐induced osmolarity shifts contribute to these changes, we compared cultures equipped with a membrane‐sealed lid versus our water compartment lid, assuming previously determined evaporation rates. We continuously recorded the cultures in inkudock and performed a medium exchange every 3 to 4 days. The two groups did not differ significantly in baseline firing rate or in the balance of activity across the four regions (Figures S21 and S22). Networks cultured with a conventional membrane lid exhibited significantly greater transient changes in the relative firing rate immediately following medium exchange when performing a linear fit (Figure 2b–d). We additionally assessed the recovery after medium exchange. Under the membrane lid, the firing rate rarely returned to baseline within six hours, whereas under the water compartment lid it stayed close to baseline throughout (Figure S23), suggesting that the exchange triggers a lasting reorganization of network activity.

**FIGURE 2:**
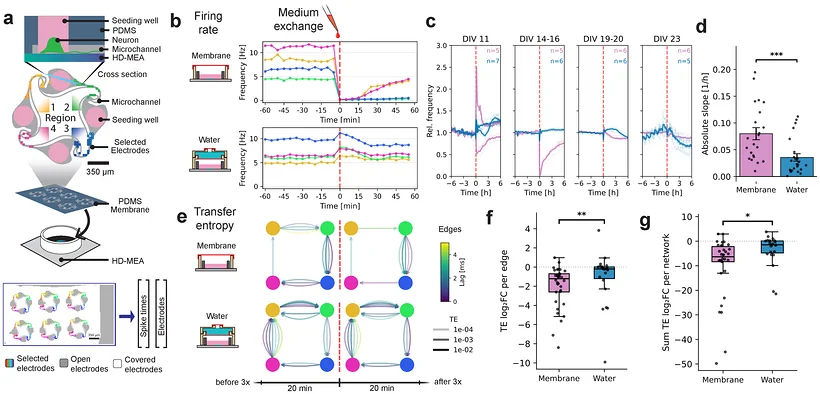
Comparison of the impact of medium exchange on network activity.(a) Compartmentalized networks were formed on top of an HD‐MEA with a two‐layer PDMS membrane, which restricts cell bodies from migrating from the seeding well into the microchannels [25]. Electrodes were selected inside the microchannels with an impedance scan and summarized into 4 regions. Spikes from up to 6 networks in parallel were simultaneously recorded using the MaxLab Live application programming interface (API). (b) A chip with a single‐membrane lid and one with the water lid were placed inside inkudock and recorded continuously. Every 3–4 days, half of the culture medium was exchanged with fresh medium. The firing rates of the 4 regions for one example network per condition 1 h before and after the exchange are shown, averaged across 5 min. (c) The firing rate of several networks on different MEAs, divided by the mean firing rate in the 6 h before an exchange are shown for different DIV. The average across the networks on the same MEA is shown as thick lines. (d) The slope of a linear fit on the relative rate change in the first 4 h after exchange was compared across the conditions. Mann–Whitney U testing shows significantly less impact of medium exchange on the activity with the water compartment lid (20 unique networks; p<0.001; linear mixed model with network identity as random intercept confirmed negligible between‐network variance across DIVs). (e) Transfer entropy between network regions was calculated as described in Methods. Example networks 20 min before and after the exchange are shown for both conditions. Only networks with a weakly connected component of at least 3 nodes were used for further analysis. The total analysis comprised 25 unique networks with 81 network‐exchange observations (54 membrane, 27 water). (f) Persistence of edges across medium exchange was assessed. Stable edges were defined as connections with TE $\geq 1\times 10^{-4}$ at the same lag across all three 20 min windows before exchange. The mean log_2_ fold change of transfer entropy per stable edge is shown (disappearing edges were floored to $\mathrm{TE}\;=\;1\times 10^{-6}$). Networks with the water compartment lid showed significantly lower changes (Mann–Whitney U; p<0.01). The number of stable edges was 175 and 113 for the membrane and water compartment lid, respectively. (g) The fold change summed across a network also showed higher persistence with the water compartment lid (linear mixed model with network as random intercept; p<0.05).

Functional connectivity within a network is a common readout for assessing circuit‐level dynamics [31, 32]. We therefore tested whether medium exchange altered directed information flow beyond global changes in firing rate. Effective connectivity was quantified using transfer entropy within a 5 ms time window, computed from 20 min segments of spike data. Figure 2e shows the transfer entropy as directed edges with different lags. Stable directed edges were defined as connections with TE $\geq 1\times 10^{-4}$ (Figure S24) at a consistent lag across all three pre‐exchange windows (Figure S25). Edges were tracked across the exchange by computing the log_2_ fold change of their mean TE with the TE at the same lag after exchange (Figure 2f,g). The mean fold change per edge showed a roughly 2.5‐fold stronger reduction with the membrane lid (Mann–Whitney U, p<0.01). Also, when summed across a network, the membrane lid showed stronger reorganisation (linear mixed model with network as random intercept, p<0.05). The direction of both effects holds for edge thresholds between $1\times 10^{-5}$ and $1\times 10^{-3}$, and the per‐edge effect remains statistically significant across this range (Figure S26). Medium exchange thus induced a more pronounced reconfiguration of directed functional interactions for the membrane lid in the presence of substantial evaporation. This indicates a transient displacement of the network from its ongoing dynamical regime, affecting information‐theoretic readouts.

Notably, since evaporation with the membrane lid in inkudock is substantially lower than the rates expected during continuous recording in a commercial incubator, the observed effects are a conservative estimate of the medium exchange impact under typical recording conditions.

### 2.3 Persistent Network Activity can be Continuously Recorded for Weeks Without Intervention

With our system, recordings that were previously limited to a few hours due to evaporation‐induced changes in firing rates can now be performed for days. The reduced impact of medium exchange under the water compartment lid (Figure 2) was also observed in a mature culture recorded over twelve days in inkudock, in which two half‐medium exchanges produced no lasting deflection of the firing rate (Figure S27). After the same culture was transferred to a state‐of‐the‐art recording configuration, a standard incubator with a commercial membrane lid, its activity was perturbed within hours and ceased within approximately eight days. Considering the minimal impact of medium exchange on culture activity under the water compartment lid, we reduced the frequency of medium exchanges for the long‐term recordings. This enables the characterization of network maturation without the confounding effects of medium exchange. We performed continuous recordings of two HD‐MEAs equipped with water compartment lids for 35 days (see Figure 3 for electrophysiology, environmental parameters are shown in Figure S28). Recording was started at 7 DIV following a full medium exchange, and interrupted after 3 weeks by a single exchange, where half of the culture medium was replaced with fresh medium. Both chips exhibited remarkably similar developmental trajectories, with network activity emerging after a few days, as is expected from these cultures [33, 34]. Activity levels rose progressively before plateauing between 14 and 21 DIV, after which firing rates declined on both chips. Notably, the proportion of active units decreased less dramatically than the overall firing rate, indicating that many electrodes continued to record sparse activity even as network‐wide firing diminished. The spike amplitude distribution remained stable over most of the recording (Figures S29 and S30) and shifted to lower values only during the period of low activity, when high‐amplitude units ceased firing (Figure S31). Network bursting increased progressively up to this period (Figures S32 and S33). This decline could be attributed to nutrient depletion, as the exchange of culture medium initiated a slow recovery. Glucose measurements showed a decrease by 20% in 14 days, while protein electrophoresis and UV–vis spectroscopy indicated stable levels of protein content (see Section D.5). As both chips followed similar trajectories, a cause affecting both cultures alike, such as a change in medium composition, is more likely than a failure of an individual lid.

**FIGURE 3:**
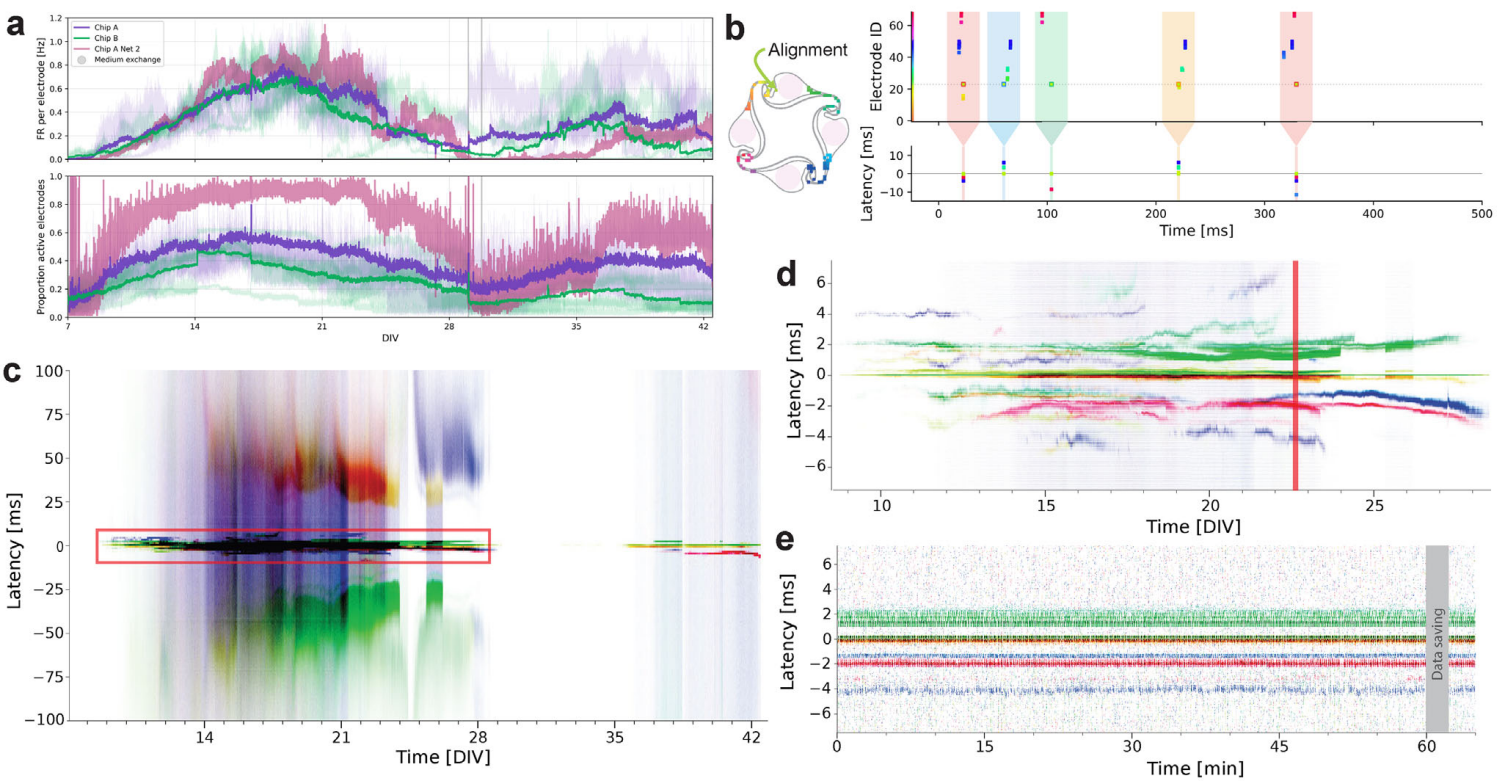
Network activity of two HD‐MEAs with a water compartment lid is continuously recorded for 35 days. (a) Firing rate per electrode and proportion of active electrodes over time, averaged across 1 min. Networks are considered active if their average firing rate lies above 0.1 Hz. The HD‐MEAs contain 6 independent networks each (faint lines, with network 2 from chip A highlighted in pink). The recording was started at 7 DIV, with a full medium exchange prior to the recording and an exchange of half the medium after 3 weeks. The water compartments were refilled after 10 days. Spikes were recorded for 1 h followed by a break of 2 min for data saving. The firing rate decreased after 21 DIV on both chips, but recovered after the medium exchange. (b) Spike‐time‐triggered raster plots (STTRPs) are generated to visualise network activity over days. The most active electrode in a network is used for alignment, and spikes in its vicinity (colored windows in the upper axis, here for ± 15 ms) are plotted with their respective latencies at the spike‐time of the alignment spike (lower axis). All spikes are plotted; no clustering, averaging or selection of recording periods is applied. (c) Full STTRP of network 2 from chip A with a maximum latency of ± 100 ms, binned to 30 min. Spikes are color‐coded following panel b, with a firing frequency of 2 Hz leading to saturation of a pixel with the electrode color. After 14 DIV, synaptic activity formed bands of consistent latencies at ± 50 ms. Over a time course spanning about 2 weeks latency decreased until the firing at this relatively stable latency abruptly disappeared. (d) Zoom‐in on 8.5–28.5 DIV and a latency of ± 10 ms (red rectangle in panel c). Sequential spikes across the whole network are visible, presumably relying on fast synaptic transmission between the network nodes. Several bands of activity with consistent latency and low probability occur, especially between 14 and 24 DIV. (e) Zoom‐in to 1 h on 22.5 DIV (red bar in panel d). The activity is remarkably stable during the conventionally applied recording duration of tens of minutes.

We performed a detailed analysis of the spatiotemporal network dynamics over time on network 2 from chip A (firing rate shown in Figure 3a), where we acquired about 800 million spikes across 35 days. For visualization, we aligned the spontaneous network activity to its most active electrode and investigated the relative latencies of spikes on the remaining network electrodes over time with a spike‐time‐triggered raster plot (STTRP, see Figure 3b). When spikes reliably occur at a similar latency over time, a horizontal band is visible in the STTRP, suggesting a strong connection between the neurons recorded on those electrodes. At long latencies, synaptic activity bands appear around ± 50 ms, indicative of polysynaptic transmission from the alignment to the observation electrode [35, 36, 37]. Zooming in to shorter timescales (± 10 ms) reveals circular propagation patterns consistent with the directional recurrent network architecture. As activity begins to decline, firing latencies increase, suggesting slowed conduction or weakened synaptic transmission. An additional network is presented in the Section D.6 with Figure S38.

### 2.4 Independent Spontaneous Firing Patterns Develop Simultaneously in Maturing Cultures

Persistent sequences of spontaneous activity are a frequently studied target, due to their assumed importance for memory formation and retention [38, 39]. In our platform, the axons of all cells are guided through the microchannels, where the HD‐MEA records their activity at single‐cell resolution, and the recording is continuous, so that recurring sequences can be identified and followed over time. Analyzing their trajectories through continuous recording represents one of the many potential benefits of the system presented here. To quantify how spontaneous activity is composed of recurring firing patterns and how this composition changes over time, we developed a clustering pipeline for low‐latency sequences of activity (Figure 4a–d). To reduce the electrode dimensionality, spikes recorded within a window of 1.5 ms on multiple electrodes belonging to the same region of the microstructure corresponding to the underlying microchannel geometry were summarized into a region event (for details see Methods; the merge window, the encoding kernel and the analysis window are checked against the data in Figures S39– S41). Region‐specific firing sequences were then aligned to the first region event (on the region with the lowest ID), windowed to ± 10 ms around this reference, and converted into matrices. A density‐based clustering (HDBSCAN, see Methods) was performed on the first 5 principal components and visualized via t‐SNE, showing clear cluster separation for most patterns (Figure 4e). Example sequences from the 4 most prominent clusters, which stand for a consistent activity pattern, demonstrate low intra‐cluster variability. All sequences arranged by pattern and sorted by the first principal component are provided in Figure S42. Cluster averages and pairwise similarities (Figures S43 and S44), internal validity indices (Figure S45, Table S3), and repeated clustering with other algorithms, numbers of principal components, density parameters and fitting subsamples (Figures S46– S48, Tables S4 and S5) confirm the pattern assignment.

**FIGURE 4:**
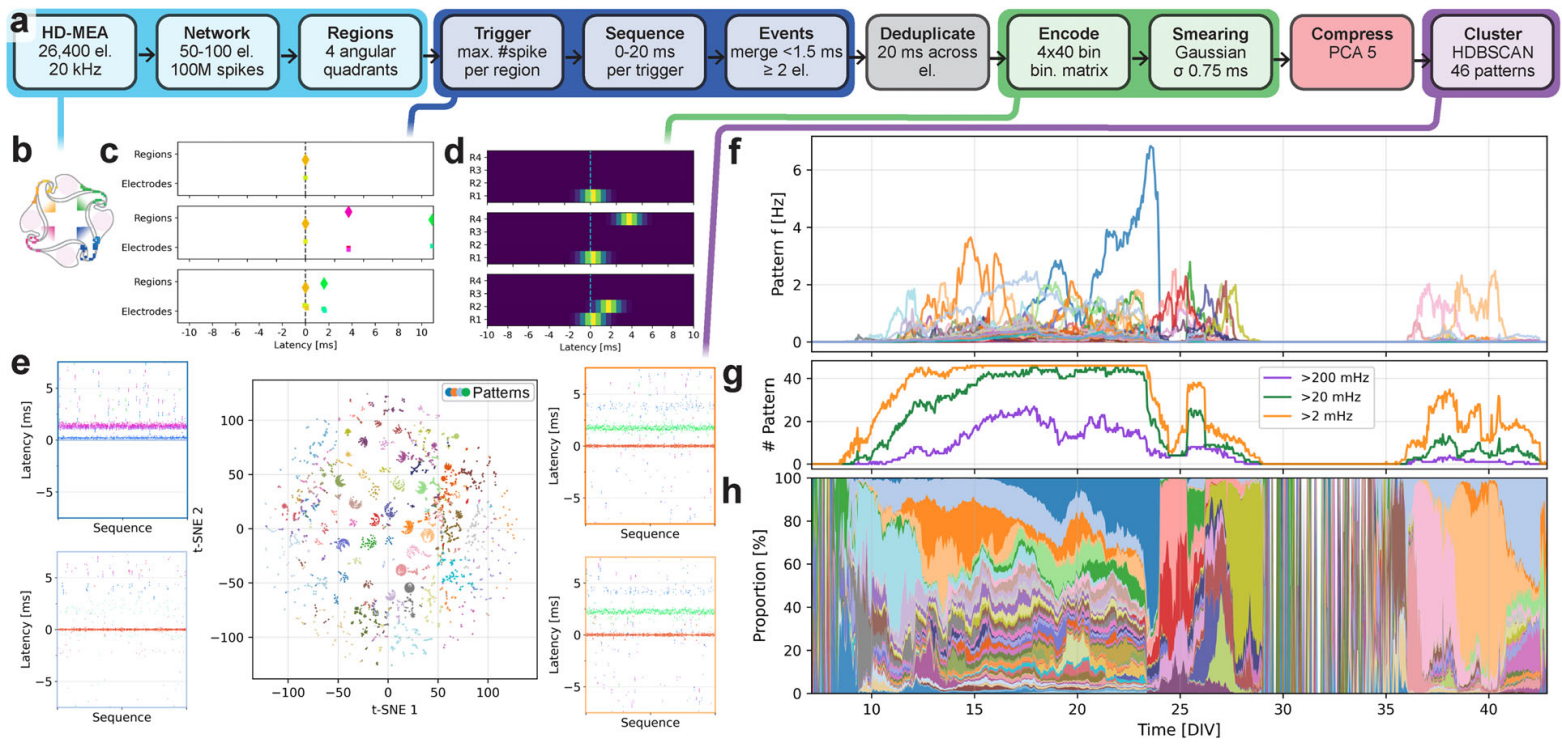
Simultaneously active, recurring firing patterns develop over weeks of activity. (a) Data processing pipeline. Network electrodes were selected on the HD‐MEA and assigned to microchannel regions by angular limits, and the most active electrode of each region served as the alignment trigger. Sequences were extracted as in Figure 3b, with individual spikes aggregated into region events (see Methods) and sequences detected on several alignment electrodes removed by deduplication. Each sequence was then converted into a binary matrix, convolved with a Gaussian kernel and reduced by PCA, before density‐based clustering grouped the sequences into clusters corresponding to recurring patterns. (b) Network electrodes were aggregated into 4 regions corresponding to the microchannels. A color code was applied, mapping the hue circle onto the angular rank of the electrodes with gaps for the wells. (c) The most active electrode was selected for each region and firing sequences, with at least one spike detected within ± 10 ms on another electrode, were extracted. The dimensionality of the firing sequences was reduced for clustering. When more than 2 spikes occur in the same region within 1.5 ms, they were registered as a region event (diamonds). A few examples are shown next to the original spike data by electrode, following the color code in (b). About 15 million sequences were identified. (d) Sequences were aligned to the first region event (in the region with the lowest ID) and transformed into spatiotemporal matrices spanning ± 10 ms with a Gaussian kernel as described in Methods. (e) PCA was performed on the flattened matrices. Clustering was performed using HDBSCAN on the first five principal components, yielding 46 recurring patterns. The 2D t‐distributed stochastic neighbour embedding (t‐SNE) shows the cluster distribution with 500 example sequences from the 4 most frequent patterns (for all sequences see Figure S42). (f) The frequency of the patterns was tracked over the whole experiment duration and aggregated into bins of 1 h. The color code is selected according to (e). Stable patterns emerged around 10 DIV. The most frequent pattern peaks at more than 6 Hz. (g) The number of patterns surpassing 3 different frequency thresholds is shown over time. When looking at the pattern diversity, the number of patterns occurring with a frequency above 20 mHz plateaus at about 15 DIV and drops rapidly from 23–24 DIV. (h) The distribution of patterns is shown by proportion. After 36 DIV, single patterns dominate the distribution.

Figure 4f shows when and how often specific patterns occurred during the experiment. The number of concurrently active patterns increased steadily until it plateaued at 17 DIV (Figure 4g). The first stable firing patterns emerged around 10 DIV, with multiple low‐frequency sequences coexisting as early as 12 DIV up to 24 DIV alongside an increasingly dominant cluster. This period overlaps with the onset of synapse formation reported for these neurons after 2 to 4 weeks in vitro [40, 41]. After 24 DIV, fewer patterns were simultaneously active (Figure 4h). This can be partially attributed to the concurrent decrease in firing rate, at which rarer patterns are observed less often. It could also indicate a manifestation of patterns through more stable synaptic connections [42, 43]. Following the period of near‐silence, activity re‐emerged at 36 DIV, with a few prominent patterns. Some of these patterns had already been active before the quiet interval. Throughout the recording, several patterns were held concurrently, with occurrence rates that changed independently of one another (Figure 4f).

The directed functional connectivity between the four regions, quantified by delayed transfer entropy as for Figure 2 and independently of the clustering, remained highly consistent over the recording (Figure S49). Over a few hours the network was stable, with a high similarity to the network 6 h earlier. Over longer timescales, most rows of the all‐to‐all similarity matrix appear multimodal rather than centered on the diagonal, indicating recurring periods of higher similarity rather than a uniform drift. Since the connectivity is derived at the level of regions without resolving individual units, it cannot distinguish whether concurrent patterns share connections or are carried by independent units within a region. The concurrency of several patterns is nevertheless notable in a network of only four compartments.

Burst analysis after Wagenaar et al. [44] was performed for comparison. The burstiness index remained below about 0.2 up to 23 DIV and rose to about 0.4 at 25 DIV, before the quiet interval (Figures S32 and S33). Such population‐level measures describe how much and how synchronously the network fires, whereas the sequence analysis resolves the propagation paths of which the activity is composed. During the quiet interval, the number of detected spikes fell by more than an order of magnitude. At the same time, the spike amplitude distribution shifted to lower values, indicating that a portion of the high‐amplitude units ceased firing (Figure S31).

The mechanisms underlying the quiet interval cannot be resolved with the present data. Homeostatic regulation of firing [45, 46], which could transiently overshoot after the preceding days of high and increasingly bursty activity, synaptic remodeling [47] and changes in culture conditions are all compatible with it.

The same analysis applied to a more mature culture on a different microstructure, recorded from 49 DIV, showed fewer changes in its pattern repertoire, but likewise several concurrently held patterns that persisted over days (Figures S50– S53). The additional network recorded for 14 days followed a trajectory similar to that of chip A, both in its pattern repertoire (Figures S54 and S55) and in its functional connectivity (Figure S49), further suggesting that the medium remains intact over at least 14 days without exchange.

Prior studies have described a rich repertoire of evolving burst patterns during maturation of dissociated cortical cultures, with pattern diversity highest while synaptic connections are still forming [44]. Periodic recordings have shown that synchronized activity emerges progressively and stabilizes once mature [48], and continuous perfusion‐based recordings confirmed that activity remains broadly stable in the absence of external perturbation [22]. These observations were largely limited to population‐level burst statistics, as passive low‐density MEAs sample only a fraction of active units in large, randomly connected networks. The patterned networks and HD‐MEA recordings employed here extend this view to single‐spike spatiotemporal sequences, enabling the tracking of individual firing patterns across weeks of uninterrupted development.

## 3 Discussion and Conclusions

With the advancements in cellular models and MEA technology, an easily usable, ready‐to‐scale solution eliminating evaporation‐induced changes in ionic concentration is required for higher‐quality assays and longitudinal studies.

We developed a hermetically sealed cell culture lid with a water compartment interface that eliminates evaporation of cell culture medium whilst maintaining gas permeability. Combined with integrated temperature sensing and a custom incubator that prevents condensation through independent temperature control, the system enables stable weeks‐long MEA recordings with two to three weeks between medium exchanges. We were able to track the maturation process and firing pattern diversity.

To improve usability, membrane protection against rupturing, overpressure and refill access ports, and an integrated, wireless temperature control of the water compartment for use in commercial incubators could be added. Furthermore, active humidification of inkudock, a smart refill system, or a humidified chamber above the second membrane, can prevent an empty water compartment. The presented lid design is easily adaptable to other culturing formats, such as multiwell plates.

Our platform addresses a fundamental limitation in long‐term electrophysiology by providing the environmental stability necessary for reliable effect attribution in studies of network maturation, plasticity, and chronic pharmacological interventions. By enabling continuous recordings with two to three weeks between medium exchanges, the system reduces media consumption and manual intervention whilst improving culture sterility through hermetic sealing and relaxing constraints on incubator humidity. Notably, we demonstrated compatibility with human iPSC‐derived neurons, which represent a highly relevant model system for studying neurodevelopmental processes and disease‐associated phenotypes yet are particularly sensitive to environmental perturbations [49]. Medium analysis, the recovery of the spike amplitude distribution after the quiet interval and the persistence of the active electrode fraction are all consistent with preserved culture integrity, but they are indirect, and a contribution of changing viability or synaptic composition to the activity changes reported here cannot be excluded. An in‐situ assessment of viability would complement the electrophysiological readout.

Continuous monitoring offers several advantages over periodic recording snapshots. It captures transient phenomena that would otherwise be missed, and reduces handling‐induced variability. A first study highlights the applicability of the setup in studies of transient disease progression [50]. It also facilitates interfacing cultures with computational models for closed‐loop experiments. Finally, fewer biological replicates are needed to achieve the same statistical power, as within‐culture variance is reduced. Together, these improvements enable more robust and efficient investigation of long‐term network dynamics.

The presented methodology suggests that, as a result of the limited complexity of connections, recurring sequences of spatiotemporal activity can be identified. The fact that multiple patterns are active in an unsynchronized manner already in such a small network indicates that seemingly chaotic population‐level activity might also be a superposition of simpler sequences of neurons firing with distinct latencies. The analysis requires that activity can be assigned to a limited number of spatially distinguishable sites with resolvable interaction delays, and that the propagation paths between them remain separable, which patterned networks on HD‐MEAs provide particularly well. Nothing prevents its extension to other configurations, including other patterned geometries, electrode layouts, organoids and unconstrained cultures, where the definition of such sites and the separability of the propagation paths will determine its applicability.

## 4 Methods

### 4.1 Hardware Description

#### 4.1.1 Water Compartment Lid

The lid comprises six CNC‐milled components made of polyether‐ether‐ketone (PEEK) assembled with epoxy adhesive and screws. Two membranes positioned above the chip well create a water‐filled compartment with an air gap. Three bent stainless‐steel needles enable liquid handling during operation. A laser‐cut copper plate electrically grounds the needles via screw contacts, preventing noise induction. A PT1000 temperature sensor integrated into the bottom component monitors chip surface temperature. Airtight sealing is achieved through epoxy bonding along the glue ridge and compression of o‐rings at the membrane and chip interfaces. Detailed fabrication instructions, including a reduced version without fluidic inlets, are provided in Section A.1; design files are available in the repository listed in the data availability statement.

#### 4.1.2 Custom Incubator

All experiments were performed in a custom incubator (inkudock) consisting of a central reservoir and up to four attachable chambers, all 3D‐printed from polylactic acid (PLA). The reservoir contains a sensor board [51], resistive heaters, a ${\mathrm{CO}}_{2}$ inlet, and circulation fans. Each chamber is mounted atop a MaxOne recording unit, which sits on a 3D‐printed holder equipped with a Peltier device and heat sink for temperature control. Electronics were adapted from the inkube system [13], with temperature and ${\mathrm{CO}}_{2}$ control for the reservoir and four independent temperature control loops for the recording units. Sensor signals are routed through a custom shield on the Arty Z7 development board (Digilent, Pullman, WA, USA) to a system‐on‐chip (XC7Z020‐1CLG400C, Xilinx, San Jose, CA, USA), which then transmits control signals through the shield to a power board for amplification. Modifications include an updated shield with PT1000 readout circuitry and a revised power board with more powerful single‐ended amplifiers for reservoir temperature and humidity control. Hardware design files, derived from the inkube system [13], are released under the Creative Commons Attribution‐ShareAlike 4.0 International licence (CC BY‐SA 4.0; https://creativecommons.org/licenses/by‐sa/4.0/), see data availability statement. Assembly instructions are provided in the Section A.2.

#### 4.1.3 Sensor Dummy Chip

A sensor dummy chip with dimensions matching the HD‐MEA was CNC‐manufactured from PMMA. The dummy incorporated an inkusense sensor board [51] equipped with humidity, temperature, and ${\mathrm{CO}}_{2}$ sensors, positioned on top of a dummy well that can be filled with liquid. Cables were routed through the bottom of the assembly and hermetically sealed with epoxy (see Supplementary Information A.3.1).

#### 4.1.4 Sensor Ring

A custom sensor ring mounted beneath the conventional single‐membrane lid contains a PT1000 temperature sensor pressed to the chip surface. The ring was fabricated using LCD resin 3D printing. Details are provided in the Section A.3.2.

### 4.2 Cell Culturing

#### 4.2.1 Chip Preparation

High‐density microelectrode arrays were prepared with polydimethylsiloxane (PDMS) microstructures as described previously [52]. Briefly, the chip surface was coated with poly‐D‐lysine (PDL), and a PDMS membrane was cut from a wafer and positioned onto the dried surface using tweezers. The MEA well was filled with phosphate‐buffered saline (PBS) and desiccated to remove air bubbles, then PBS was replaced with culture medium before cell seeding. The two‐layered PDMS microstructures, manufactured by Wunderlichips GmbH (Zurich, Switzerland) using soft lithography, consisted of open‐top nodes for cell bodies connected by enclosed microchannels. The microstructures employed 4‐node recurrent designs that promote directional axonal growth [25], with 6 full networks fitting onto the chip sensing area.

#### 4.2.2 Culture Preparation

Early‐stage Ngn2‐induced neurons derived from human iPSCs [33] were obtained from Novartis (Basel, Switzerland), thawed and differentiated using neurobasal differentiation medium (NBD+): Neurobasal Plus (A3582901) with GlutaMAX (35050‐061) and Pen‐Strep (15070‐063), supplemented with B‐27 Plus (A3582801), N2 (17502‐048), BDNF (10 μg/mL, 450‐10) and GDNF (10 μg/mL, 450‐02, all Thermo Fisher Scientific, Waltham, MA, USA), as previously described [34]. For suspension seeding (long‐term recordings), cells were plated at 15 0000 cells per chip in NBD supplemented with 5 μg/mL laminin (L2020, Sigma Aldrich, St. Louis, MO, USA) and 2 μg/mL doxycycline (631311, Takara Bio USA, Inc., Mountain View, CA, USA). Complete medium exchanges were performed at 60 min and 7 days post‐seeding. For spheroid seeding (comparison experiments), cells were aggregated at 100 cells per spheroid using AggreWell 400 plates (34415, StemCell Technologies, Vancouver, Canada) following the manufacturer's protocol. Spheroids were seeded after 1 day into medium supplemented with doxycycline and laminin, with full exchange at 7 days post‐plating. Doxycycline and laminin supplementation was maintained for the first 7 days after plating in both protocols.

#### 4.2.3 Membrane Lid

MEAs were kept under a standard single‐membrane lid outside of experiments and during controls. We used an open‐source lid, kindly provided by the University of California Santa Cruz, based on the original by Potter et al. [10], optimized for the MaxOne chip geometry with a tighter fit (see Figure S3 in [21]; design available on GitHub as membrane_ring.stl [53]). The outer holder ring was sealed with a fluorinated ethylene propylene membrane sheet of 12.5 μm thickness (ALA MEA‐MEM‐SHEET, ALA Scientific Instruments Inc.).

### 4.3 Electrophysiology

#### 4.3.1 Electrode Selection

Due to the 1024‐channel recording limit of the MaxOne HD‐MEA, electrodes were preselected based on PDMS microstructure placement. An impedance scan was performed as described in previous work [54]. A threshold was applied to the impedance map to identify PDMS‐covered regions and distinguish them from open electrode sites. Electrodes within the open regions in the microchannels connecting the wells were selected for analysis, with some selections expanded to include all electrodes from one entire network.

#### 4.3.2 Spike Data

Spike detection was performed using the MaxLab Live Python API (Maxwell Biosystems AG, Zurich, Switzerland) with detection thresholds of 4.5 standard deviations for exchange experiments and 5 standard deviations for long‐term recordings. Channels with a mean firing rate below 0.1 Hz were excluded from all analyses. Only spike times and amplitudes were stored, as storing the raw signal over several weeks was not feasible. A histogram of detected spike amplitudes is presented in Figure S29, showing the amplified signals in microchannels [55], with values within the range previously reported for such recordings [52]. Neural activity was recorded in continuous chunks of 1 (long‐term) or 2 h (exchange). Time delays between chunks interrupted by medium exchange were calculated from file generation timestamps, as the spike timing resets upon hardware restart.

### 4.4 Network Activity Analysis

### 4.5 Spike Data Dimensionality Reduction

For transfer entropy calculation and pattern clustering, unit‐level activity of the neuronal cells is more relevant and computationally efficient compared to electrode data. Spike sorting assigns electrode‐level data to individual cells [56]. As axonal growth is confined by the microstructure, electrodes located beneath the same microchannel record largely redundant activity from the same axon when an action potential is travelling along the cell [55]. In order to reduce the data dimensionality, the same action potential recorded by different electrodes should therefore be captured as a single spike event. Each electrode was assigned to one of four regions based on its angular position relative to the network centre, yielding four quadrants corresponding to the microchannels. Each of the regions comprises about 30 to 50 electrodes. Within each region, spikes occurring within a 1.5 ms window were merged into a single event with the mean spike time, while single spikes were omitted. As the typical travelling time of an action potential through the microchannel is on the order of 1 ms for the range of axonal conduction velocities reported in microchannels [52, 57], this time window prevents duplicate detection, while still allowing separation of two consecutive spikes. Consistent with this, the temporal gaps between spike events in the same microchannel increase linearly with electrode separation, corresponding to an apparent propagation speed of 0.30 m $s^{-1}$, and 97.7% to 99.8% of them fall within the 1.5 ms window (Figure S39).

#### 4.5.1 Transfer Entropy

Pairwise transfer entropy (TE) was computed between all region pairs of the membrane and water lid conditions at lags up to 5 ms using 0.5 ms bins. Spike data was aggregated over 6 non‐overlapping 20 min windows spanning a 2 h period centered on each medium exchange event. Each TE value at a certain lag was considered as a directed edge from source to target in the network graph, with the graph nodes corresponding to the 4 regions. An edge therefore describes a directed statistical dependence between two regions at a given lag. As a measure of effective rather than structural connectivity [58], its value is not interpreted as a synaptic strength. Statistical significance of each inferred link was assessed using surrogate testing with 100 surrogate time series. For each network, resulting p‐values were corrected for multiple comparisons using the Benjamini–Hochberg false discovery rate procedure, and links with adjusted p<0.05 were considered significant.

To quantify network reconfiguration induced by medium exchange, stable functional edges were identified as directed connections with raw TE $\geq 1\times 10^{-4}$ present at the same lag in all three pre‐exchange windows. This threshold retains the upper 26% of the significant TE values (Figure S24) and was chosen to exclude weak, noisy edges; results for thresholds between $1\times 10^{-5}$ and $1\times 10^{-3}$, tested with a linear mixed model on the edge‐level fold changes with network as random intercept, are given in Figure S26. Contiguous lags were grouped up to 3 bins (1.5 ms), with the requirement that at least one lag within the band exceeded the threshold. For each stable edge, the mean TE across the three post‐exchange windows was compared to the mean TE across the three pre‐exchange windows by computing the log_2_ fold change, using a floor value of $1\times 10^{-6}$ to handle cases where post‐exchange TE dropped to zero. Per‐network summaries included the mean fold change per edge and the cumulative (summed) fold change across all stable edges. For the long‐term recordings, the same analysis was applied to 10 min segments taken every 6 h, and each network was compared with the network 6 h earlier and with the networks at all other time points by cosine similarity (Figure S49).

#### 4.5.2 Spike Time Triggered Raster Plots

To visualize network activity over time, relative latency of spikes was obtained by aligning spontaneous activity to the spike timing of a single trigger electrode [13]. STTRPs were generated using Python. The trigger electrode was selected based on the highest spike count, retaining only the last trigger spike when multiple occurred within one analysis window. Spikes were aligned by subtracting both the nearest preceding and following trigger times, then windowed to ± 10 ms relative to triggers. Circular color coding was applied based on electrode angular position from array center. Visualization used 0.1 ms latency bins, 5 s time bins, and color saturation at 10 spikes per bin.

#### 4.5.3 Pattern Detection and Clustering

A series of spikes with a certain spatiotemporal order is termed a sequence, following conventions by [59, 60, 61]. Sequences were identified by detecting at least two region events within a time window of 20 ms in the relative latencies aligned to the most active electrode in a region. Every spike on a trigger electrode is a candidate alignment event; a trigger is kept only if the next spike on the same electrode is more than one window (20 ms) later (retrigger exclusion), so that sequences from one trigger electrode do not overlap. As the same propagation is detected on the trigger electrodes of several regions, the retrigger exclusion was additionally applied to the pooled triggers of all four trigger electrodes, so that the retained sequence windows are disjoint and no spike contributes to more than one sequence (deduplication). Each sequence was aligned to the first event in the region of the lowest index and transformed into a matrix of 40 time bins of 0.5 ms spanning ± 10 ms around this reference. The window length was set from the region‐event rate triggered on the alignment events, whose excess over baseline has decayed before ± 10 ms (Figure S41). Region events were convolved with a Gaussian kernel of σ=0.75 ms to account for jitter of transduction latencies and avoid dissimilarity between sequences with shared structure. The kernel width was checked against the timing jitter between repeats of a pattern, measured as the difference in timing of corresponding region events between each sequence and its nearest neighbor; this jitter has a robust spread of 0.31 ms, well below the kernel width (Figure S40). Principal component analysis (PCA) was applied to the flattened matrices; the first five components were retained, preserving 64% of the variance.

We use the term pattern for a collection of similar sequences, identified through clustering. Clustering was performed on the five‐dimensional PC space using HDBSCAN [62], a density‐based hierarchical clustering algorithm that infers cluster count from the data density structure, with a minimum cluster size of 2000 sequences and a minimum of 50 sequences required to define a core point of a cluster. Cluster assignment for the full dataset was performed by nearest‐centroid projection from a representative subsample of 500 000 sequences used to fit the model. A total of 46 clusters were identified. For visualization, a 2D t‐SNE embedding was computed on an equal‐per‐cluster subsample of up to 5000 sequences per cluster, ensuring that dominant clusters did not disproportionately determine the embedding layout.

### 4.6 Use of Generative AI and AI‐Assisted Technologies in the Writing Process

Claude (Sonnet 4.5, Sonnet 4.6, Opus 4.5, and Opus 4.6; Anthropic, San Francisco, CA, USA) was used for language editing of the manuscript text and as a coding assistant. All outputs were reviewed, verified, and edited by the authors. No AI tool was used for data collection, interpretation of results, or formulation of scientific conclusions.

## Author Contributions

**Benedikt Maurer**: conceptualization, methodology, software, validation, formal analysis, investigation, data curation, writing – original draft, writing – review & editing, visualization, supervision. **Fabio Fischer**: Methodology, validation, investigation, writing – review & editing, visualization. **Giulia Amos**: Software, validation, investigation, writing – review & editing, supervision. **Vaiva Vasiliauskaitė**: methodology, software, validation, formal analysis, writing – review & editing. **János Vörös**: conceptualization, methodology, writing – review & editing, supervision, project administration, funding acquisition.

## Funding

This research was supported by ETH Zurich, and the Swiss National Science Foundation (SNSF) [project numbers 182779 and 10001282].

## Ethics Statement

Ethical approval was not required for this study. All experiments were performed using human iPSC‐derived neurons obtained from Novartis (Basel, Switzerland). No animal experiments were conducted.

## Conflicts of Interest

The authors declare no conflicts of interests.

## Supporting information


**Supporting File**:advs78463‐sup‐0001‐SuppMat.pdf.

## Data Availability

Manufacturing files and assembly instructions for the lid and inkudock are available at https://doi.org/10.3929/ethz‐c‐000797812 under the Creative Commons Attribution–ShareAlike 4.0 International licence (CC BY‐SA 4.0). Electrophysiology data and analysis scripts are available at https://doi.org/10.3929/ethz‐c‐000798160. The software for inkudock is available on GitHub https://github.com/maurer‐lbb/inkube_software.git.
