# Supplementary material for: A Water Compartment Cell Culture Lid Enables Stable Longitudinal Recording of Neuronal Networks In Vitro
Source: Adv Sci (Weinh). 2026 Nov 3:e78463. Online ahead of print. doi: 10.1002/advs.78463 (PMC13731081; doi:10.1002/advs.78463)
Supplement: Supplementary file 1 [file ADVS-9999-e78463-s001.pdf]

Supplementary Information: A Water Compartment Cell Culture Lid Enables Stable Longitudinal Recording of Neuronal Networks in Vitro

Benedikt Maurer[1], Fabio Fischer[1], Giulia Amos[1], Vaiva Vasiliauskaitė[1], János Vörös[1*]

[1*]Laboratory of Biosensors and Bioelectronics, Institute for Biomedical Engineering, University and ETH Zurich, Zurich, Switzerland.

*Corresponding author(s). E-mail(s): janos.voros@biomed.ee.ethz.ch;

A Hardware description

A.1 Water compartment lid

A.1.1 Water compartment lid with fluidics and sensor

The water compartment cap, illustrated in Fig. S1, comprises six custom-designed components (C1-C6 in Fig. S1), each CNC-milled from either polyether-ether-ketone (PEEK) or polymethyl methacrylate (PMMA), depending on sterilisation and functional requirements. The components were assembled using Araldite 2011 (Huntsman Corporation, The Woodlands, Texas, USA), a two-component epoxy adhesive selected for its mechanical strength and chemical stability. A comprehensive list of all components is available in Table S1.

There are 2 membranes (M1, M2 in Fig. S1) positioned above the chip well. The space between the membranes is filled with ultrapure water (Milli-Q, Millipore-Sigma, Darmstadt, Germany) and includes an air gap (Fig. S3). To secure the upper membrane, a thin ring (C3 in Fig. S1) is pushed down into the water compartment.

To enable liquid handling within the well while the cap is applied, three stainless-steel needles are integrated into the design: two inlet needles (I in Fig. S1) and one extraction needle (E in Fig. S1). To maximise gas exchange efficiency, the air–liquid

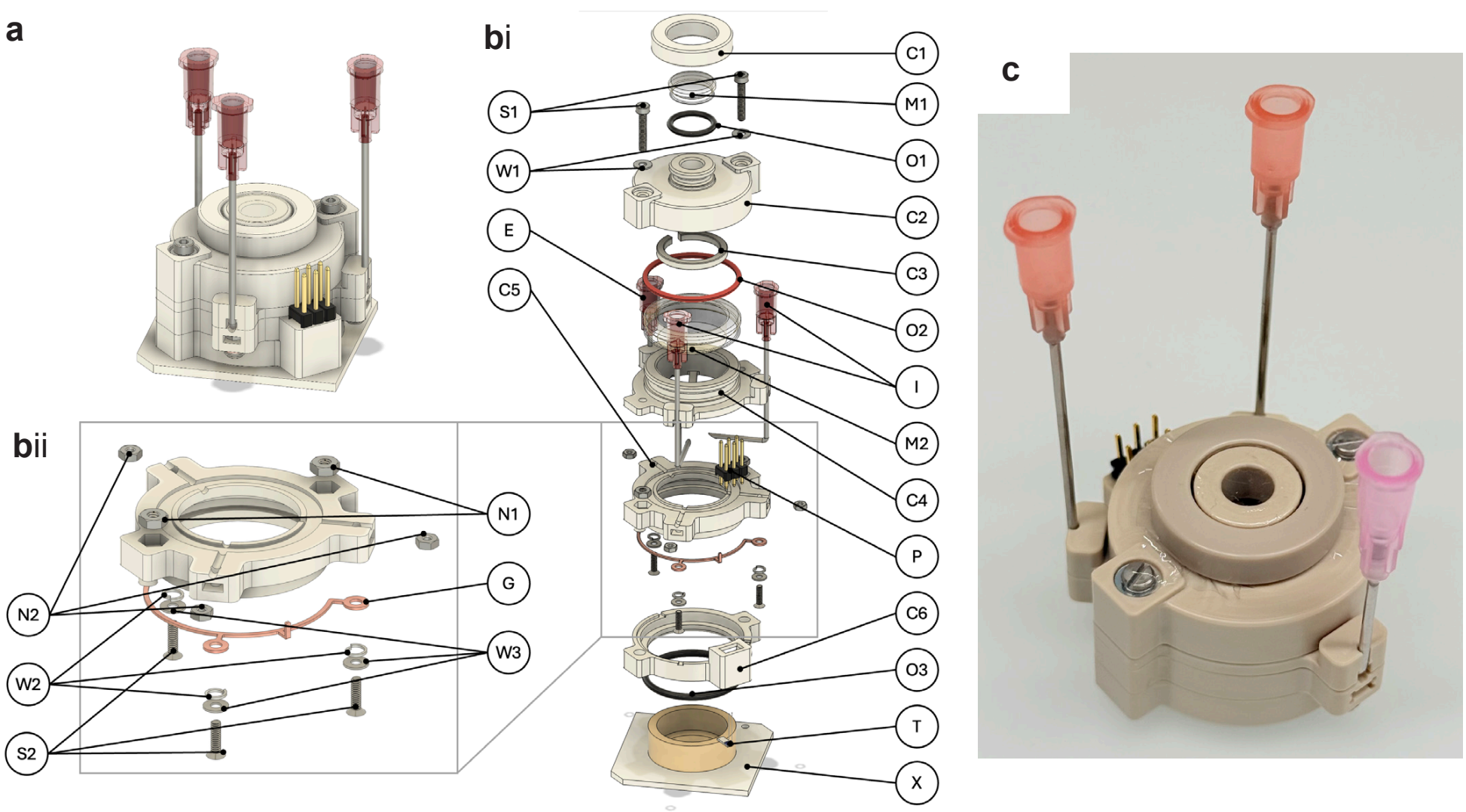

Fig. S1: **CAD design and photo of water compartment lid. a**, CAD-rendered overview of the water compartment cap, showing the complete assembly. **b**, (i) Exploded view with (ii) grounding plate details. The lettering corresponds to the label IDs listed in Table S1. **c**, Photograph of the assembled CMOS Cap manufactured from PEEK.

interfacial area of the water compartment is designed to be as large as possible. Considering the limited spatial clearance within the inkudock chamber (Fig. S3), the needles are bent upward using a custom bending rig.

To improve needle mechanical stability, the middle component (C4 in Fig. S1) contains snap-fit features that securely hold the needles in two positions. This configuration facilitates assembly and improves robustness during operation. The inlet needles are bent to avoid direct contact with the culture medium, thereby minimising undesired diffusion of compounds through the fluidic system. In contrast, the extraction needle remains in contact with the medium to allow aspiration from the well (Fig. S3).

A grounding plate (G in Fig. S1) is fabricated by laser-cutting a 0.45 mm thick copper sheet into the desired outline. After bending to its final geometry, the plate is integrated into the lower part of the cap and soldered to the connector (P in Fig. S1), as illustrated in Fig. S2. Electrical contact between the grounding plate and the needles is established using screws, washers, and spring washers (S2, W3, W2 in Fig. S1).

For monitoring chip surface temperature, a temperature sensor (T in Fig. S1) is integrated into the cap. The mounting geometry for the sensor within the lower bottom component (C6 in Fig. S1) is specifically designed to optimise its thermal response to surface temperature changes on the CMOS chip. Several geometrical configurations were evaluated, varying the protrusion distance with respect to the bottom surface. The fastest response to temperature fluctuations was achieved when the sensor slightly protruded from the surface. During assembly, the sensor is first bonded into the lower

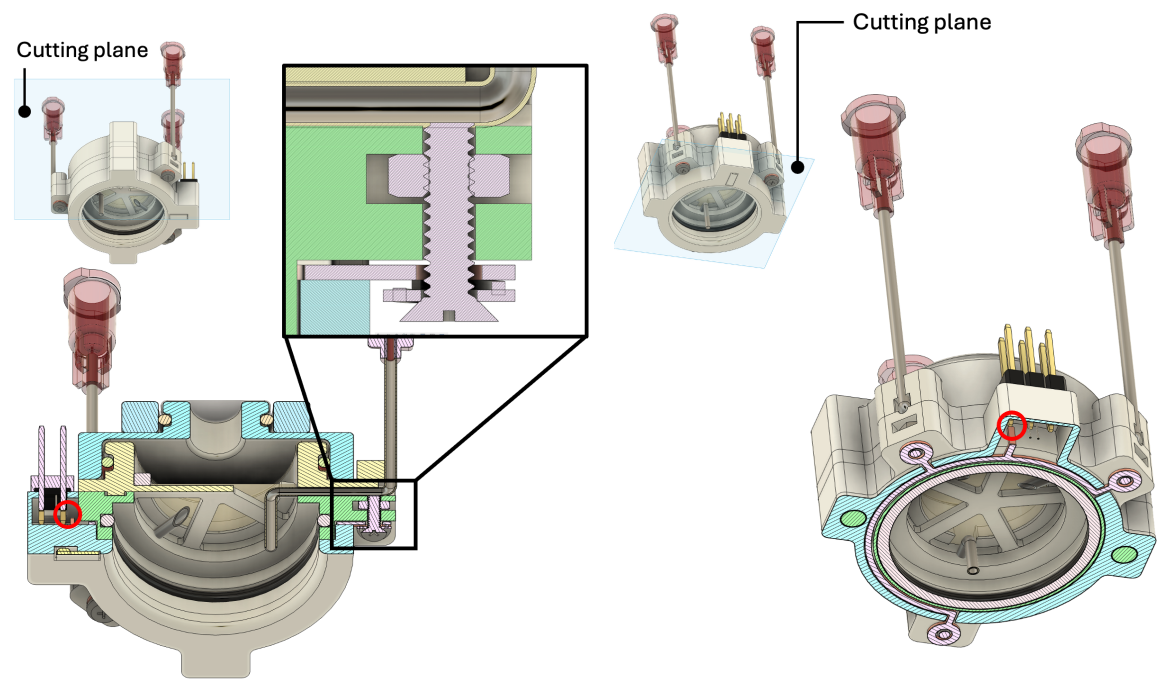

Fig. S2: **CAD renderings of the grounding system shown in two sectional views. The red circles highlight the soldering locations between the grounding plate and the connector.** Left: Vertical section with detail view demonstrating the electrical connection between the needles and the grounding plate established by M1.6 screws. Right: Horizontal section illustrating the position of the grounding plate.

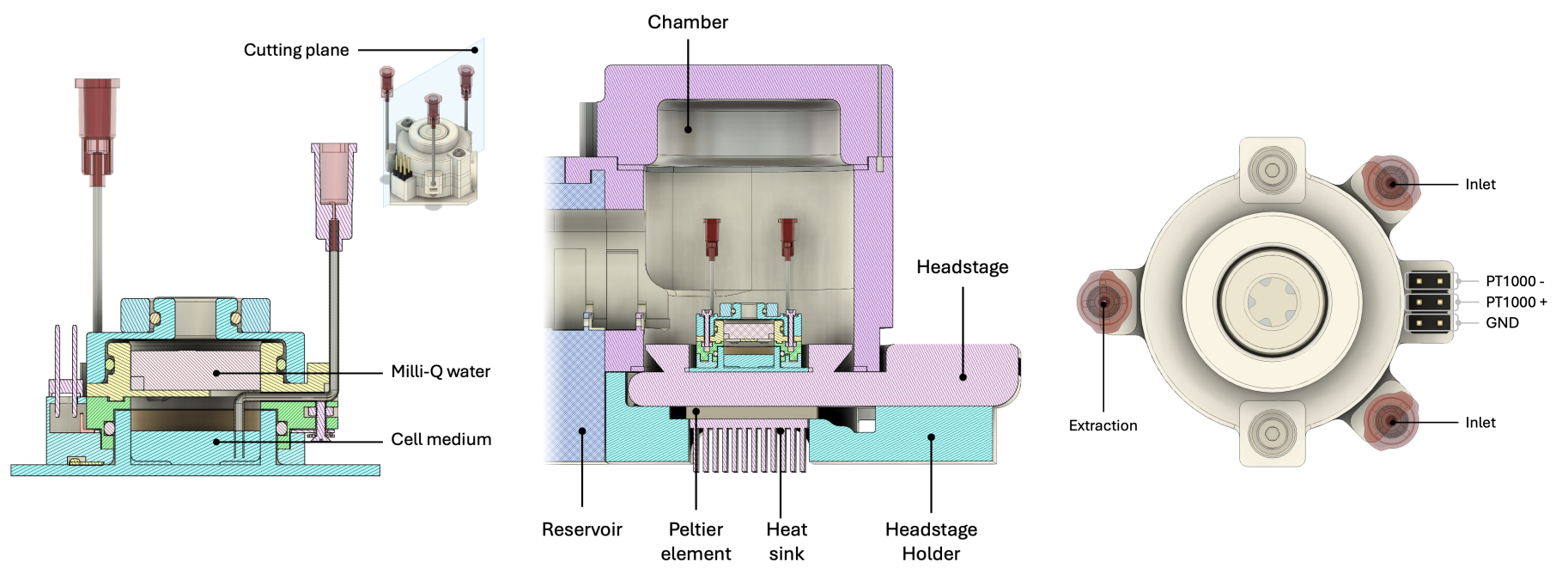

Fig. S3: **Detail views of the water compartment lid and setup.** Left: Sectional CAD view of the water compartment cap mounted on a CMOS chip. Centre: Sectional CAD view of an inkudock chamber containing a CMOS chip with the water compartment cap installed. Right: Top-view CAD rendering of the water compartment cap illustrating the arrangement of liquid-handling and electrical connections.

bottom component using adhesive and subsequently soldered to the connector (P in Fig. S1). The corresponding pin assignments for all components are shown in Fig. S3.

To ensure an airtight seal between the interior of the CMOS chip well and the external environment, a combination of adhesive bonding and O-rings is employed. Adhesive is applied along the glue ridge of the upper-bottom component (C5 in Fig. S1), encapsulating sections of each needle in epoxy and thereby forming a robust, leak-tight interface. The membranes are sealed using o-rings (O1, O2 in Fig. S1). The top ring (C1 in Fig. S1) and top component (C2 in Fig. S1) are compressing the o-rings to achieve a gas-tight seal. Finally, the interface between the CMOS chip well

and the water compartment cap is sealed by a press-fit o-ring (O3 in Fig. S1), ensuring complete isolation of the interior of the CMOS chip well.

Stepwise instructions with detailed depictions can be found in the Assembly Instructions.

A.1.2 Simplified water compartment HD-MEA lid

For applications in the standard incubator without recording, for short-term recordings, or in combination with the heated top component (C10 in Fig. S5, see Section A.1.3), the design of the water compartment cap is adapted into a simplified version that omits both the temperature sensor and the liquid-handling needles (Fig. S4). Consequently, a grounding system is no longer required. These simplifications enable a fully mechanical assembly that eliminates all glueing steps, thereby simplifying fabrication and maintenance. While the middle component and the top ring are interchangeable with those of the standard cap, the top component is unique, as the simplified version is also used with standard glass-based MEAs, requiring a reduction in the overall outer dimensions to fit the recording setup.

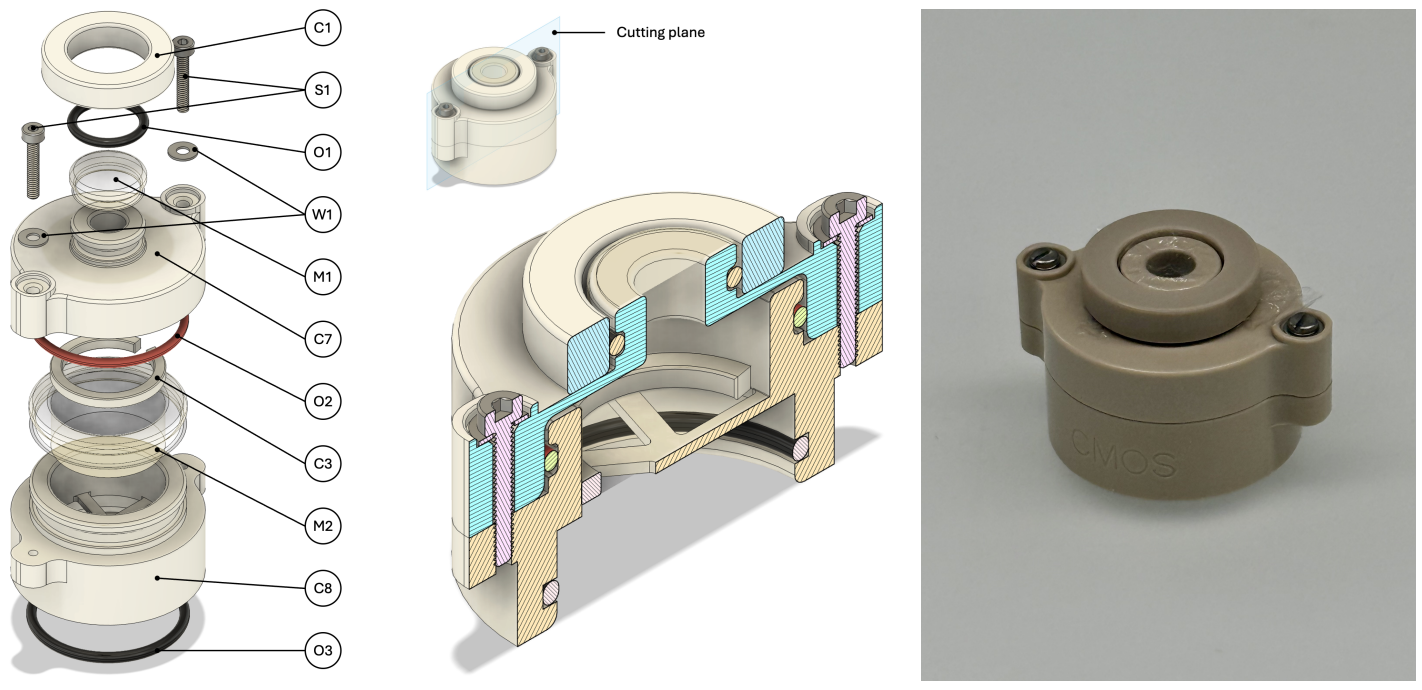

Fig. S4: **CAD design and image of the simplified MEA lid.** Left: Exploded CAD view of the simplified water compartment cap without temperature sensor or liquid-handling needles, showing all major components and O-ring placements. Centre: Section view of the assembled cap illustrating the sealing interfaces and internal geometry. Right: Photograph of the assembled cap manufactured from PEEK. To differentiate it from other cap versions, "CMOS" is engraved laterally.

A.1.3 Heated water compartment HD-MEA lid

To replicate the temperature gradient achieved in inkudock, where reservoir heating and recording unit temperature control are combined, within a standard incubator, a heated variant of the top component was developed for use with the simplified water compartment lid (Section A.1.2). A resistor (R in Fig. S5) and temperature sensor (T in Fig. S5) are embedded within a protrusion of the heated top component that extends into the water compartment, enabling active temperature control of the water. In all

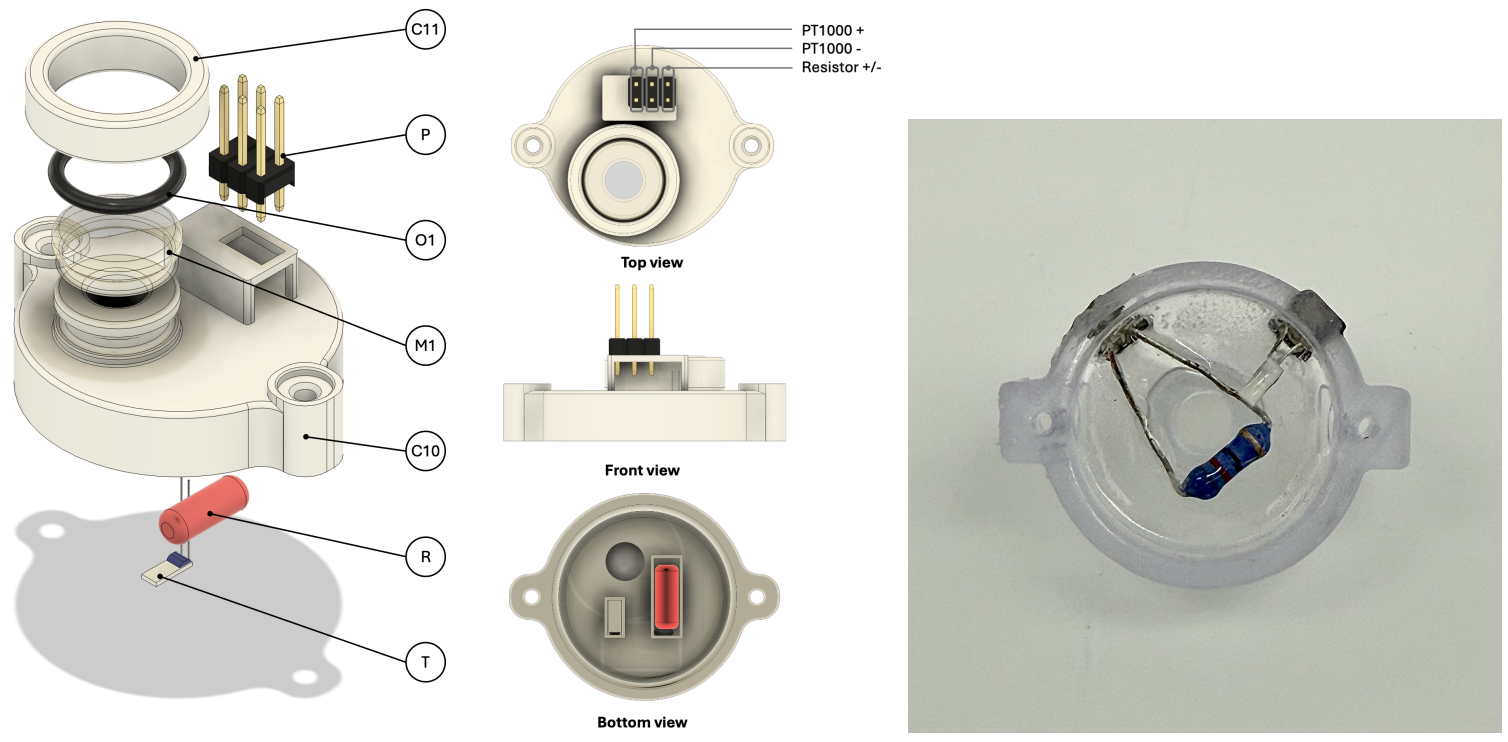

Fig. S5: **CAD design and first prototype image of the heated water compartment lid.** Left: Exploded CAD view of the heated water compartment cap, showing all major components and sealing elements. Centre: Different views of the assembled cap illustrating the internal geometry. Right: Photograph of the assembled top part 3D-printed from resin.

experiments, the water compartment was maintained at 40 °C while the incubator was set to 35.5 °C. The component was fabricated using resin-based 3D printing (Formlabs, Somerville, MA, USA). The prototype used in experiments (Fig. S5) differs from the final CAD design in the mechanical support and sealing of the embedded components: in the prototype, the resistor and sensor were suspended without structural support and protected only by a thin layer of Araldite 2011, whereas the final design fully encapsulates them for protection of the access wires.

A.1.4 Water compartment glass-MEA lid

The water compartment cap was further adapted for use with standard passive glass-based MEAs. While the MEA well (60MEA500/10iR-Ti-gr, Multi Channel Systems MCS GmbH, Reutlingen, Germany) has the same outer diameter as the MaxOne, its lower height required an adjustment of the bottom component geometry. The middle ring, top, and top ring are compatible with the simplified water compartment cap, ensuring design consistency and simplifying fabrication.

A.1.5 Leakage test lid

The leakage test cap is identical in design to the water compartment cap, except that instead of a middle membrane (M2 in Fig. S1), Middle Ring (C3 in Fig. S1) and a water layer, it contains a solid block of material (C12 in Fig. S6).

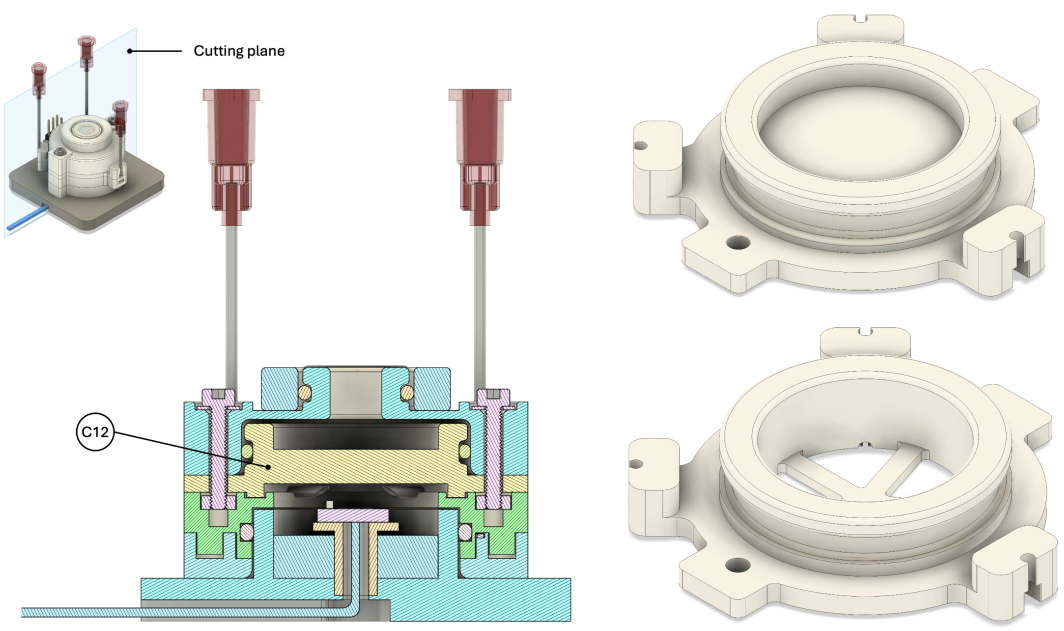

Fig. S6: **CAD design of the cap used for leakage test.** Left: Section view of the CAD model of the leakage test cap. Right: CAD Rendering of the Verification Middle (C12) on the top and the regular Middle Component (C4 in Fig. S1) on the bottom.

A.2 Custom incubator inkudock

A.2.1 Overview

The block diagram in Fig. S7 shows the different components and their respective boards.

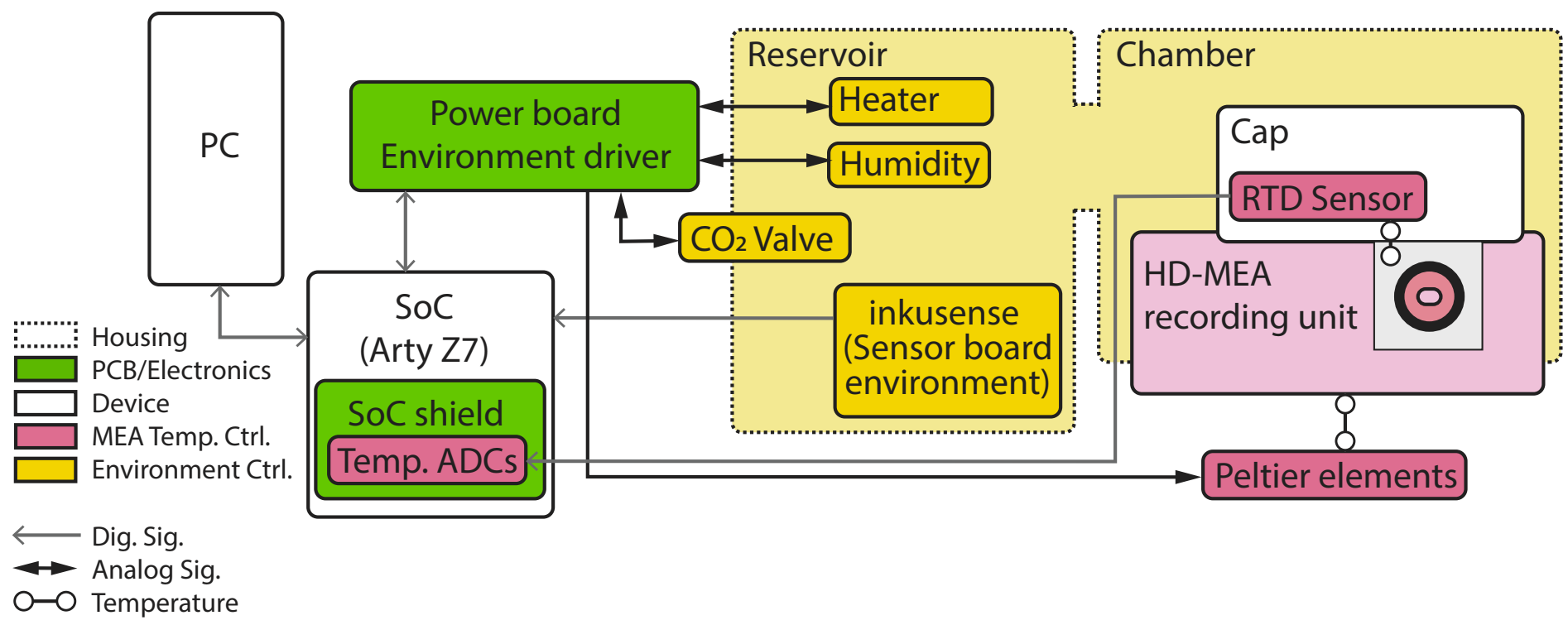

Fig. S7: Block diagram of inkudock. The PCBs are adapted from inkube. All active components for environment control are placed inside the reservoir.

A.2.2 Housing

Fig. S8A shows an annotated picture of inkudock with partially mounted chambers.

Reservoir

The reservoir, depicted in Fig. S8A, carries the sensors and active components for temperature and CO_2 control, as well as fans to equilibrate the housing inside. A passive water bath can be used to humidify the environment. The assembly is presented in Fig. S9 and Fig. S10.

Chamber

The chamber carries no active components. It encloses the HD-MEA and connects to the reservoir, as shown in Fig. S8B.

Holder

On the holder, the Peltier device is embedded. On top the recording unit is placed. The fan is mounted at the end of the ventilation channel. Details are shown in Fig. S11.

A.2.3 Electronics

The electronics are based on the inkube setup Maurer et al. (2026). Two custom printed circuit boards (PCBs) are embedded in the setup: the power board and the

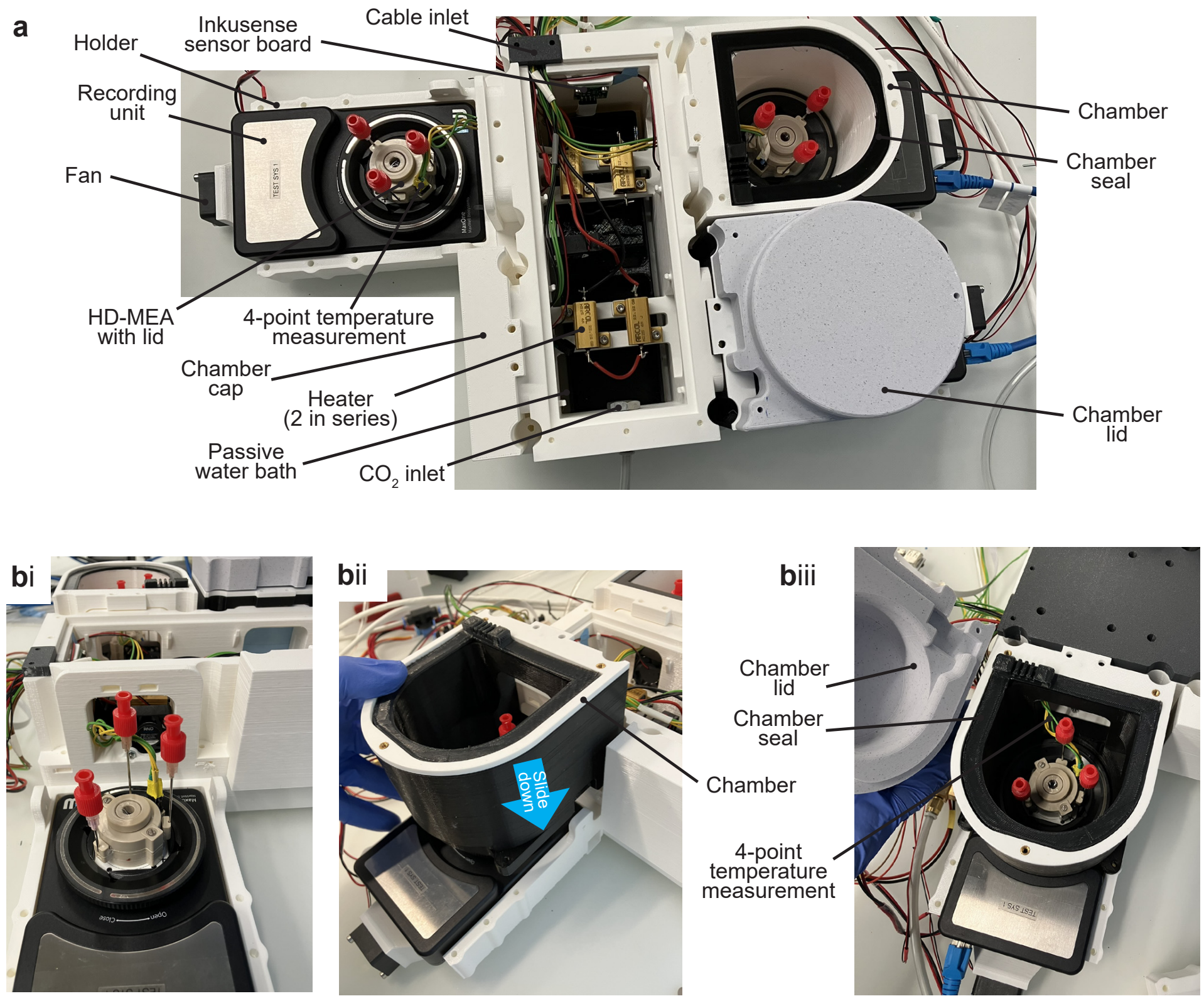

Fig. S8: **inkudock overview a**, Top view of the assembled inkudock with one closed chamber on the bottom right, a chamber with the lid removed on the top right, a recording unit without the chamber on the top left, and a closed connection port on the bottom left. **b**, Chamber mounting. **(i)** Recording unit with mounted chip aligned to a port. **(ii)** The chamber can be mounted by sliding in from the top to hold the recording unit in place and connect the chamber interior to the reservoir. **(iii)** Finally, the chamber seal is placed, and the lid can be attached.

extended shield. The power board is a revised version of the inkube_pwr. Besides a few minor fixes, the humidity and reservoir heater have been changed to single-ended FET-based output drivers. The extended shield is a combination of the previous inkube_fpga and inkube_bot. It houses the temperature measurement and relays the power board and SoC signals.

Besides the custom PCBs, one replicate of the inkusense board is required for control of the reservoir Duru et al. (2024). As heaters, 2 pairs of 2 resistive heaters connected in series are used (RH). For heating and cooling the recording unit, 2 Peltier devices are connected in series (TD) and attached to a heat sink (HS) with thermally conductive foil (HF). Further, 2 fans per mounted recording unit help distribute the air inside the reservoir and chamber and dissipate heat from the heat sink (F).

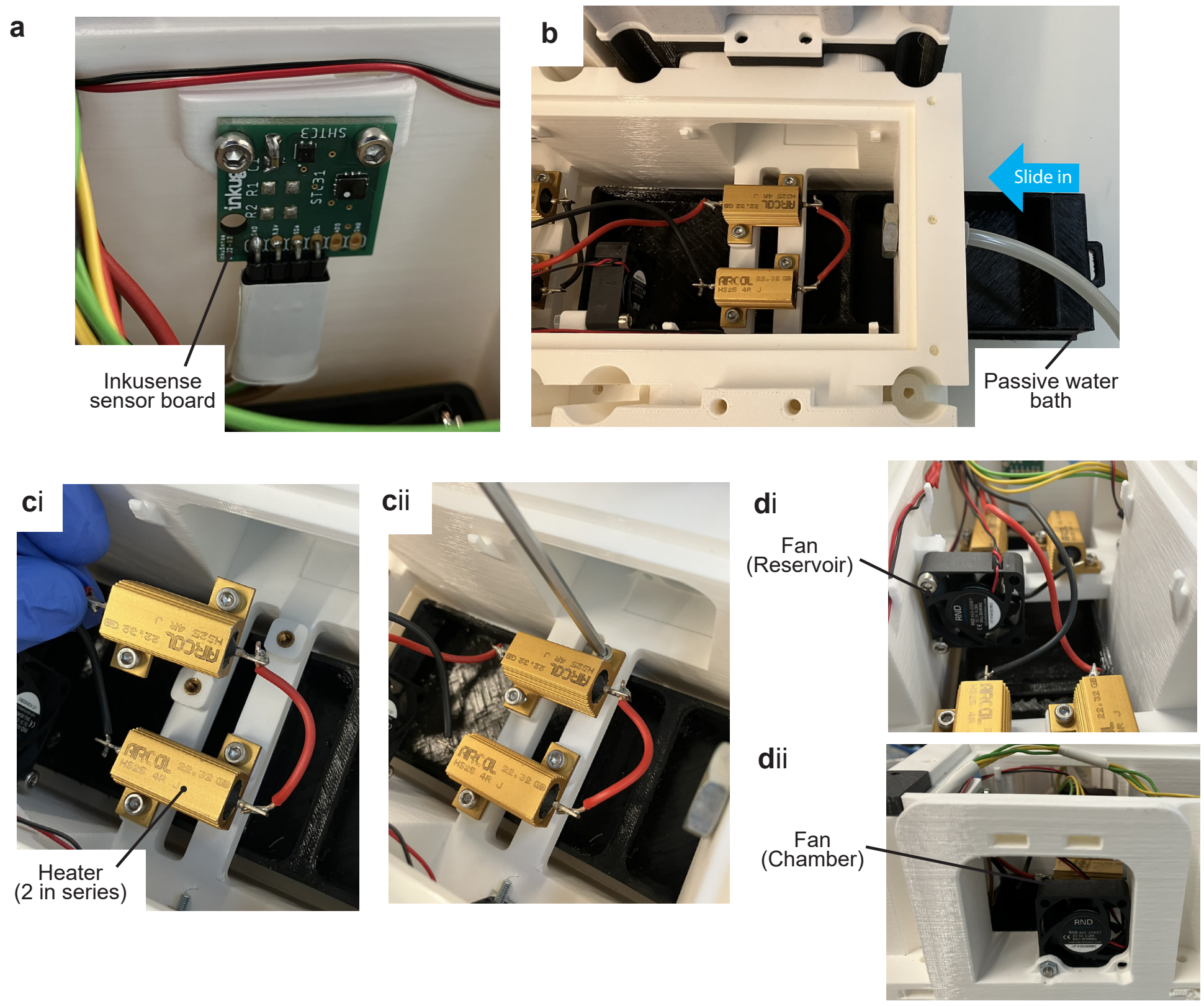

Fig. S9: **Mounting of components in the reservoir. a**, The inkusene board is attached with 2 M2.5x6 screws with counter hex nuts. **(i)** The water bath is slid in from the side. **c**, Mounting the resistive heaters. **(i)** For heating of the reservoir, four 4 Ω resistive heaters (RH in S2) are placed, split into 2 branches of 2 resistors in series. **(ii)** Two M3 thread inserts per resistor are placed in the holes and the resistors are mounted using 2 M3x8 screws. **d**, Fan mounting. **(i)** One central fan (F in S2) is placed in the reservoir, with the airflow pointing towards the CO_2 inlet, by using a M3x18 screw and a counter hex nut. **(ii)** Per chamber, one fan (F in S2) is used to balance temperature gradients and attached to the inkudock port with one M3x20 screw.

A.3 Validation sensing setups

A.3.1 Sensor dummy chip

To evaluate the CO_2 diffusion characteristics of the water compartment cap, a custom test setup (hereafter referred to as the *test well*) was fabricated. The test well replicates the geometry of the CMOS chip and includes a central platform that holds a sensor board capable of recording relative humidity, temperature, and CO_2 concentration. The sensor board was developed in a previous project where it was used to control an incubator setup Duru et al. (2024). The sensor board cables are routed through a hole

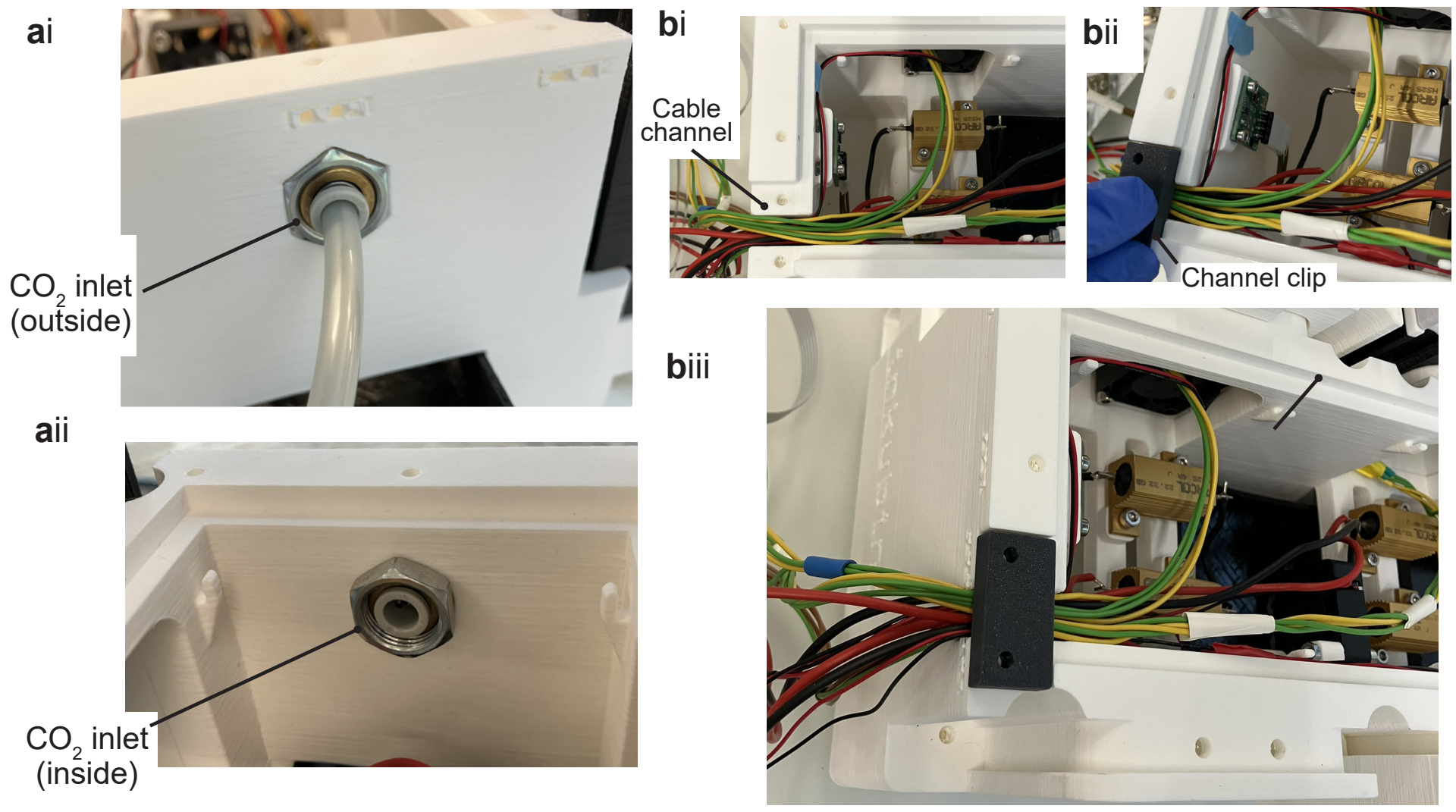

Fig. S10: Mounting inlets and outlets of the inkudock. **a**, CO_2 connection. **(i)** The transduction thread is mounted from outside into the hole. The tube supplying the CO_2 from the pulsing valve can be plugged in. **(ii)** The thread is secured from the inside with a counter hex nut. **b**, All cables leading inside the inkudock are aligned to the cable channel next to the sensor board. **(ii)** and **(iii)** A clip is used to secure the cables.

in the platform and well and sealed with Araldite 2011 adhesive to ensure an airtight connection (Fig. S12).

A.3.2 Sensor ring

The sensor ring incorporates the same PT1000 embedding design as the water compartment lid, but features a reduced structure around the well, forming a thin ring that allows a commercial single-membrane cap to be placed on top (Fig. S13).

A.4 Component and Supplier Details

Tables S1 and S2 summarise the components used in the water compartment cap and the inkudock incubator, respectively. PCB design files, 3D CAD models, and detailed component-level BOMs are available in the ETH Research Collection (see data and material availability statement).

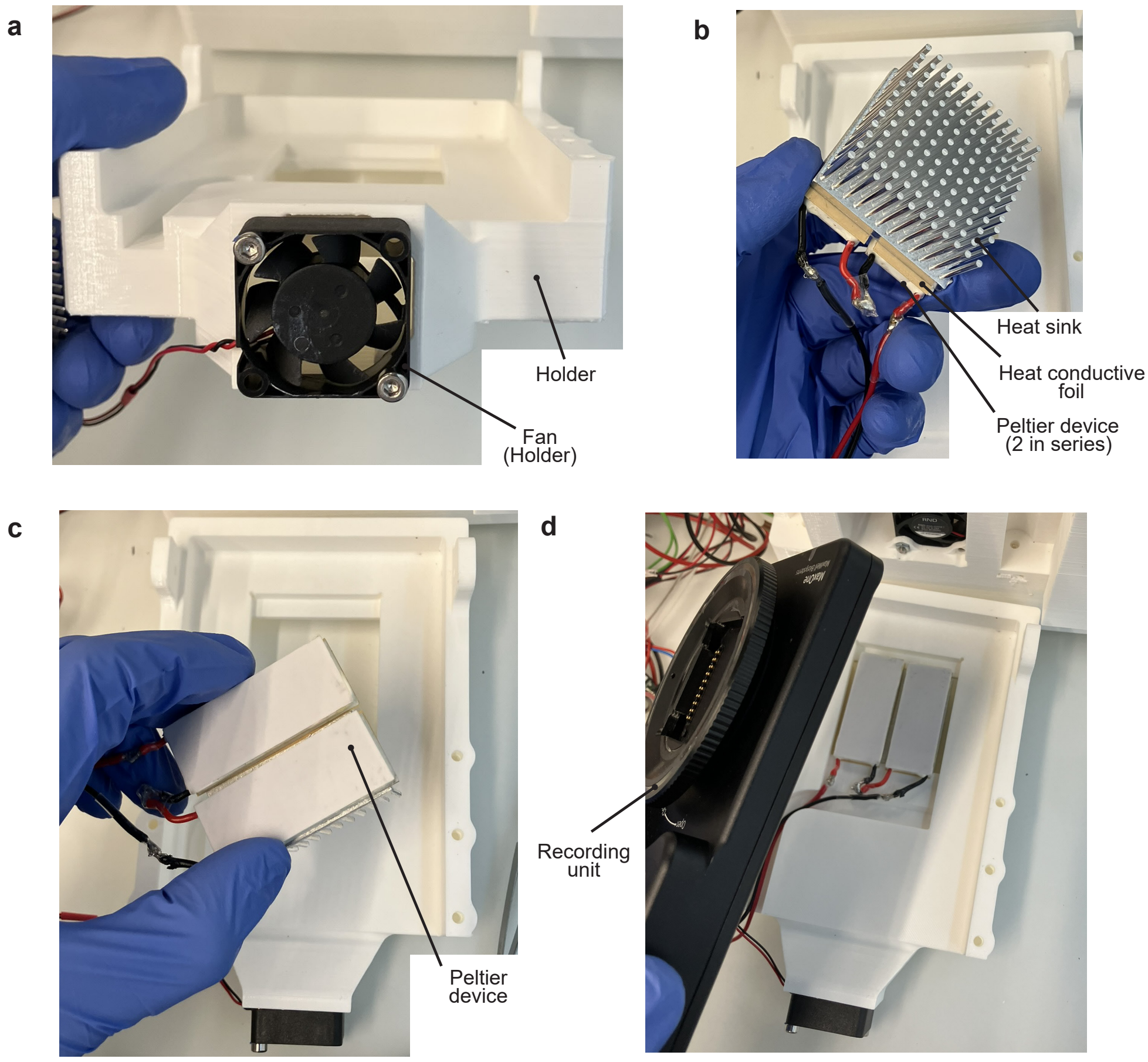

Fig. S11: **Assembly of the holder. a**, The fan (F in S2) is mounted at the end of the ventilation channel, with 2 M3x15 screws and M3 thread inserts, pointing its airflow towards the Peltier device. **b**, Peltier device are mounted with a thermally conductive adhesive foil on top of a heat sink, with the labelled side of the Peltier devices exposed towards the top. For sufficient power, 2 Peltier devices are connected in series (be aware of the polarity). **c**, The assembly is then placed in the gap of the holder and the cables are lead out. **d**, Finally, the MaxOne recording unit is placed on top of the holder.

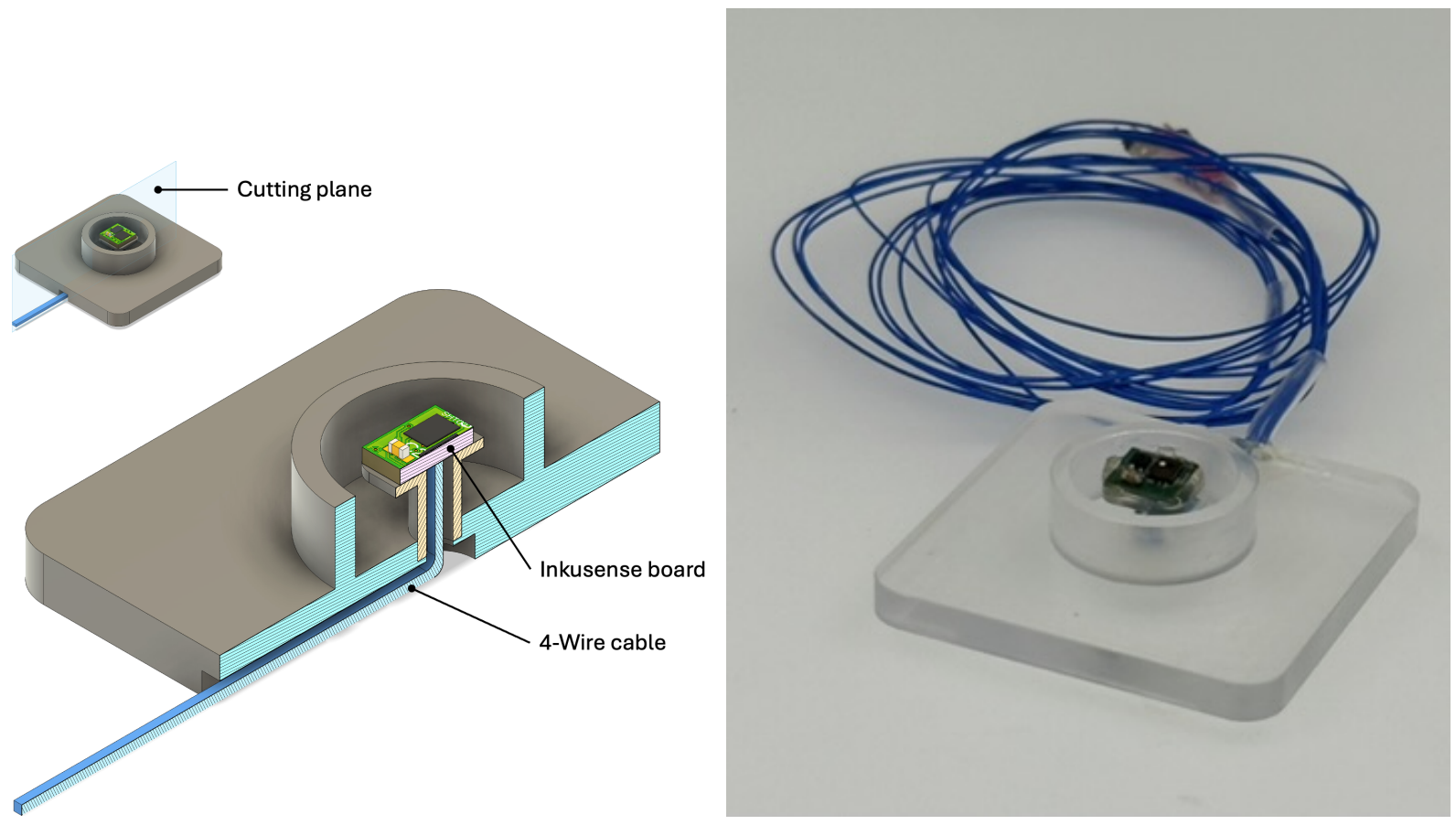

Fig. S12: Left: Section view of the CAD model of the test well illustrating the well geometry and chamber configuration. Right: Photograph of the test well fabricated from PMMA.

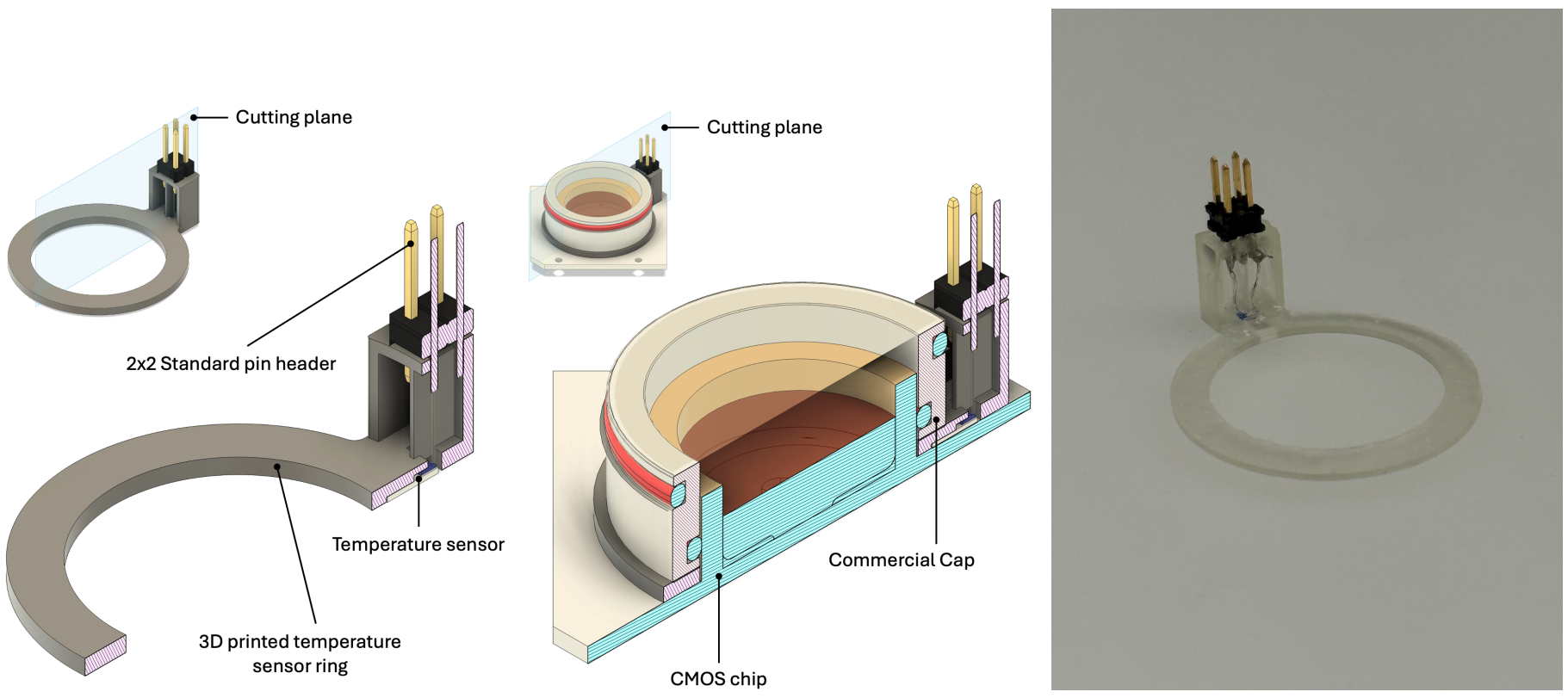

Fig. S13: Left: Section CAD view of the sensor ring. Centre: Section CAD view of the sensor ring mounted on a CMOS chip with a single-membrane lid mounted on top of it. Right: Photograph of the sensor ring.

**ID**	**Name**	**Specification**	**Manufacturer / Supplier**
Cap Parts (CNC machined)			
C1	Top Ring	PEEK / PMMA	In-House
C2	Top	PEEK / PMMA	In-House
C3	Middle Ring	PEEK / PMMA	In-House
C4	Middle	PEEK / PMMA	In-House
C5	Upper Bottom	PEEK / PMMA	In-House
C6	Lower Bottom	PEEK / PMMA	In-House
C7	Top Glass MEA	PEEK / PMMA	In-House
C8	Bottom CMOS NS/NT	PEEK / PMMA	In-House
C9	Bottom Glass MEA	PEEK / PMMA	In-House
C12	Verification Middle	PMMA	In-House
Cap Parts (3D printed)			
C10	Heated Top	BiomedClear Resin	In-House
C11	Heated Top Ring	BiomedClear Resin	In-House
Seals and Membranes			
O1	Top O-Ring	Viton 75 Shore, 10×1.5 mm (0103-004528)	Kubo, CH
O2	Middle O-Ring	Viton 75 Shore, 23×1.5 mm (0103-212057)	Kubo, CH
O3	Bottom O-Ring	MVQ 70 Shore, 24×2 mm (0102-194567)	Kubo, CH
M1	Small Membrane	ALA MEA-MEM-SHEET, 50 Gauge	MCS GmbH, DE
M2	Large Membrane	ALA MEA-MEM-SHEET, 50 Gauge	MCS GmbH, DE
Fasteners			
S1	M2×12 Screw	Pan Head, Slotted, V4A SS	toolcraft AG, DE
S2	M1.6×6 Screw	Pan Head, Slotted, A2 SS	RS Components, CH
N1	M2 Nut	SS, DIN 934	RS Components, CH
N2	M1.6 Nut	A2-304 SS, 3.20 mm, DIN 934	RS Components, CH
W1	M2 Washer	Stainless Steel	RS Components, CH
W2	M2 Spring Washer	Zn-Plated Steel, 0.5×2.2×4.4 mm	RS Components, CH
W3	M1.6 Washer	Stainless Steel	RS Components, CH
Other Components			
P	Connector	2×3 Pin Header, 2.54 mm pitch	RS Components, CH
I	Inlet Needle	Sterican, 1.2×50 mm, sharp	B. Braun, DE
E	Extraction Needle	Sterican, 1.2×40 mm, blunt	B. Braun, DE
T	Temp. Sensor	PT1000, NB-PTCO-170	TE Connectivity / DigiKey
G	Grounding Plate	Copper Sheet, 300×300×0.45 mm	In-House, laser cut
MEA Systems			
X1	CMOS HD-MEA	MaxOne+ HD-MEA chip	MaxWell Biosystems, CH
X2	Recording system	MaxOne recording system	MaxWell Biosystems, CH

Table S1: Bill of materials for the water compartment cap.

ID	Name	Specification	Manufacturer / Supplier
Reservoir (3D printed, PLA)			
CAD1	Reservoir housing	PLA, 3D printed	In-House
CAD2	Reservoir lid	PLA, 3D printed	In-House
CAD3	Water bath	PLA, 3D printed	In-House
CAD4	Reservoir seal	PLA, 3D printed	In-House
CAD5	Port cap	PLA, 3D printed, seals unused chamber ports	In-House
CMOS MEA Chamber and Recording Holder (3D printed, PLA)			
CAD6	Recording holder	PLA, 3D printed	In-House
CAD7	Chamber	PLA, 3D printed	In-House
CAD8	Insulation chamber	TPU, 3D printed	In-House
CAD9	Chamber lid	PLA, 3D printed	In-House
Printed Circuit Boards			
PCB1	Power board	4-layer, power_board v4.4	Custom, see Research Collection
PCB2	Extended shield	2-layer, extended_shield v4.4	Custom, see Research Collection
PCB3	Inkusense board	2-layer, inkusense v4.2	Custom, see Duru et al. (2024)
Electronic Components			
SoC	FPGA/SoC board	Arty Z7-20, Zynq-7000 Z7020 (410-346-20)	Digilent / DigiKey
PSU	Power supply	DT80PW090D, 9 V	TDK-Lambda / DigiKey
Thermal Management (per holder)			
TD	Peltier device	TEC1-07901, 50×20 mm (×2, series)	Wellen Technology Co., China
HS	Heat sink	ICK S 45×45×20, pin type	Fischer Elektronik, DE
HF	Thermal foil	WLFT 404, 40×40 mm	Fischer Elektronik, DE
F	Fan	RND 460-00087, 30×30×10 mm, 5 V (×2)	Distrelec AG, CH
Environment Control			
RH	Resistive heater	HS25 4R J (×4, 2×2 series)	ARCOL / Distrelec
V	CO_2 valve	PVQ13-6M-06-M5-A	SMC Pneumatics / Distrelec

Table S2: Bill of materials for the inkudock incubator.

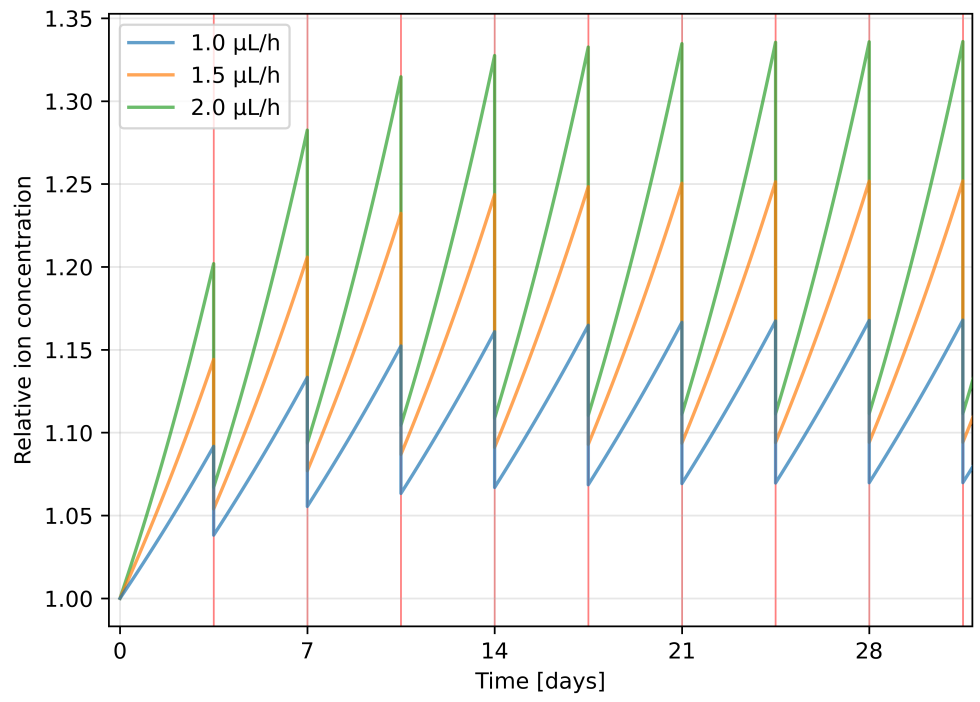

Fig. S14: **Simulated relative ion concentration in the culture medium over time under a half-medium exchange protocol.** Each cycle consists of 3.5 days of evaporation followed by removal of half the well volume (500 µL from a 1000 µL starting volume) and replenishment with fresh medium to restore the original volume. Curves are shown for three constant evaporation rates (1.0, 1.5, and 2.0 µL/h). Medium exchange is indicated with red vertical lines.

B Device characterisation

B.1 Ion concentration change due to half-medium exchange

To estimate the cumulative effect of evaporation on extracellular ion concentrations, we modelled the medium as a discrete recurrence over exchange cycles of 3.5 days. Within each cycle, evaporation removes only water at a constant rate, thereby concentrating all solutes by a factor $V_0/(V_0 - V_{\text{evap}})$, where $V_0 = 1000\,\mu\text{L}$ is the starting volume and V_{evap} is the total volume lost to evaporation per cycle. At the end of each cycle, 500 µL of the remaining medium are removed, and the starting volume V_0 is restored by adding fresh medium (500µL + evaporated volume). For the common evaporation rates in an incubator with a membrane lid (1.0–2.0 µL/h), steady-state concentrations are elevated by at least approximately 7–11 % (right after medium exchange) relative to fresh medium after 30 days.

B.2 Temperature distribution in a conventional recording setup

To compare the temperature gradients of a conventional recording setup with those in inkudock (Supplementary Note B.3), temperature sensors were placed in the housing, in the culture liquid and in the air above the lid, using PT1000 elements and the same measurement pipeline as for inkudock; the liquid sensor was insulated in biocompatible heat-shrink tubing. During the warm-up (Fig. S15), the air above the lid, with negligible thermal mass, reaches within 1 °C of its final value in about 10 min, while the liquid follows the housing over roughly an hour. Once settled (Fig. S16), the housing and the liquid track each other to within approximately 0.05 °C, while the air above the lid sits approximately 1.1 °C below them. The medium is therefore

the warmest element of the assembly, because the culture is heated from below by the power dissipated in the CMOS chip and the recording unit. This is the configuration that drives evaporation from the medium and condensation onto the lid, and it is inverted by the temperature control of inkudock.

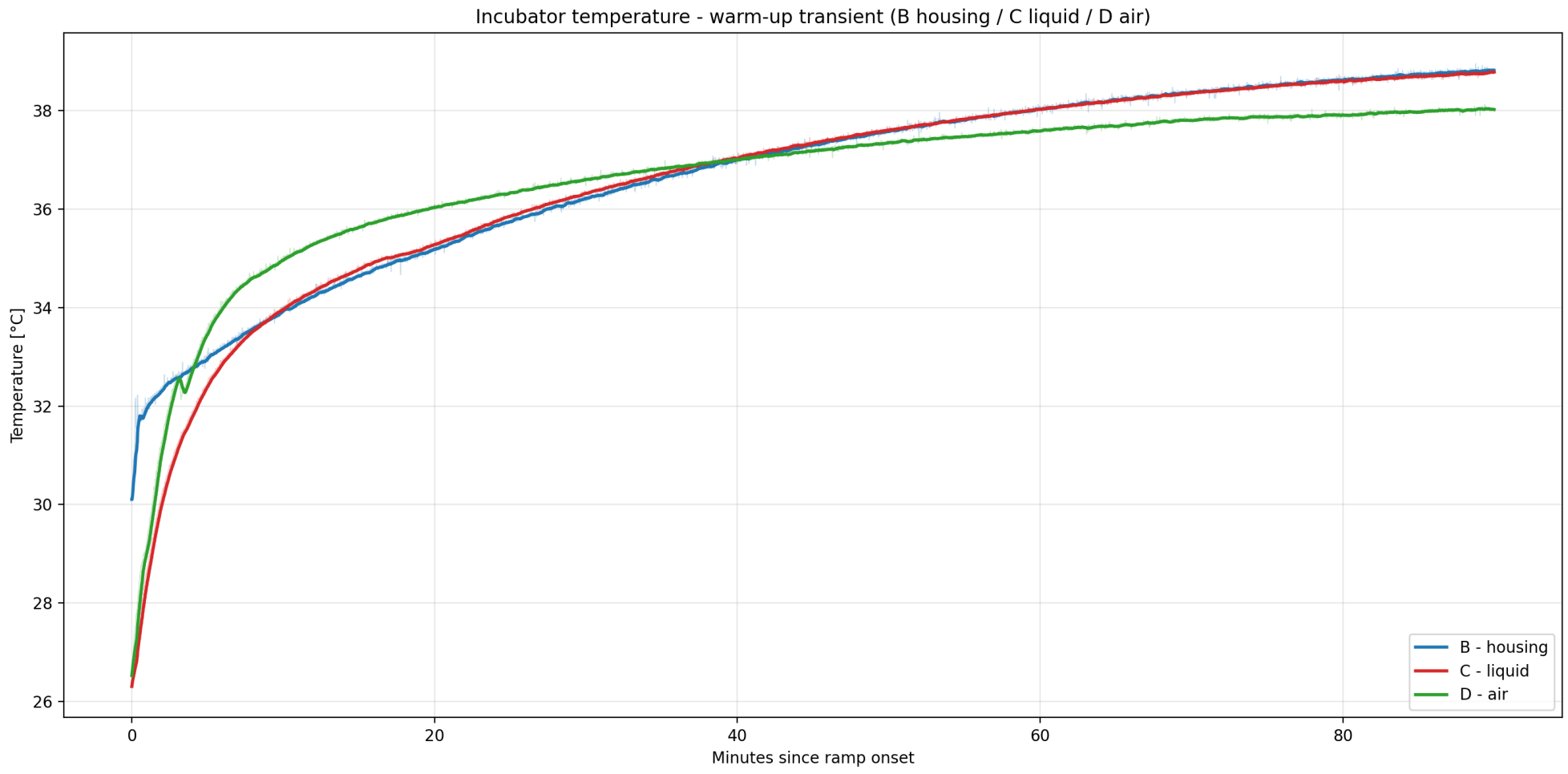

Fig. S15: **Warm-up transient of the conventional recording setup.** Temperature in the housing (B, blue), in the culture liquid (C, red) and in the air above the lid (D, green), from the onset of the temperature ramp, measured with PT1000 elements and the same pipeline used for the inkudock calibration of Fig. S17; the liquid sensor was insulated in biocompatible heat-shrink tubing. The air above the lid, having negligible thermal mass, rises fastest and settles first, while the liquid follows the housing over approximately one hour.

B.3 Temperature calibration

Measurements during calibration of the temperature control loop showed a chamber temperature of approximately 43 ° C when the reservoir was set to 45 ° C (Fig. S17C). To achieve physiological conditions for the cells, the relation between medium and HD-MEA surface temperature was determined. The sensor in the culture medium was therefore controlling the Peltier device with a set temperature of 35.5 ° C, which was established to correspond to 38.5 ° C on the HD-MEA surface. The fact that the set temperature is higher than the medium temperature is presumably explained by the sensor placement. As the sensor is mounted on the lid and pressed onto a corner of the MEA chip, it is still strongly influenced by the temperature of the chamber environment. The recording unit, in contrast, is presumably colder as the bulk of the material is outside of the chamber, and the lid serves as a good insulator. This would then lead to a temperature gradient across the HD-MEA, with a lower temperature

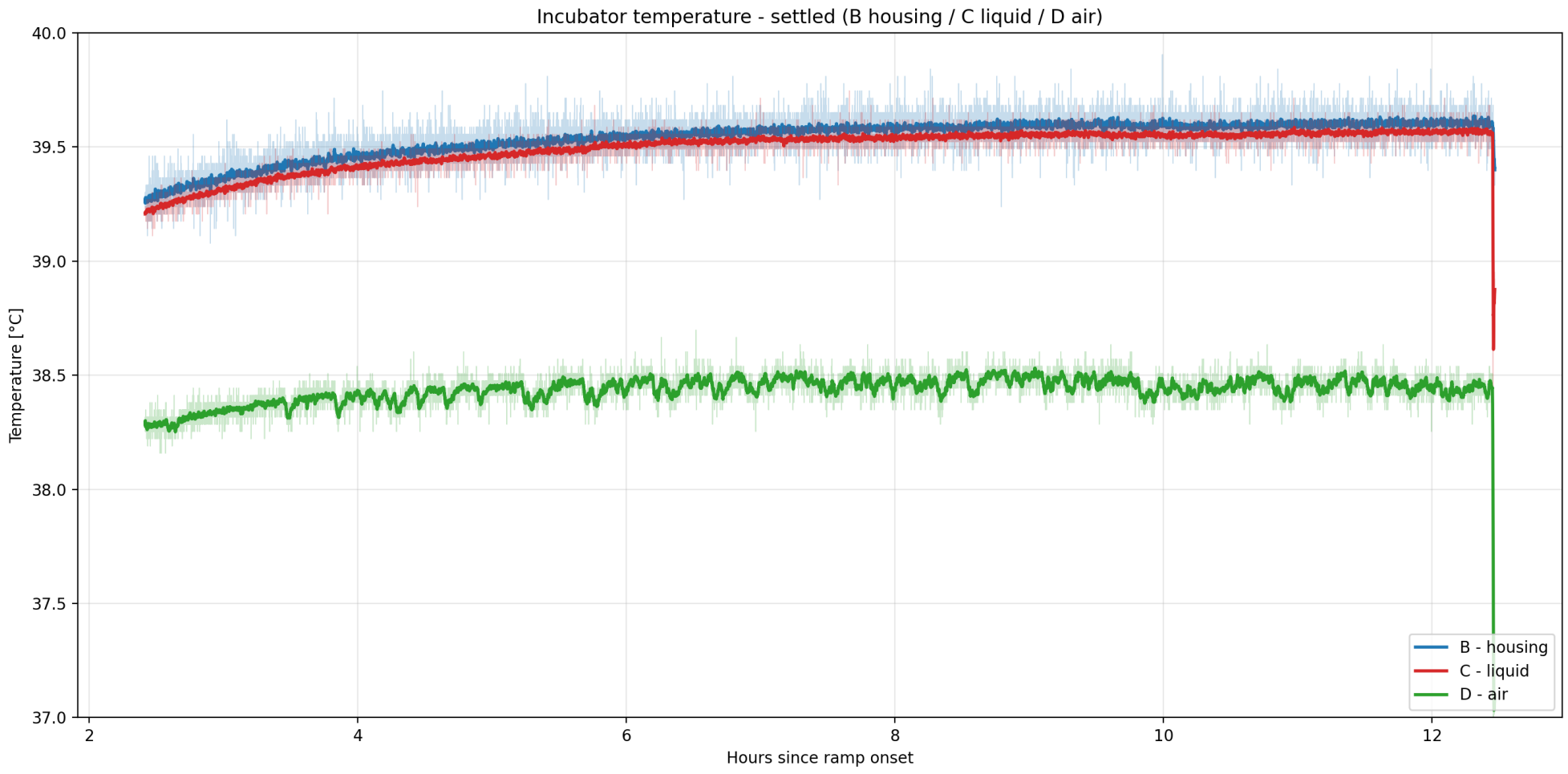

Fig. S16: **Settled temperature distribution of the conventional recording setup.** The same three sensors after the ramp has settled (thin lines, raw samples; thick lines, moving average). The housing and the liquid agree to within approximately 0.05 °C and drift together, while the air above the lid is approximately 1.1 °C cooler. The medium is thus the warmest element and the lid the coolest, the gradient that drives evaporation from the medium and condensation onto the lid, and the one that the temperature control of inkudock inverts (Fig. S17).

in the centre and a higher temperature outside. For all further experiments, the HD-MEA surface was controlled to this value, and the temperature sensor in the liquid was omitted to reduce the risk of contamination.

B.4 Evaporation in incubator with membrane lid

Fig. S18 shows evaporation from HD-MEAs in a Petri dish and with a commercial membrane lid in commercial incubators to provide a reference for expected evaporation from HD-MEAs following state of the art protocols.

B.5 Evaporation from the water compartment

Evaporation from the water compartment itself was determined by weighing the water compartment lid during the runs recorded for Fig. 1f (Fig. S19). A linear fit gives 1.11 µL h^{-1}, close to the evaporation of an MEA well under a membrane lid in commercial incubators (Fig. S18). At this rate, the 2 mL compartment loses about 0.27 mL (13 %) in ten days. Since the vapour barrier relies on a continuous water layer between the two membranes, the compartment should be refilled well before depletion; in all experiments reported here it was refilled at least every 10 days. The refill of the long-term recording is visible in the environmental data (Fig. S28) but not in the activity (Fig. S30).

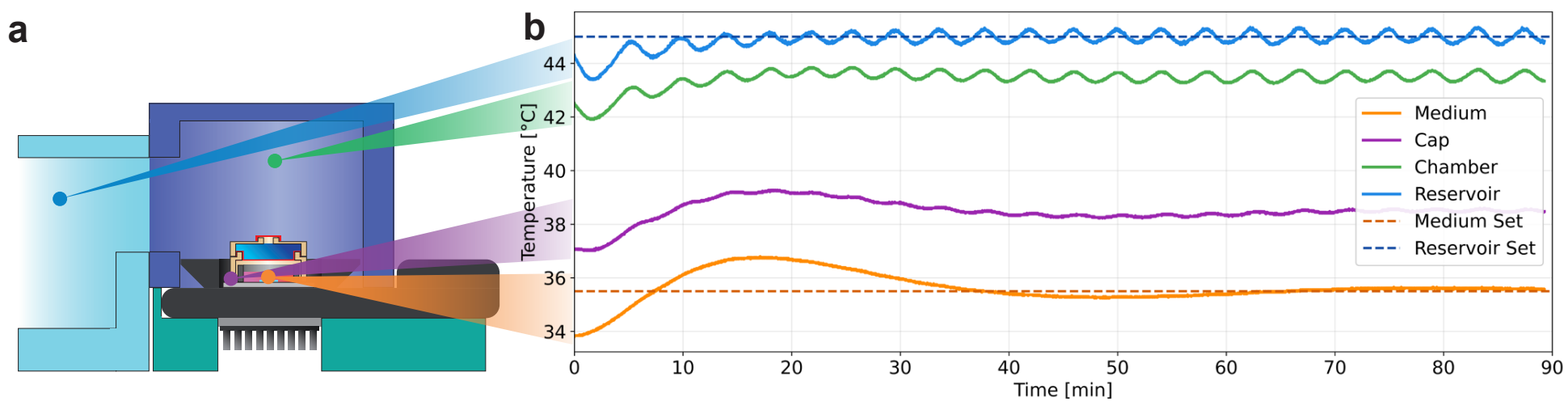

Fig. S17: Temperature control calibration of the incubation setup. (i) Measurement locations of the temperature sensors. An independent loop controls the reservoir temperature and is set to 45 ° C (blue). A second sensor measures the temperature inside the chamber (green). In order to determine the set temperature for the second control loop with the temperature sensor on the chip (purple), a sensor is placed inside the liquid with a set temperature of 35.5 ° C (orange). In steady state, the chip temperature is 38.5 ° C, which is further used as the set temperature for the chip sensor (purple).

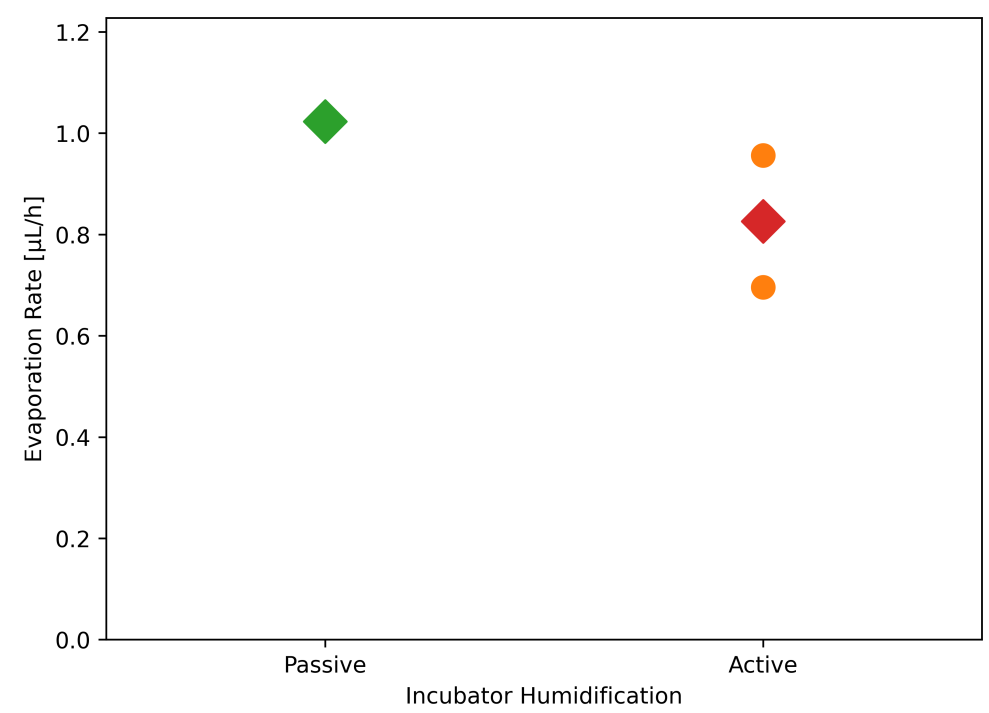

Fig. S18: **State-of-the-art evaporation in incubator.** Evaporation was measured over 48 h from 3 HD-MEAs in commercial incubators. One was placed in an incubator with passive humidification, and two were placed in incubators with active humidification. For the latter, the individual measurements are presented as dots and the mean is marked with a diamond. All MEAs were placed with 1 mL of water and a commercial membrane lid was mounted. MEAs were weighed before and after 48 h to determine the volume of evaporated liquid.

B.6 Gas exchange and filling volume of the water compartment

The CO_2 step response of Fig. 1g was repeated with the sensor dummy chip (Supplementary Note A.3.1) for an empty, a half-filled (1 mL) and a fully filled (2 mL) water compartment (Fig. S20). Equilibration becomes faster as the filling volume decreases, so that evaporation from the compartment does not impair gas exchange; the full compartment at the start of an experiment is the limiting condition.

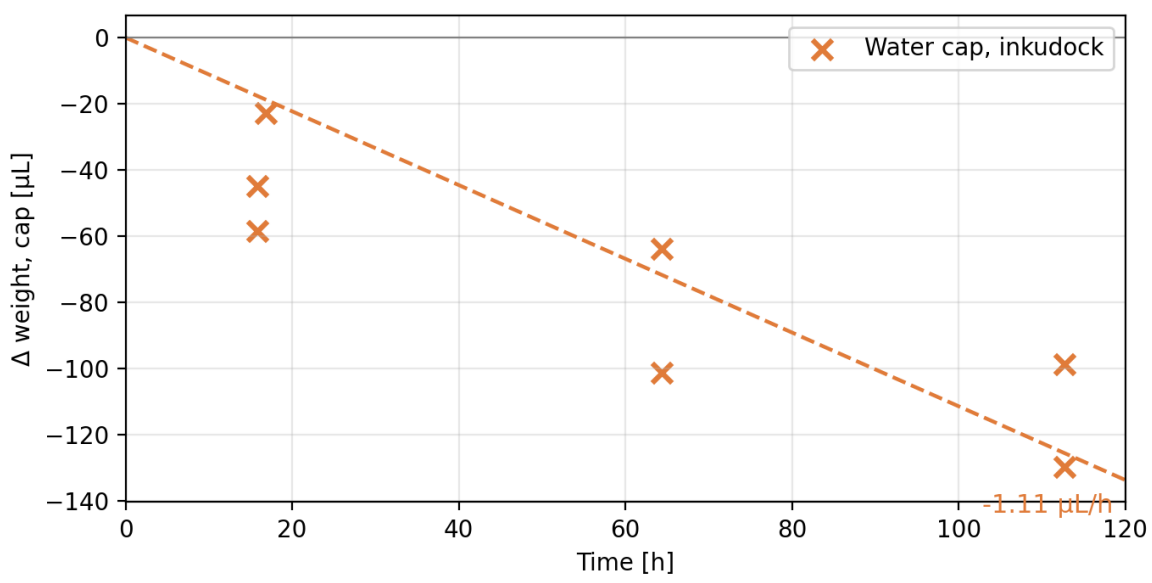

Fig. S19: **Evaporation under the water compartment lid in inkudock.** The difference in lid weight that was recorded for Fig. 1f is shown over time, with the linear fit used to determine the rate.

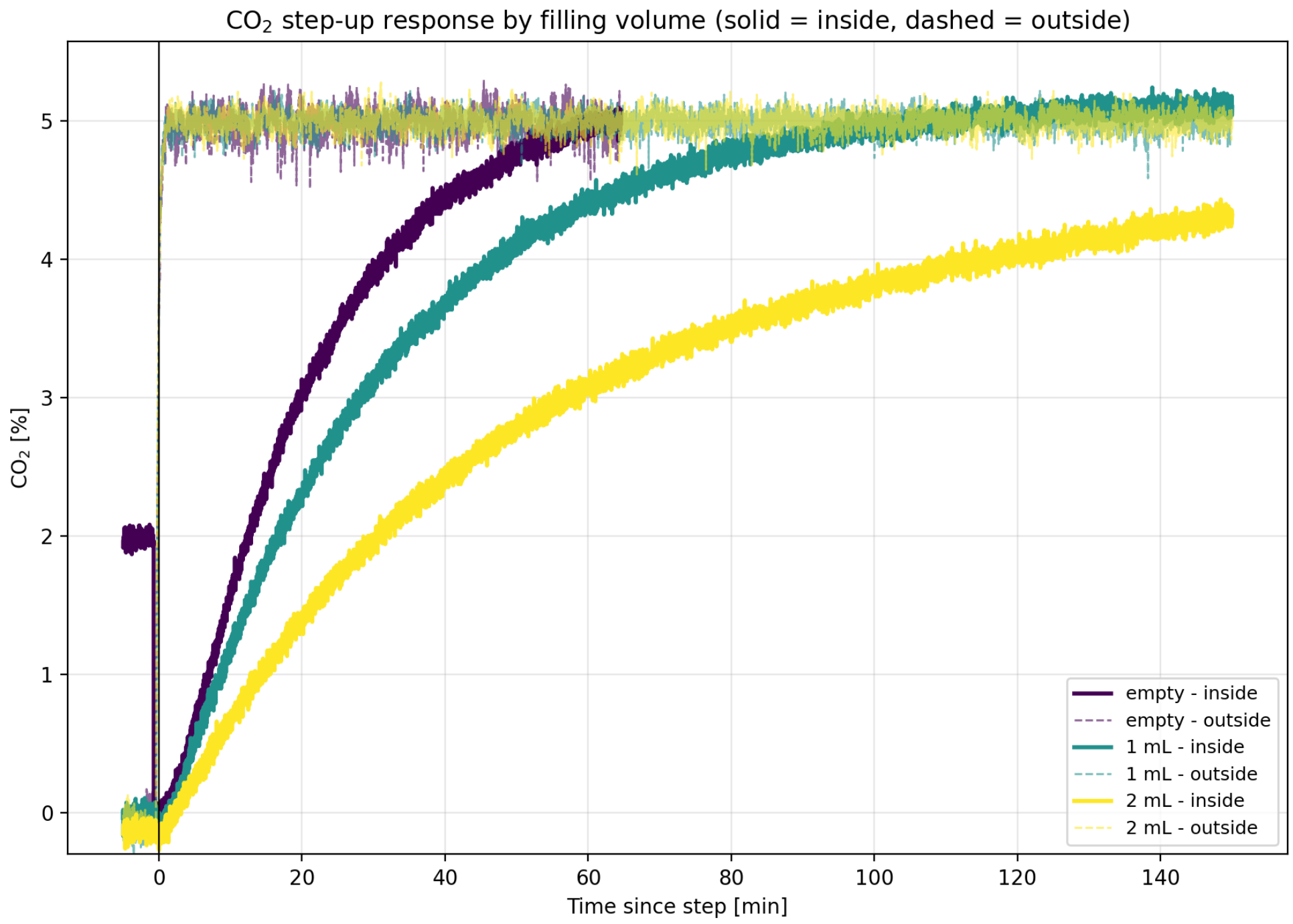

Fig. S20: **Gas exchange through the water compartment, resolved by filling volume.** CO_2 concentration inside the sealed culture chamber (solid) and in the surrounding incubator atmosphere (dashed) following a step change of the external concentration at $t = 0$, measured on the sensor dummy chip of Fig. S12, for an empty compartment (dark), 1 mL (teal) and the nominal 2 mL filling (yellow). The external concentration follows the step within seconds in all three cases. Internally, the approach to the new equilibrium slows as the water column deepens, while the same final concentration is reached in every condition.

C Medium exchange experiment

C.1 Baseline comparability of the two groups

To test whether the cultures of the two lid conditions in Fig. 2 were comparable before the medium exchange, the mean firing rate over the 6 h preceding each exchange (Fig. S21) and the standard deviation of the firing rate across the four regions of a network (Fig. S22) were compared between groups. Neither differs significantly.

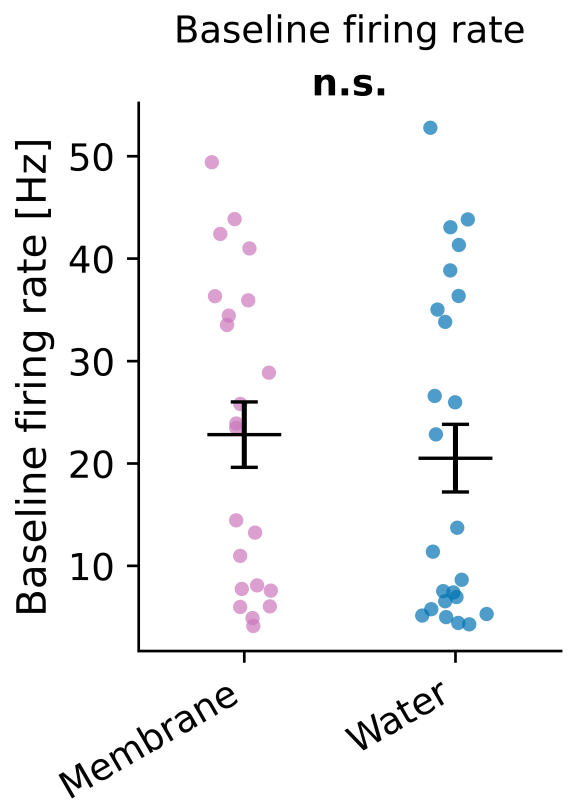

Fig. S21: **Baseline firing rates of the membrane and water compartment groups.** Mean firing rate of each culture over the 6 h preceding the medium exchange, for the conventional membrane lid and the water compartment lid. The difference between the membrane and water compartment groups is not significant (linear mixed model, $n = 22$ membrane, $n = 24$ water cultures, $p = 0.62$). Bars are mean $\pm$ s.e.m.; each point is one culture.

C.2 Recovery after medium exchange

Firing rate was normalised to each culture's own mean over the 6 h preceding the exchange and averaged in 1 h bins from 6 h before to 6 h after the exchange (Fig. S23). Under the water compartment lid the relative rate stays close to baseline, rising slightly to 1.05 in the first two hours and returning to 1.01 by +6 h. Under the conventional membrane lid it falls to 0.77 in the first hour and reaches only 0.87 six hours later, so that it does not return to 90 % of baseline within the observation window.

C.3 Transfer entropy threshold and edge stability

Stable edges in Fig. 2f,g are defined by a threshold of TE $\geq 1 \times 10^{-4}$, applied on top of the surrogate-based significance test, and by their presence at a consistent lag in all three windows before the exchange. Fig. S24 shows the distribution of significant TE values and the fraction retained by the threshold, and Fig. S25 the edge count

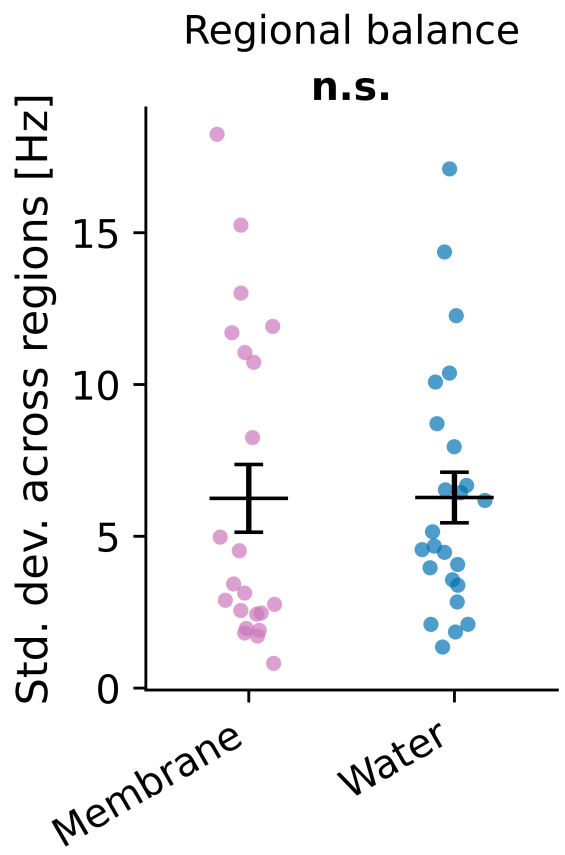

Fig. S22: **Regional balance of baseline activity in the two groups.** Standard deviation of the baseline firing rate across the four regions of each culture, as a measure of how evenly the network is engaged. The difference between the membrane and water compartment groups is not significant (linear mixed model, $n = 22$ membrane, $n = 24$ water cultures, $p = 0.90$). Bars are mean $\pm$ s.e.m.; each point is one culture.

changes between all pairs of windows. Fig. S26 repeats the comparison of Fig. 2f with the unchanged pipeline for thresholds between 1×10^{-5} and 1×10^{-3}, tested with a linear mixed model on the edge-level fold changes with network as random intercept, since edges of the same network are not independent.

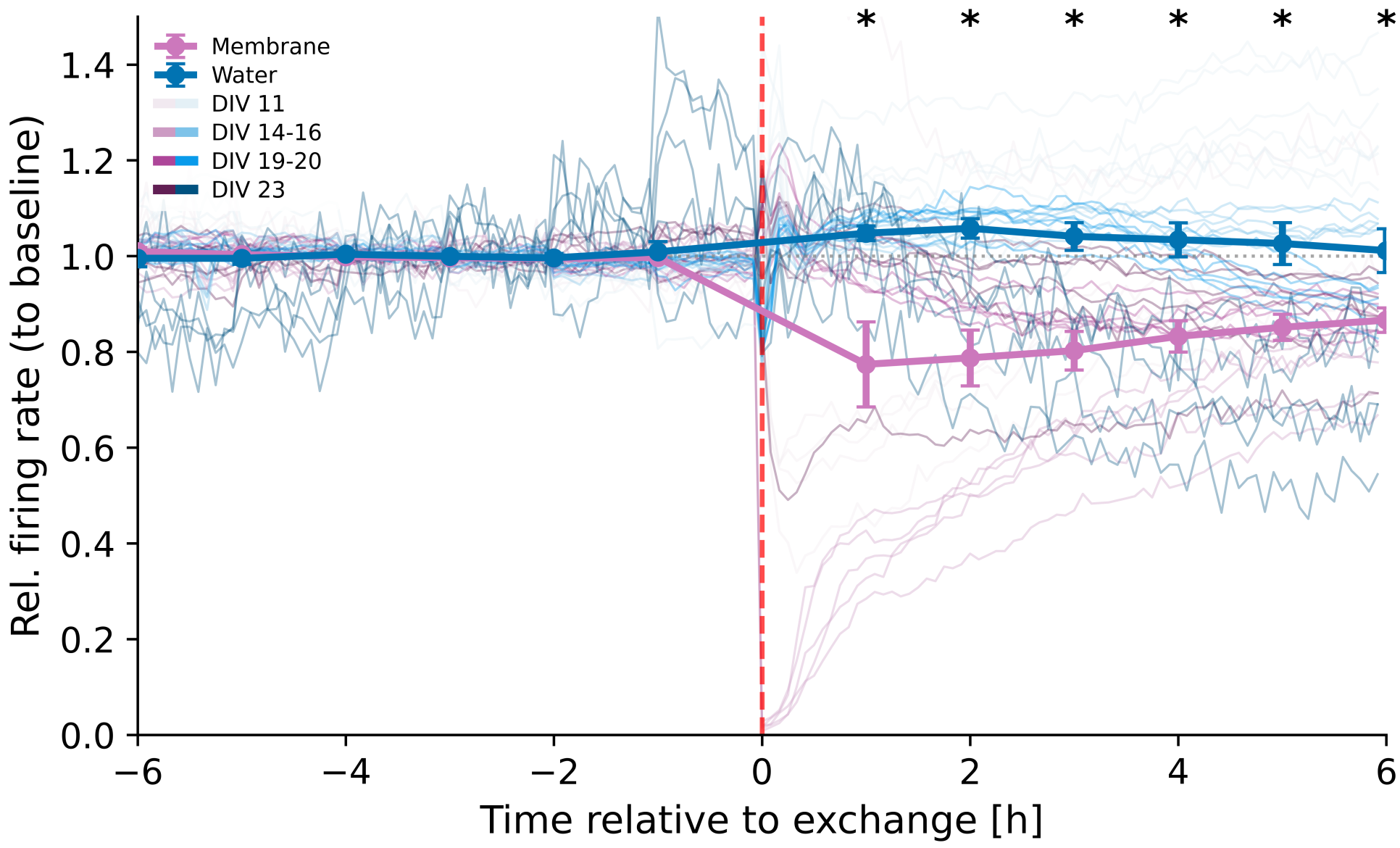

Fig. S23: **Firing rate around medium exchange in the two groups.** Firing rate relative to each culture's own mean over the 6 h preceding the exchange (dotted line, 1.0), for the conventional membrane lid (pink, $n = 22$ cultures) and the water compartment lid (blue, $n = 24$ cultures); faint lines are individual cultures, coloured by DIV at the time of the exchange (light = DIV 11, dark = DIV 23). The red dashed line marks the exchange. Bold points are mean ± s.e.m. over cultures. The bracket reports a linear mixed model (random intercept per network) comparing, for each culture, its mean absolute deviation from baseline over the 0–6 h post-exchange window — the same approach used to compare slopes in Fig. 2d — and shows that the membrane group deviates from its own baseline significantly more than the water compartment group overall ($p = 0.046$). The membrane group falls to 0.77 at +1 h and has reached only 0.87 at +6 h, not returning to 90% of baseline within the observation window, whereas the water compartment group stays close to baseline throughout.

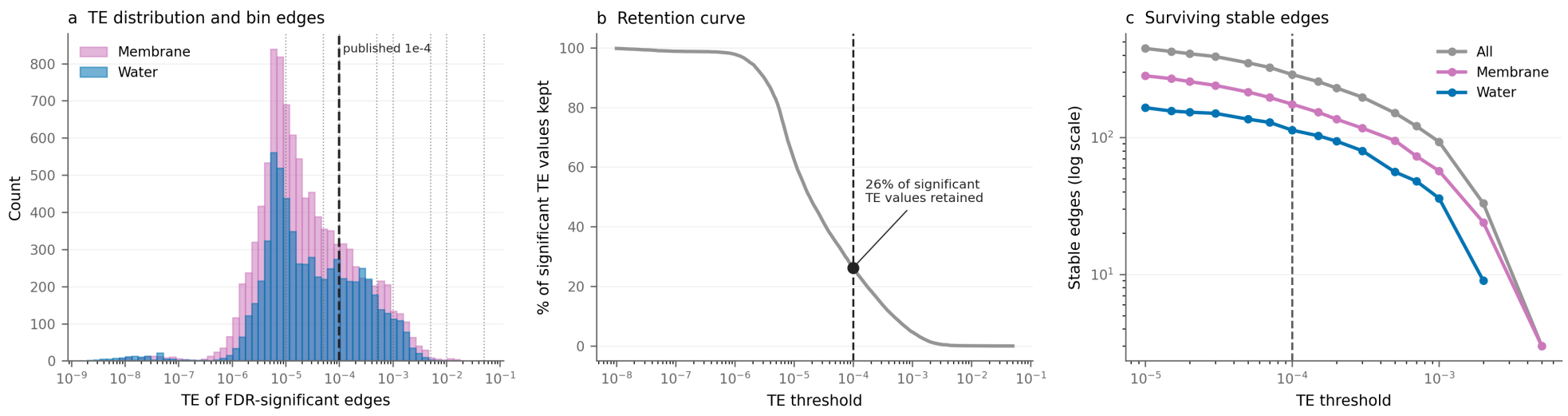

Fig. S24: **Distribution of transfer-entropy values and choice of the edge threshold.** Medium exchange experiment of Fig. 2, membrane lid (pink) and water compartment lid (blue). **a**, Distribution of the TE values of all edges that passed the surrogate test (FDR-adjusted $p < 0.05$), over all lags and windows. The dashed line marks the threshold used in the main text (1×10^{-4}), the dotted lines the thresholds tested in Fig. S26. **b**, Fraction of significant TE values retained as a function of the threshold. At 1×10^{-4}, 26 % of the significant values are retained. **c**, Number of stable edges (present at the same lag in all three pre-exchange windows) surviving as a function of the threshold, for all networks and per condition (logarithmic scale).

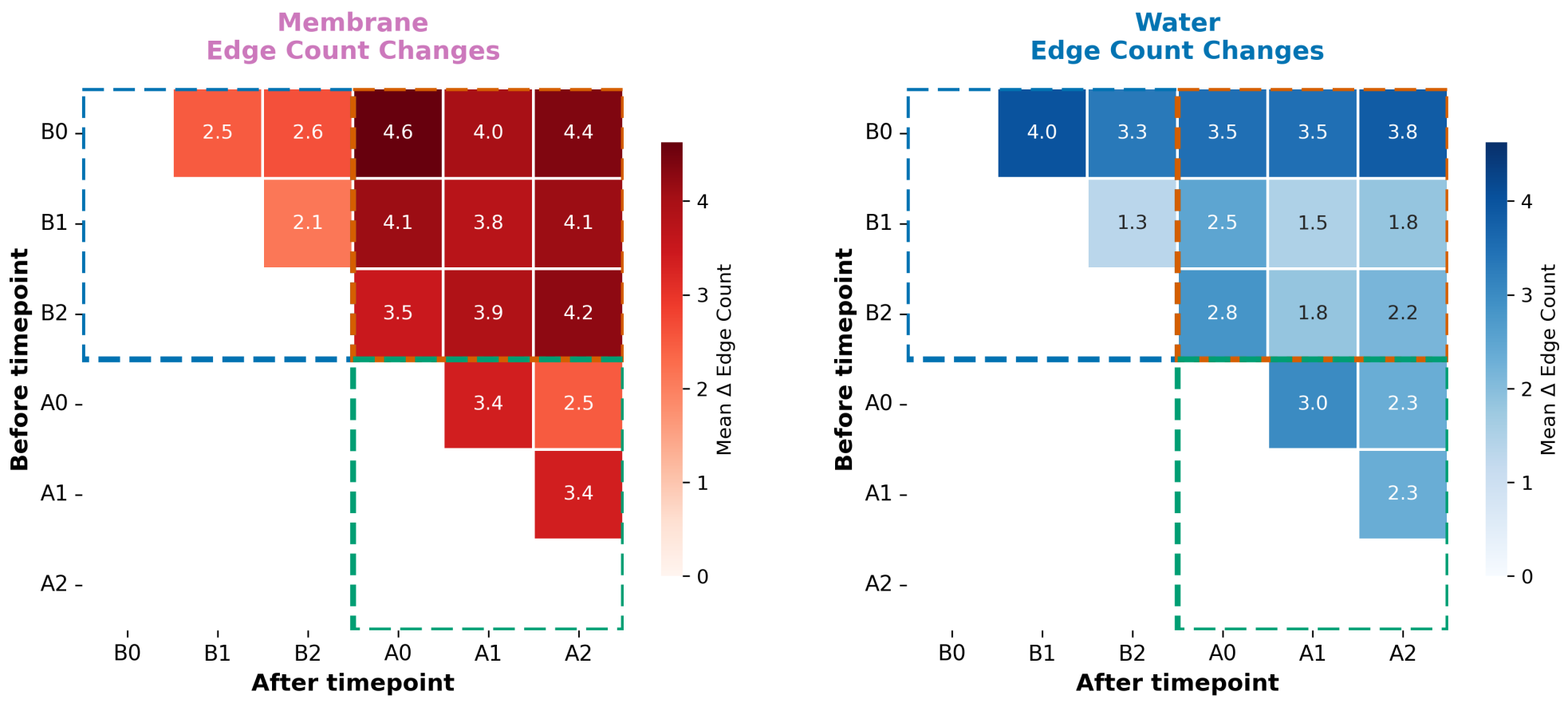

Fig. S25: Edge count changes across all timepoint pairs. Mean edge count changes (sum of absolute differences per source-target connection, normalised TE $\geq 1 \times 10^{-4}$) for all transitions. Networks with weakly connected component $\geq$3 nodes included. Boxes highlight within-period (blue, green) versus across-exchange (orange) transitions for membrane and water conditions.

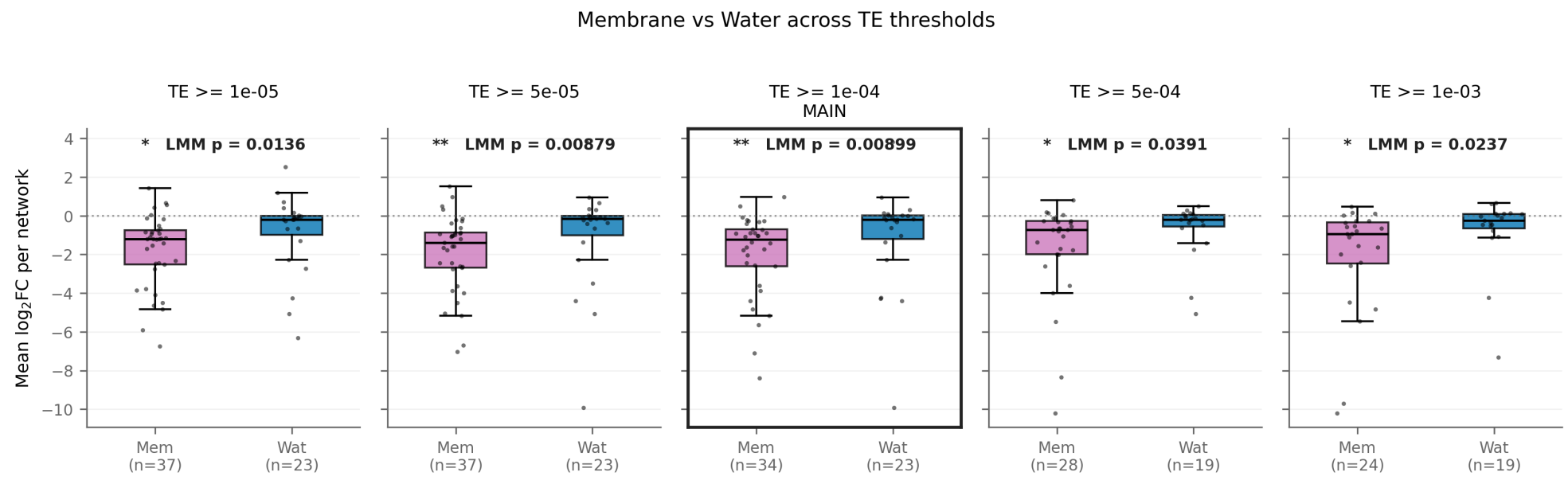

Fig. S26: **Edge persistence across medium exchange for different TE thresholds.** Mean $\log_2$ fold change of the transfer entropy of stable edges per network across the medium exchange, as in Fig. 2f, recomputed with the unchanged pipeline for edge thresholds from 1×10^{-5} to 1×10^{-3}; the framed panel is the threshold of the main manuscript. n gives the number of network–exchange observations that retain at least one stable edge. p values are from a linear mixed model on the edge-level fold changes with network as random intercept, which accounts for the dependence between edges of the same network. The membrane lid shows a significantly stronger reduction at every threshold shown (p between 0.009 and 0.039).

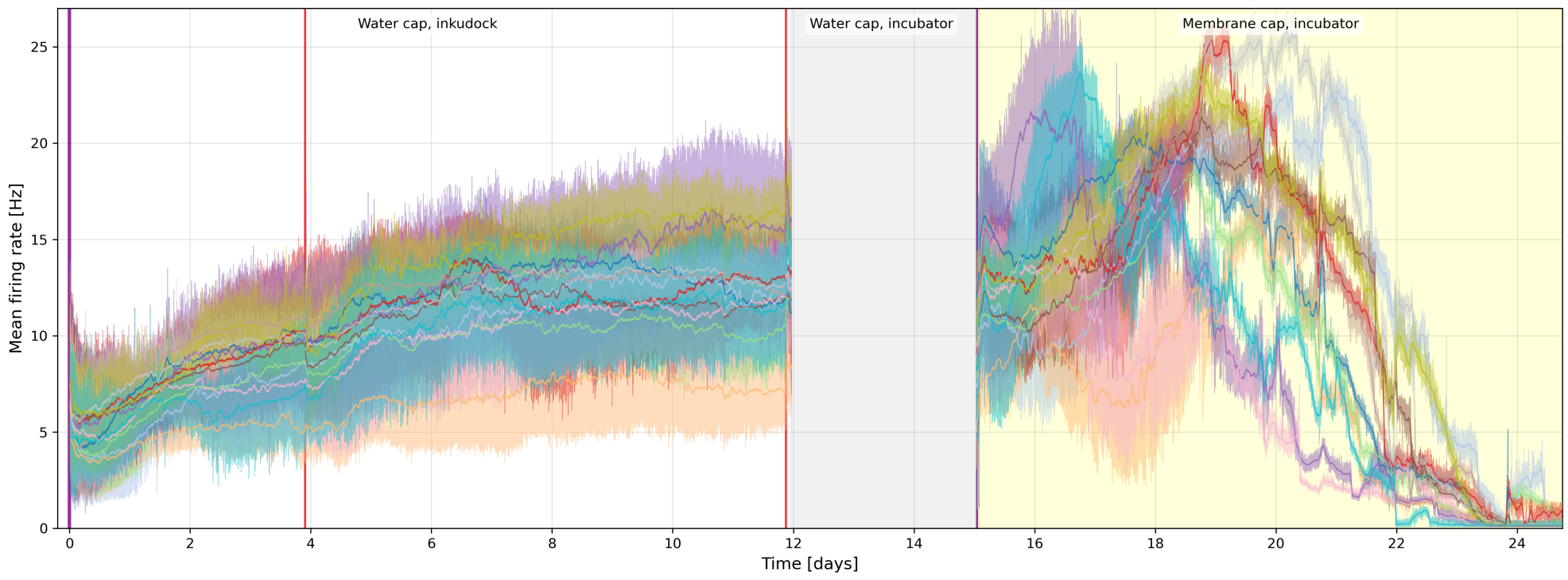

Fig. S27: Firing rate of a cell culture during medium exchange and different recording conditions. Electrodes were selected in the microchannels. Activity is processed by network, binned into 10 s and a 3-point median is applied (faint lines) to avoid a drop due to overlap of bins with the data saving breaks. The thin lines show an average across 60 min, excluding the bins during data saving.

D Long-term recording

D.1 Culture moved between conditions

A cell culture (chip D) with a microstructure of 12 networks, each with 2 seeding wells connected via 10 parallel microchannels, was recorded starting at 49 DIV. The mean firing rate per network is shown in Fig. S27. The culture was initially recorded with the water cap in inkudock following a full medium exchange. At day 4 and day 12, half of the culture medium was exchanged. After the second half exchange (day 12), the culture was moved to a standard incubator without recording. At day 15, a brief recording was taken, followed by a full medium exchange. The culture was then recorded for 2 h in the inkudock. The water cap was then exchanged with a commercial membrane cap, and it was transferred to the incubator, where recording continued until firing ceased. Notably, firing rates remained remarkably stable during the inkudock recordings (days 0–12 and day 15), with gradual increases over the first 12 days. In contrast, upon transfer to the standard incubator (day 15 onwards), all networks exhibited rapid perturbations within hours followed by an increase in firing rate between 17-19 days and a decline afterwards. The spike-time-triggered raster plots and the pattern repertoire of network 9 of this culture are shown in Figs. S50 to S52.

D.2 Environmental data during long-term recording

Temperature settings and CO_2 concentration during the long-term recording in inkudock are shown in Fig. S28. The medium exchange after 22 days is visible as a fluctuation in the temperature and the CO_2 is increased to 10 % for 30 min after restart. After 10 days, a refill of the water compartment is performed, leading to a dip and small overshoot in the chip temperature.

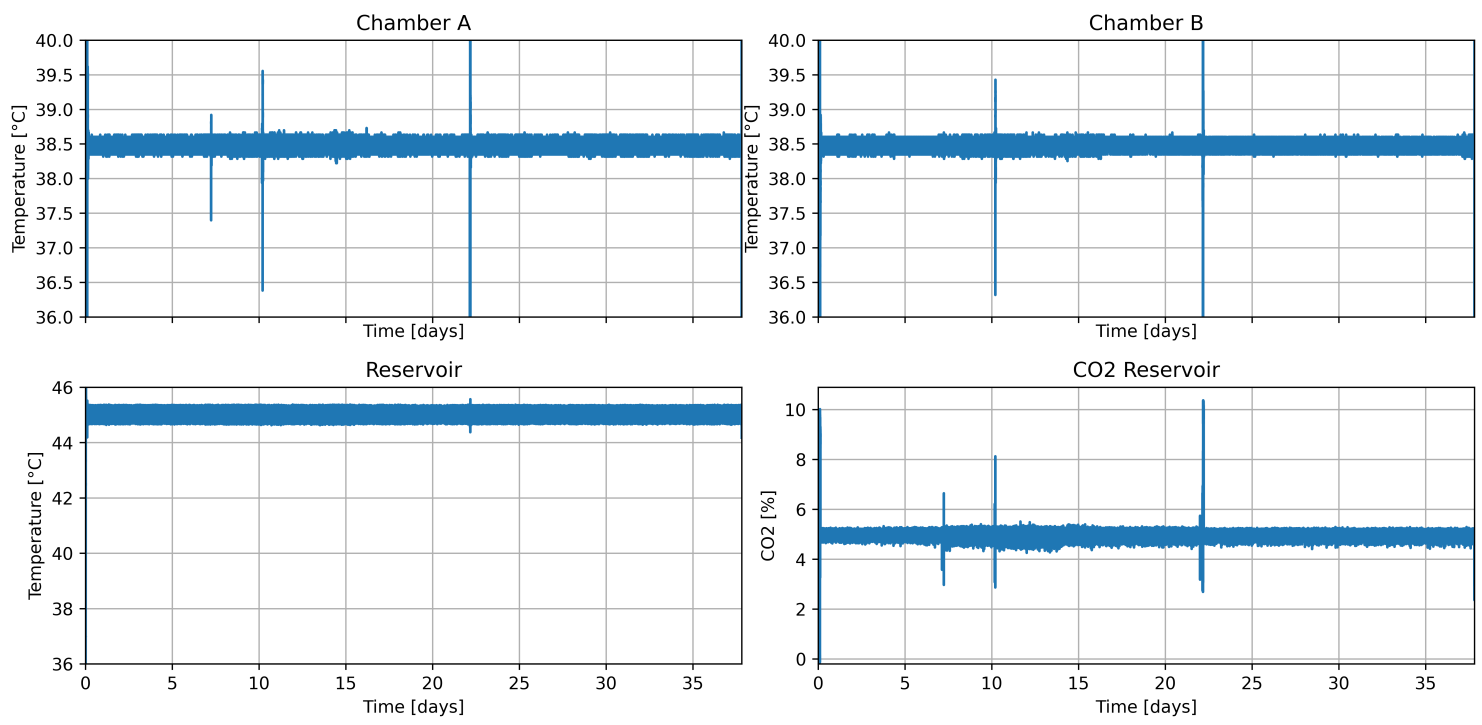

Fig. S28: Culturing conditions during the long-term recording. The temperature in the chambers is set to 38.5 °C, with interrupts in Chamber A during opening of the chamber lid on day 7, the water compartment refill on day 10 and the medium exchange on day 22. The reservoir temperature is unaffected and stable at 45 °C. The CO_2 concentration is set to 5 % with small deviations at chamber opening and the 10% settings for 30 min at start and after medium exchange.

D.3 Spike detection and amplitude

Spikes were detected online by the MaxWell acquisition software with a negative amplitude threshold at a fixed multiple of the per-channel noise standard deviation (5 standard deviations for the long-term recordings), and only spike times and amplitudes were stored. Fig. S29 shows the amplitude distribution of network 2 of chip A over the whole recording, Fig. S30 the per-electrode mean and standard deviation over time, and Fig. S31 the daily distributions. Outside the quiet interval the amplitude distribution is stable; during the quiet interval it shifts to lower values while the number of spikes falls by more than an order of magnitude, and it recovers once activity re-emerges.

D.4 Burst analysis

Bursts and network bursts were detected and the burstiness index was computed following Wagenaar *et al.* Wagenaar et al. (2006), as detailed in the captions of Figs. S32 and S33. For network 2 of chip A, the index stays below about 0.2 up to DIV 23, rises to about 0.4 at DIV 25 and remains elevated until the quiet interval, and settles near 0.08 after the re-emergence of activity.

D.5 Medium comparison

Culture medium was sampled from MEA cultures with the water compartment cap after 14 days without medium exchange and from cultures after 3 days in the incubator with a membrane cap. Both cultures were seeded at a density of 150 k cells.

Glucose concentration was measured using a commercial blood testing glucometer (CONTOUR NEXT 20438293, Ascensia Diabetes Care, Parsippany, NJ, USA).

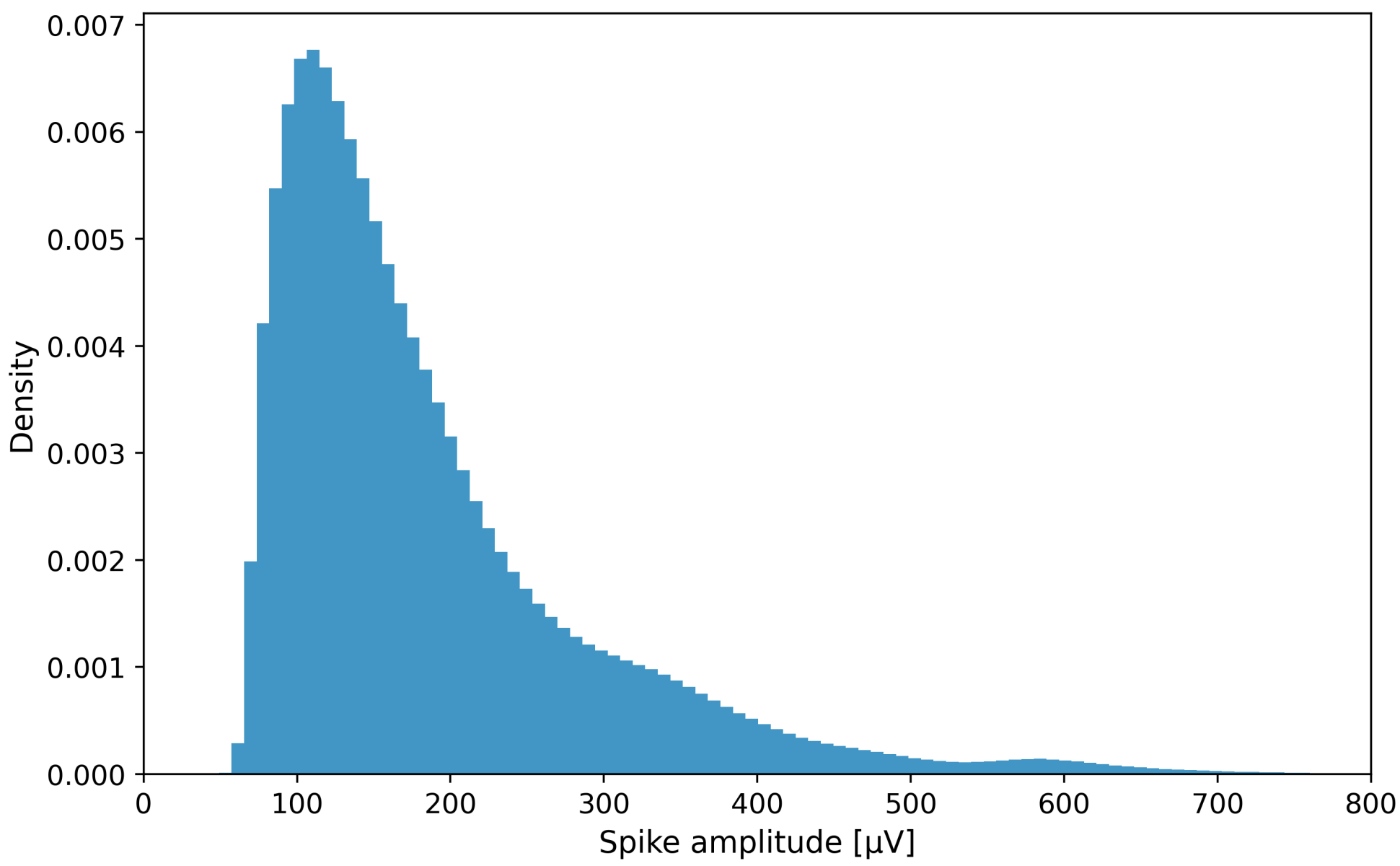

Fig. S29: **Spike amplitude distribution over the long-term recording.** Network 2, chip A; all detected events of the recording shown in Fig. 3 of the main text. The distribution is unimodal with a long tail; the sharp low-amplitude edge is the per-channel detection threshold.

Medium was diluted 1:1 with PBS to fit within the glucometer's measurement range. Linear regression through all data points with a fixed intercept at 100% (fresh medium) yielded a glucose consumption rate of -1.36 %/day ($R^2 = 0.679$). After 14 days, glucose levels remained at approximately 80 % of the initial concentration. Given the high glucose concentration in Neurobasal medium (25 mM), glucose depletion is unlikely to affect culture viability.

The NanoDrop One Microvolume UV-Vis Spectrophotometer (ThermoFisher) was used to characterise the protein content in the different medium conditions. The measurements are shown in Fig. S35 and Fig. S36

Protein electrophoresis was performed using the NuPAGE™ Bis-Tris gel system (NP034B, Invitrogen) with the same media, and gels were stained with SimplyBlue™ SafeStain (LC6060, Invitrogen). Protein size was estimated using the PageRuler™ Prestained Protein Ladder, 10 to 180 kDa (26616, Thermofisher) as calibration. Protein loads of 10 and 20 µg were analysed in Fig. S37.

D.6 Additional network recorded for 14 days

The HD-MEA (chip C) was prepared as described in Methods. On 25 DIV, a full medium exchange was performed, the water compartment lid was mounted, and a continuous recording was started. The pattern analysis of this network is given in Supplementary Note E.6.

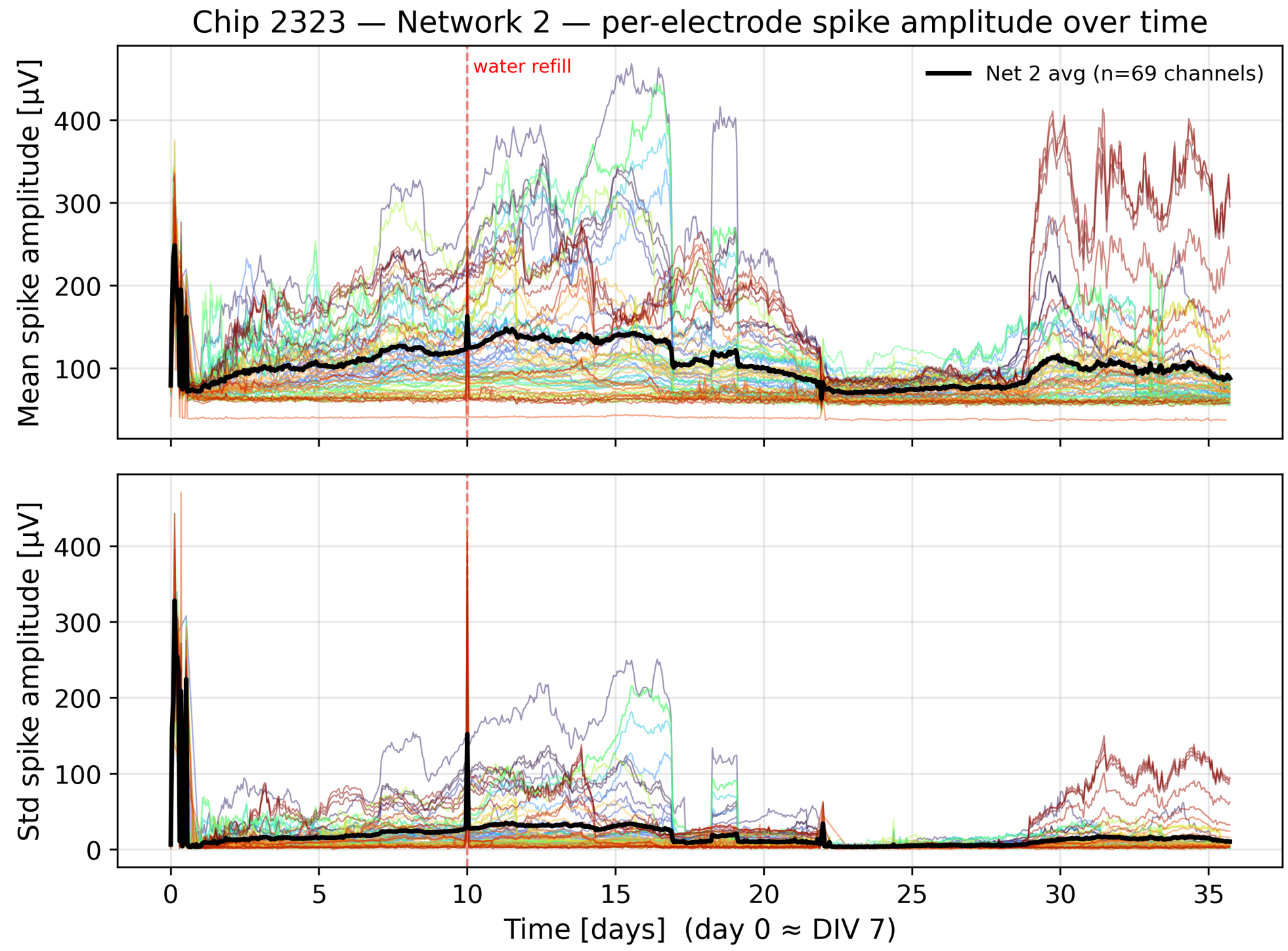

Fig. S30: **Spike amplitude over the recording.** Network 2, chip A; per-electrode mean (top) and standard deviation (bottom) of the spike amplitude against recording time (day 0 ≈ DIV 7), one coloured line per electrode, black line the array average over $n = 69$ channels. The red dashed line marks the water compartment refill on day 10. There is no progressive drift of the amplitude towards or away from the detection threshold; the mean decreases during the quiet interval and recovers afterwards (see Fig. S31), and the refill produces a transient in the standard deviation without a step in the mean.

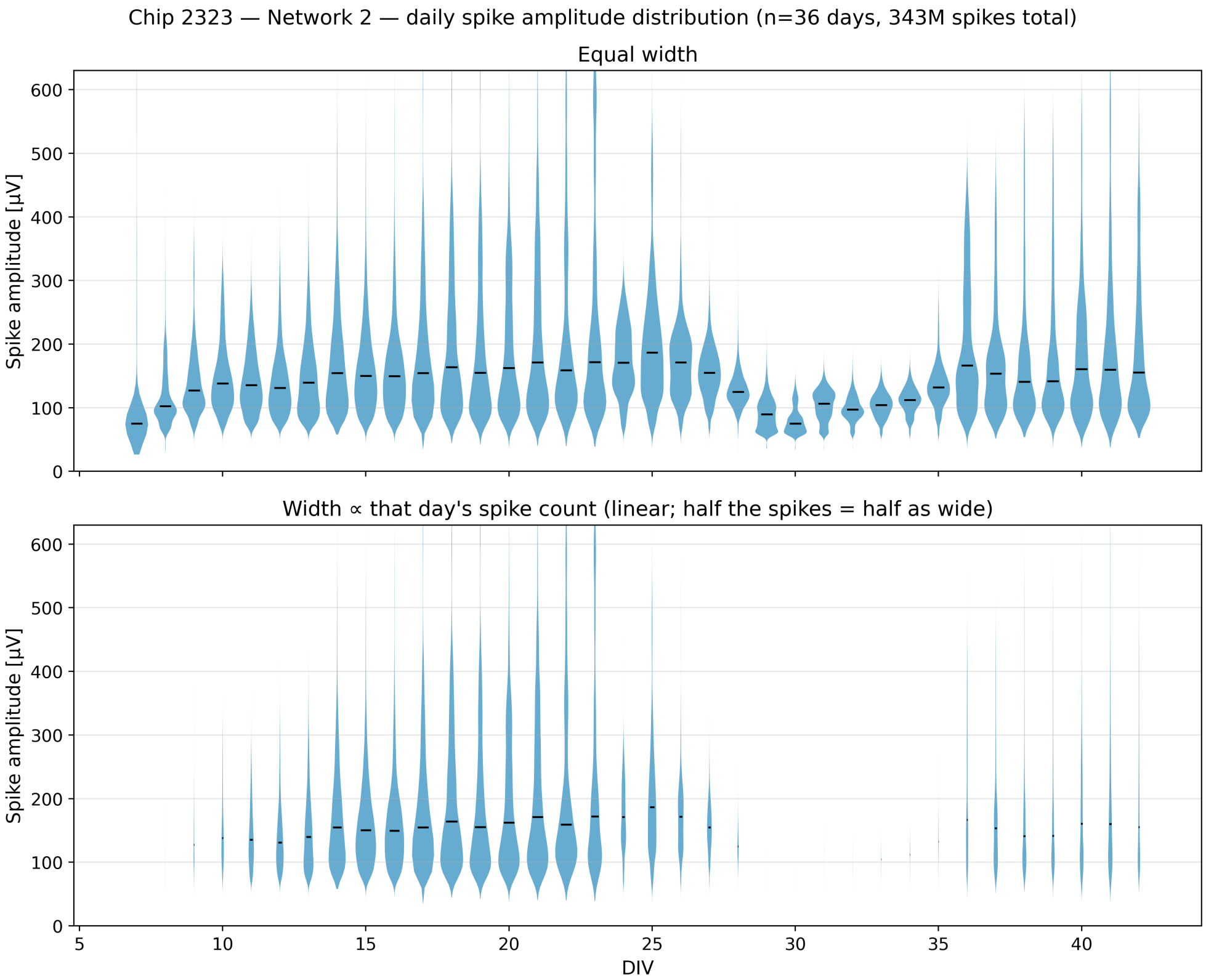

Fig. S31: **Daily spike amplitude distributions.** Network 2, chip A; daily spike amplitude distribution over 36 days (343 M spikes). Top, all days drawn at equal width: the distribution and its median are stable over most of the recording, but shift to lower amplitudes and lose the high-amplitude tail during the quiet interval (DIV 28 to 34), before recovering once activity re-emerges. Bottom, the same distributions with the width of each violin proportional to that day's spike count: during the quiet interval the number of events falls by more than an order of magnitude.

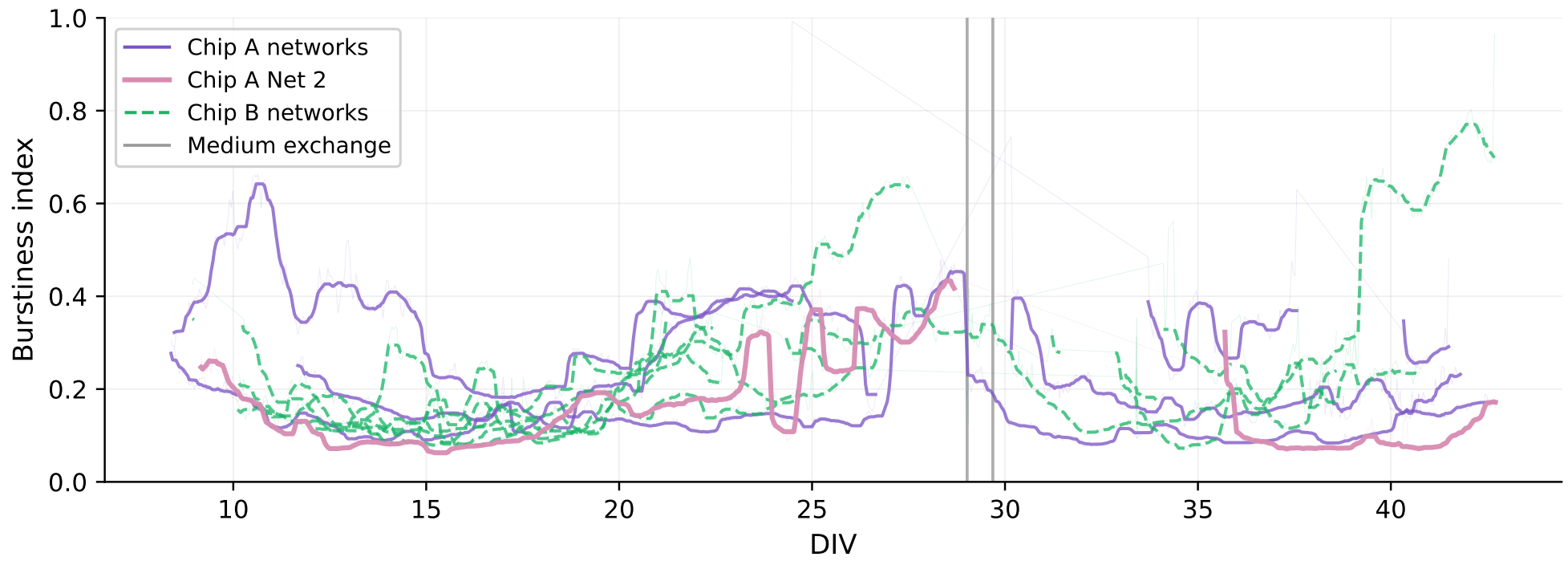

Fig. S32: **Burstiness index over development.** Burstiness index of each spatial network over the long-term recording shown in Fig. 3 of the main text, one line per network, with a 12 h rolling median drawn thick over the individual ~1 h recordings (faint). The index was computed per recording and per network after Wagenaar *et al.* Wagenaar et al. (2006) as BI = $(f_{15} - 0.15)/0.85$, where f_{15} is the fraction of all spikes falling in the 15 % most active non-overlapping 1 s windows of the population spike count, so that 0 corresponds to spikes spread uniformly in time and 1 to all spikes confined to the most active 15 % of the recording. Recordings below 0.1 Hz per electrode are omitted, since the index is not meaningful at that sparsity, which leaves the gaps in the traces. Grey vertical lines mark the recording interruptions accompanying the half-medium exchange.

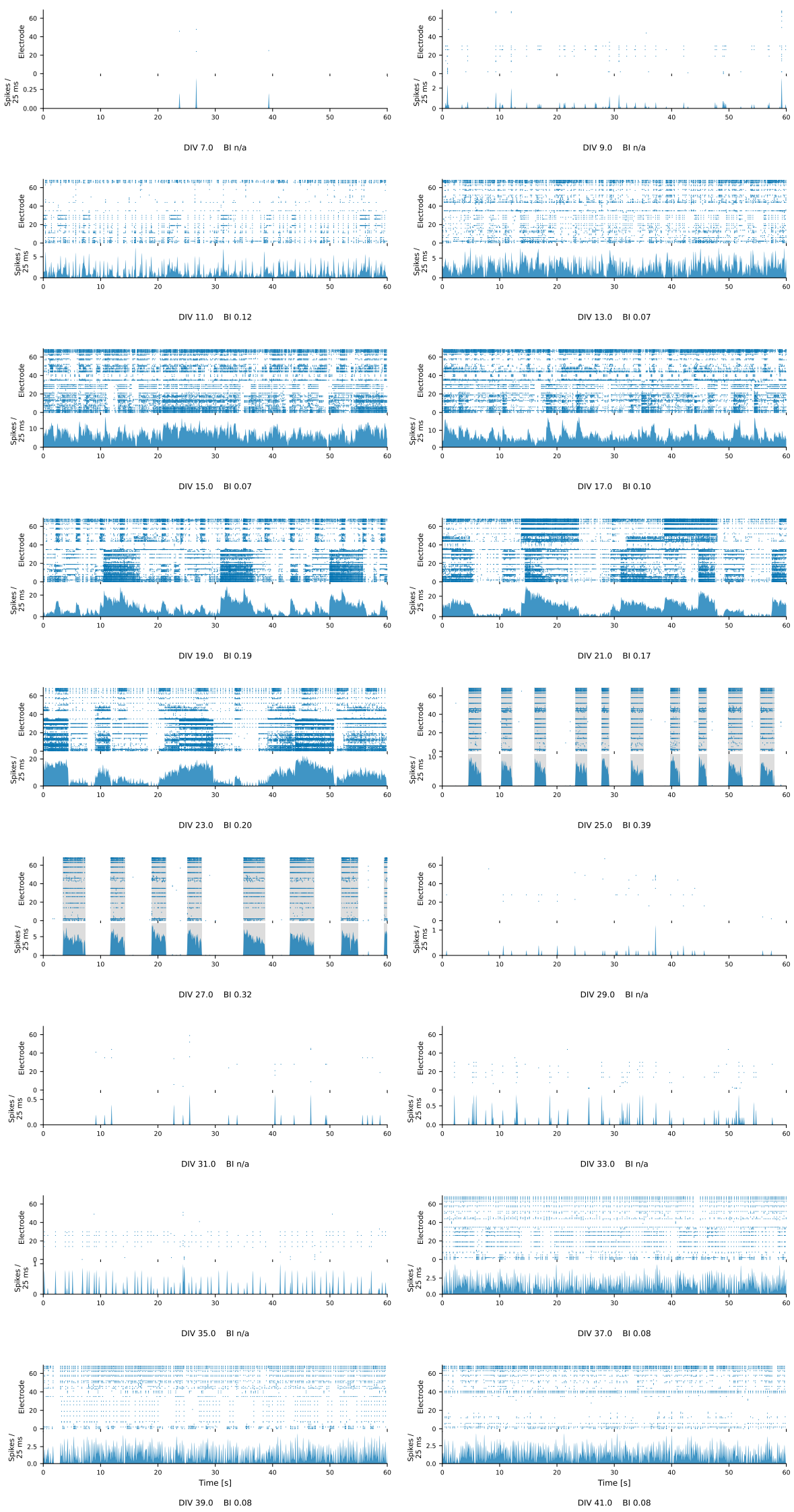

Fig. S33: **Network bursting across development.** Sixty-second excerpt from one recording every two days for network 2 on chip A, showing the spike raster (top of each panel, one row per electrode) and below it the population firing rate, summed over the 69 electrodes of the network in 25 ms bins and smoothed with a Gaussian kernel ($\sigma = 2$ bins). Detected network bursts are shaded grey and the burstiness index of the recording is given in the panel title.

Fig. S33: (continued) Each 3,600 s recording was analysed independently and classified as bursting only where its burstiness index, computed over non-overlapping 1 s windows, exceeded 0.25 Wagenaar et al. (2006). Burst onset and offset were then thresholded at 25 % and 10 % of the difference between that recording's median and 99th-percentile population rate, with at least 30 % of the electrodes required to participate; bursts separated by less than 200 ms were merged and bursts shorter than 50 ms discarded. Recordings below 0.1 Hz per electrode were left unassessed (BI n/a). The progression from sparse activity, through dense irregular firing, to pronounced network bursting and the subsequent quiet interval is visible in the unprocessed data.

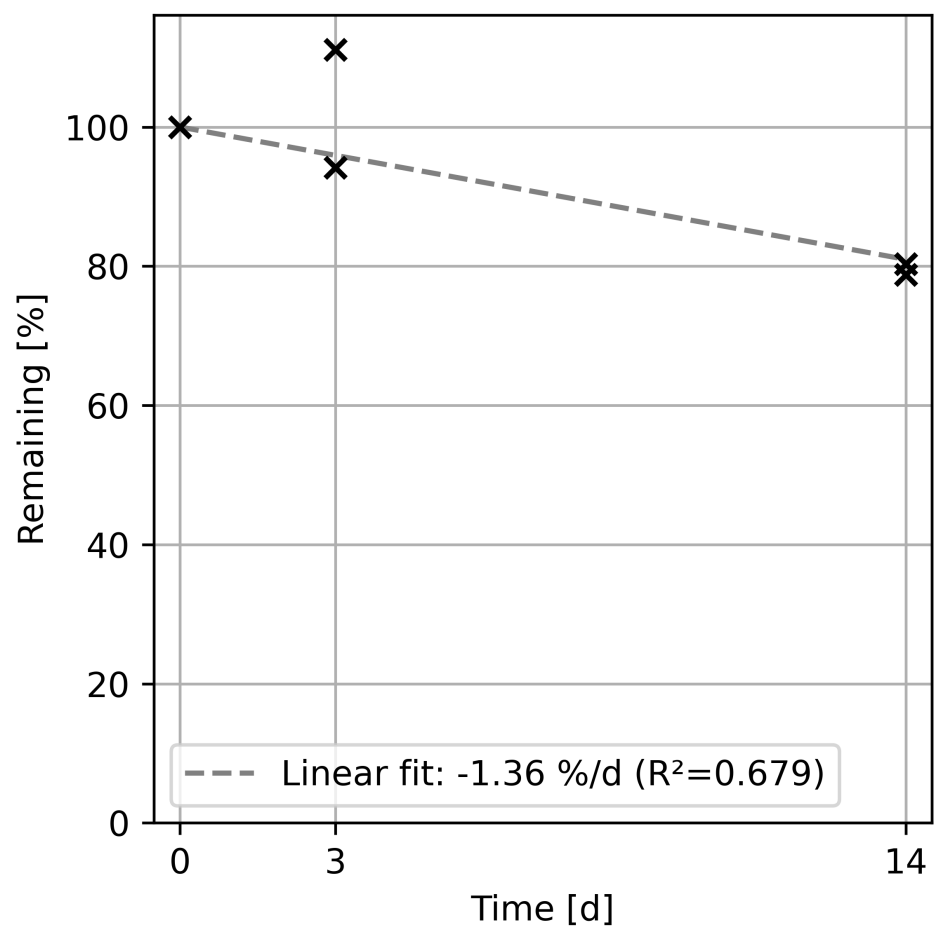

Fig. S34: Glucose concentration remaining in culture medium over time. Fresh medium (day 0) serves as 100% reference. Individual measurements from day 3 and day 14 are shown with crosses. Dashed line represents linear fit forced through 100% at day 0.

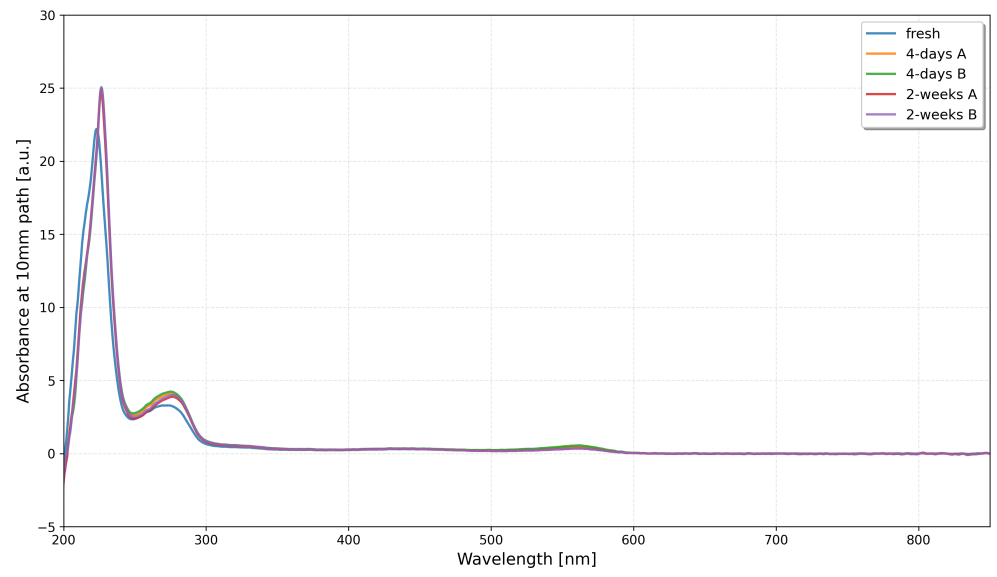

Fig. S35: Nanodrop analysis of samples.

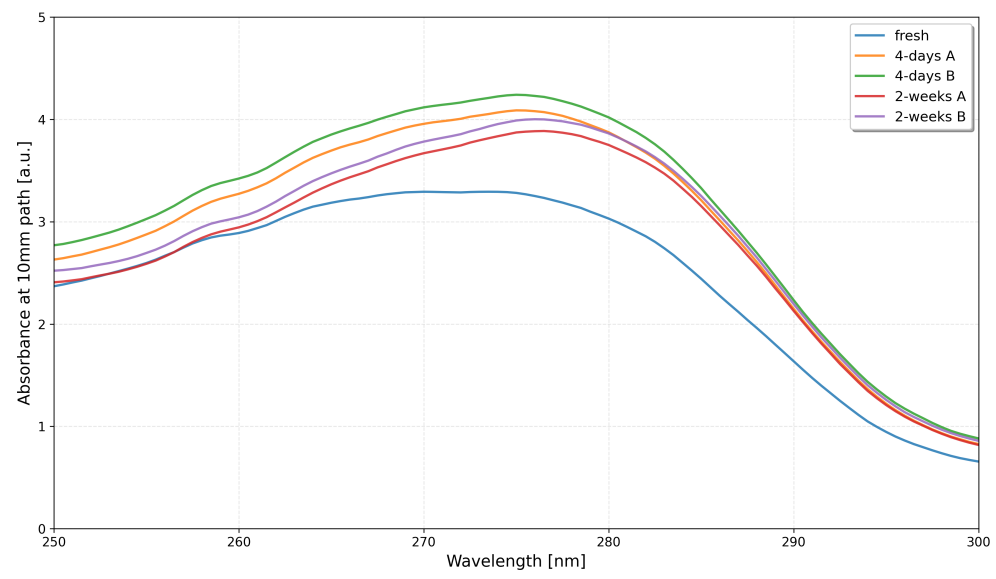

Fig. S36: Zoom in on the nanodrop analysis, small changes at the 275 nm peak are visible.

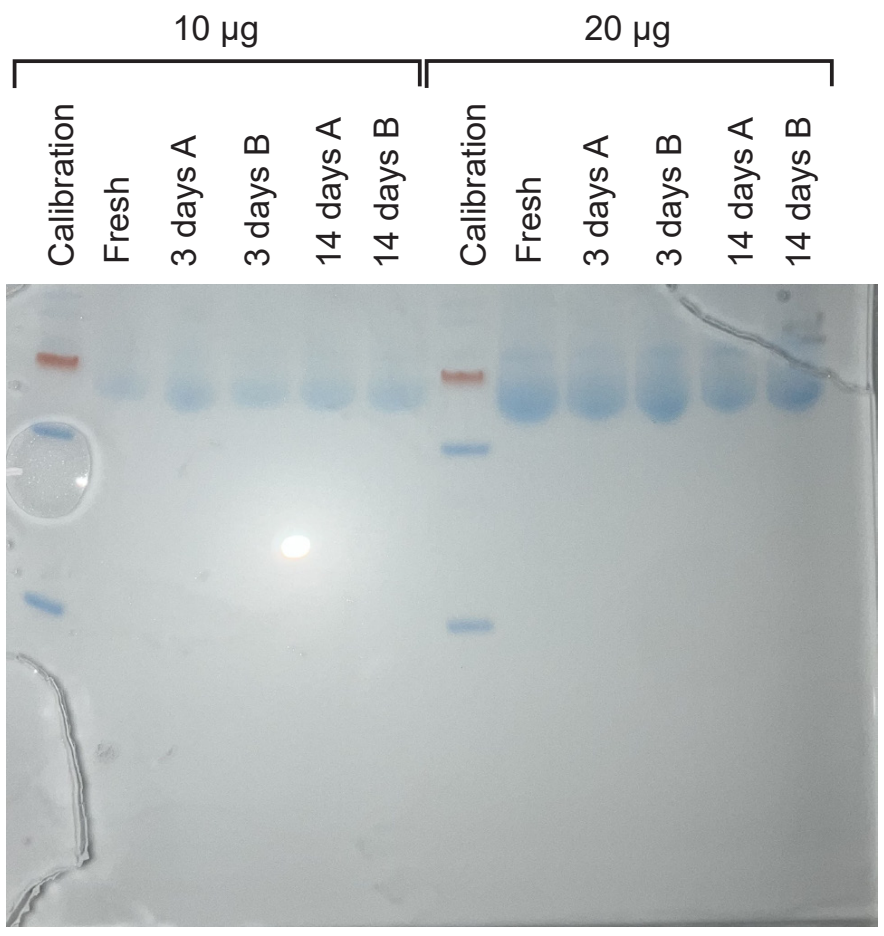

Fig. S37: Protein Electrophoresis (from left to right): calibration, fresh medium, 2 samples after 3 days, 2 samples after 14 days at 10 µg of protein and the same samples at 20 µg. No apparent differences in protein content are visible.

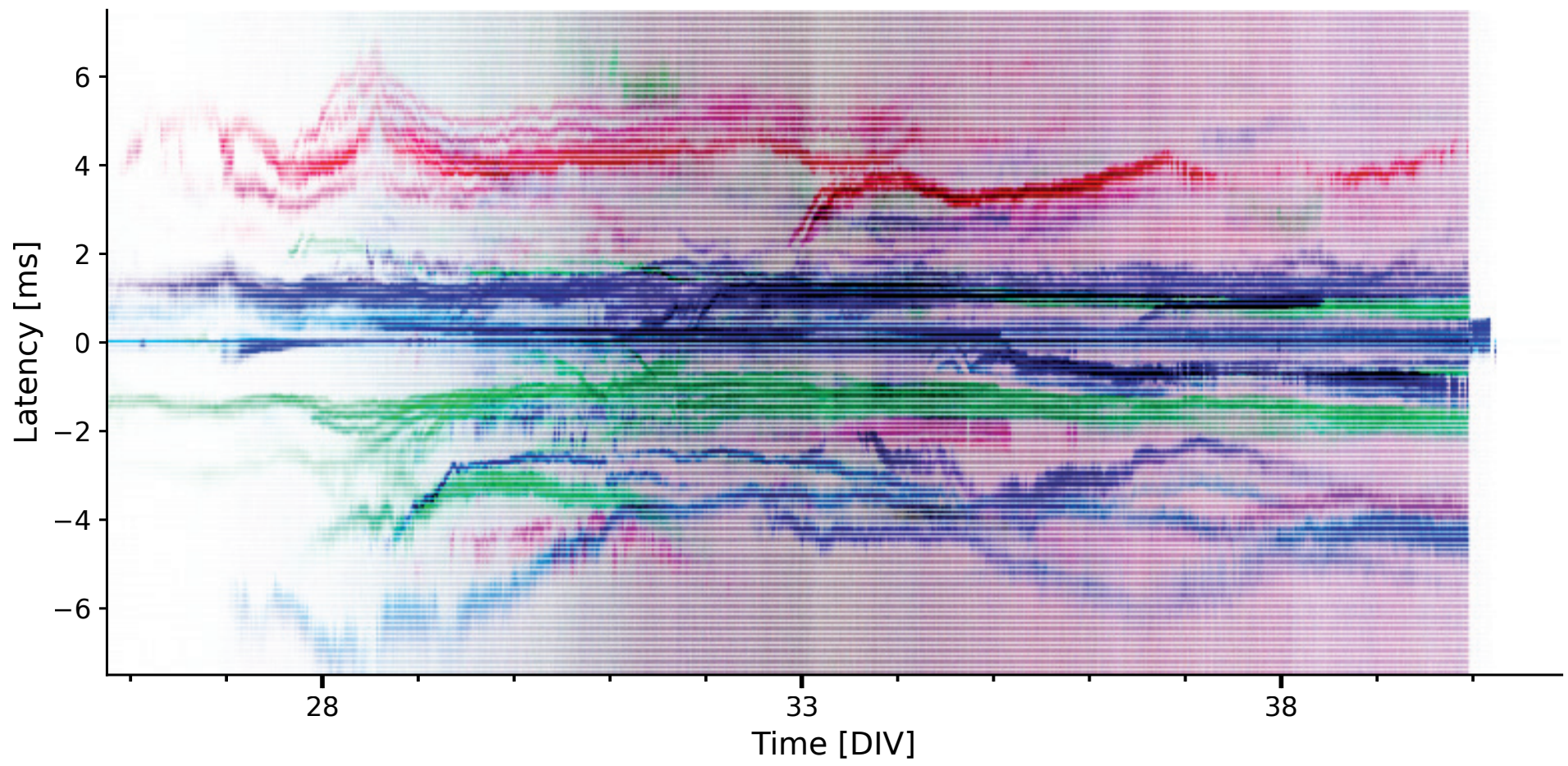

Fig. S38: Additional MEA (chip C, network 5) recorded for 14 days without medium exchange.

E Pattern analysis

E.1 Derivation of the analysis parameters

The integration window, the encoding kernel and the analysis window of the sequence analysis were checked against measurements on the data of network 2 of chip A. *Integration window.* Consecutive spikes recorded in the same region were paired and characterised by their temporal gap and the distance between the two electrodes (Fig. S39). The gaps scale with electrode separation at an apparent propagation speed of $0.30\,\mathrm{m\,s^{-1}}$, and 97.7 to 99.8 % of them fall inside the 1.5 ms merge window across all separations. *Encoding kernel.* The timing jitter between repeats of a pattern was measured as the difference in timing of corresponding region events between each sequence and its nearest neighbour in the encoding (Fig. S40). Its robust spread is 0.31 ms, so that the kernel width of $\sigma = 0.75\,\mathrm{ms}$ is a conservative choice. *Analysis window.* For every alignment event, the region events of the network were histogrammed as a function of their latency to the alignment event (Fig. S41a). In addition, repeats of a pattern were paired as nearest neighbours using only the first 4 ms after their alignment event, and their similarity was followed at later latencies, out-of-sample, against time-matched random pairs (Fig. S41b). The trigger-locked excess and the difference in similarity both decay before $\pm 10\,\mathrm{ms}$, so that extending the window would add baseline rather than structure.

E.2 Cluster overview and within-cluster similarity

Patterns sampled from the patterns identified through clustering of the deduplicated sequence set are shown in Fig. S42. The mean spatiotemporal matrix of each cluster is shown in Fig. S43, and the cosine similarity between sequences of the same and of different clusters in Fig. S44.

E.3 Cluster validity

All validation and robustness analyses were carried out on the deduplicated sequence set used in Fig. 4 and are scored against the partition of that set; values for the sequence set without deduplication, obtained with the same procedure, are given for comparison. Deduplication retained 61 % of the sequences, and the resulting partition agrees with the one before deduplication at ARI 0.88 (AMI 0.90). The partition was scored with three internal validity indices, each computed on the same subsamples for the partition itself, for a k-means partition with the same number of clusters, and for the labels shuffled at random (Table S3). The partition is far above the chance floor on all three indices. It is below the k-means reference, which is expected, since silhouette and Davies–Bouldin are defined in terms of compact, convex, similarly sized clusters, which k-means produces by construction and a density-based method is designed not to require. The density-based index DBCV depends strongly on the random seed of a single fit; over three seeds it is 0.20 ± 0.04 after and 0.23 ± 0.02 before deduplication. Per cluster (Fig. S45), the silhouette is negative for 6 of the 46 clusters.

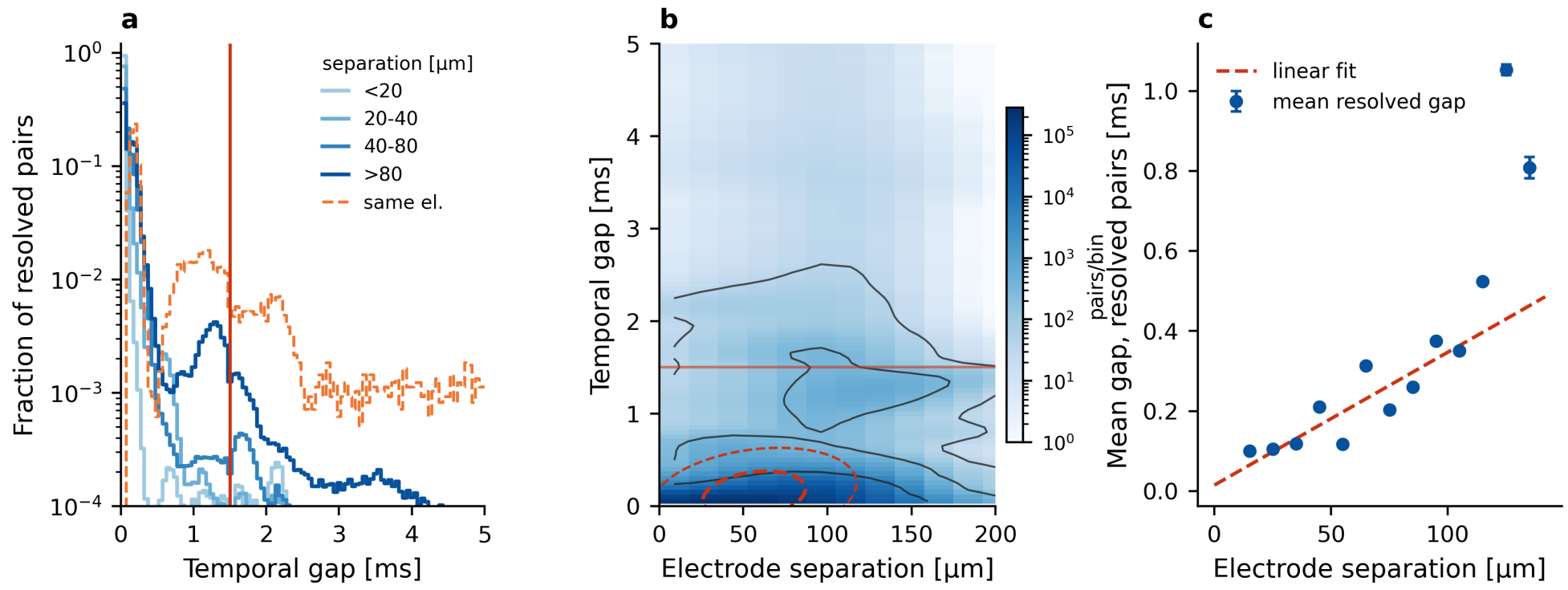

Fig. S39: **Within-region spike gaps resolved by electrode separation.** Consecutive spikes recorded in the same region of network 2 (chip A) were paired, and each pair characterised by its temporal gap and by the straight-line distance between the two electrodes. Electrode coordinates follow from the electrode index on the 220×120 array at 17.5 µm pitch; a straight microchannel is assumed, so the separation is a lower bound on the distance actually travelled. 8,140,030 pairs are shown. **a**, Distribution of temporal gaps, split by electrode separation (light to dark) and for pairs recorded on the same electrode (dashed). The distribution shifts to longer gaps as the electrodes move apart. The vertical line marks the 1.5 ms window used to merge spikes into a single region event; 99.8 % of pairs below 20 µm separation and 97.7 % of pairs beyond 80 µm fall below it, so the merge criterion holds across the whole array. **b**, Joint distribution of separation and gap (colour, smoothed counts per bin; black lines, density contours) with a two-dimensional Gaussian fitted to the same data (red, 1 and $2\,\sigma$). The two variables are positively correlated ($r = +0.25$ over the range shown; $r = +0.40$ against $\log_{10}$ gap over the full distribution). **c**, Mean gap of the resolved pairs against separation (mean ± s.e.m. per 10 µm bin) with a weighted linear fit (red). The slope corresponds to an apparent propagation speed of $0.30\,\mathrm{m\,s^{-1}}$ ($R^2 = 0.62$), and the intercept is +0.01 ms, i.e. zero delay at zero separation as expected. This is a lower bound on axonal conduction velocity, because temporally consecutive detections need not span the full electrode separation. Throughout, 56 % of pairs fall in the same 0.05 ms sample and have a gap of exactly zero; the panels describe the 44 % that are resolved in time, and the simultaneous fraction itself falls from 74 % to 47 % between 15 and 135 µm separation. Pairs on the same electrode (0.1 % of the total) are excluded from **b** and **c**. Histogram bins are one sampling interval (0.05 ms) wide.

E.4 Robustness of the clustering

Other algorithms

Five further clustering algorithms were applied to the same sequences in the same five-component space (Fig. S46, Table S4). k-means, a Gaussian mixture model and Ward agglomeration, which make different assumptions about cluster shape, reach an ARI of 0.72 to 0.73 with the partition of the main manuscript. The two HDBSCAN implementations agree less well (0.56 to 0.57) because of their handling of outliers.

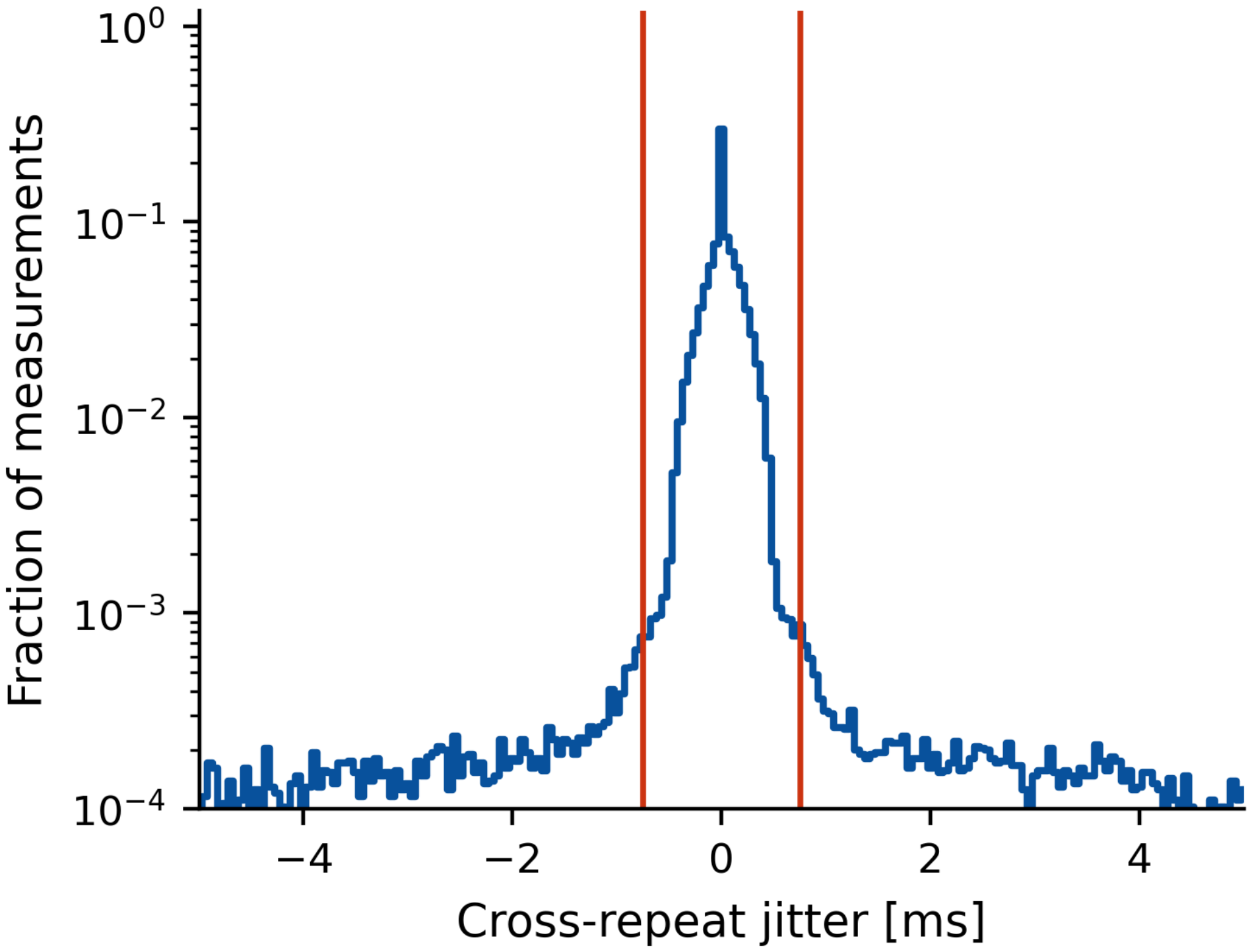

Fig. S40: **Timing jitter of region events between repeats of a pattern.** Network 2, chip A, deduplicated sequence set. Difference in the timing of corresponding region events between each sequence and its nearest neighbour in the ±10 ms window of the main manuscript (a region contributes only where it holds exactly one event in each sequence). Red lines mark the ±0.75 ms encoding kernel; the robust spread (IQR/1.349) is 0.31 ms.

Table S3: Internal validity of the partition, against a compact-cluster ceiling and a chance floor, for the deduplicated sequence set used in Fig. 4 and, for comparison, for the sequence set without deduplication. Mean over five independent draws of 50,000 sequences. Higher is better for silhouette and Calinski–Harabasz, lower for Davies–Bouldin.

	Silhouette		Davies–Bouldin		Calinski–Harabasz	
Partition	dedup.	w/o dedup.	dedup.	w/o dedup.	dedup.	w/o dedup.
HDBSCAN + nearest centroid	0.484	0.507	1.211	1.147	24,533	27,176
k-means reference	0.694	0.716	0.870	0.804	40,479	39,534
Shuffled labels (chance floor)	−0.039	−0.042	119.5	113.3	1	1

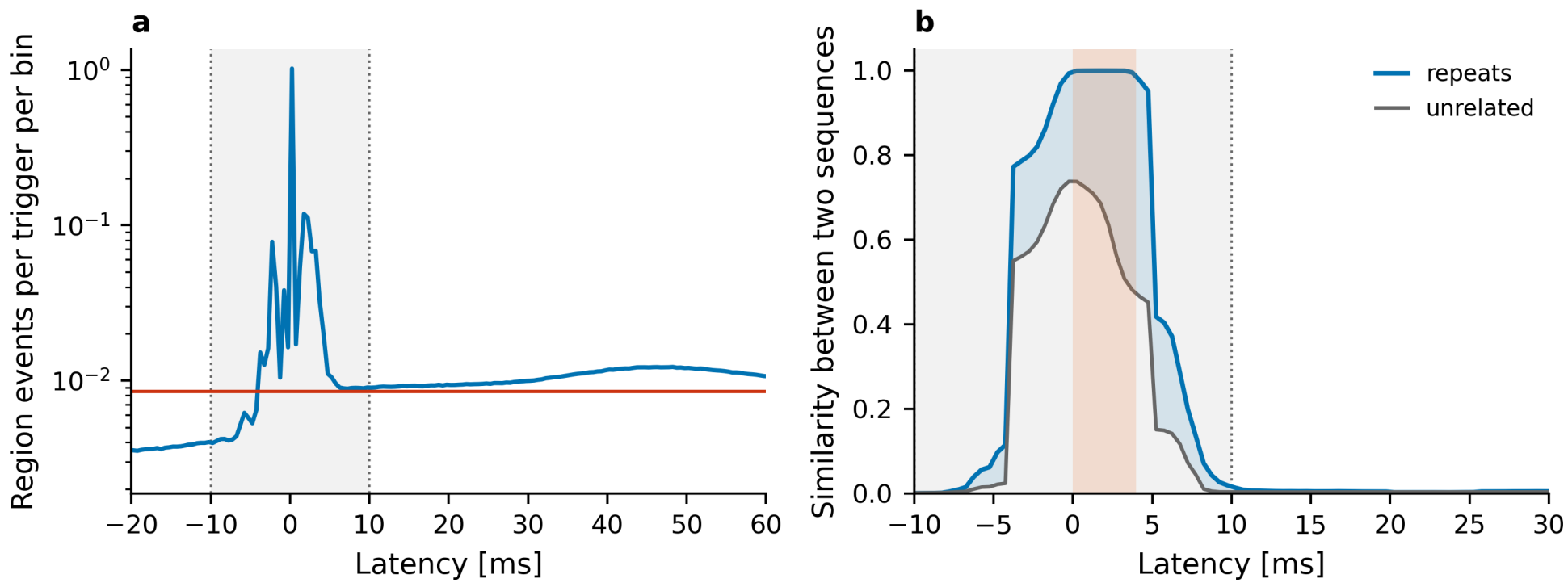

Fig. S41: **Choice of the ± 10 ms analysis window.** Network 2, chip A, deduplicated sequence set. **a**, Rate of region events per trigger and per latency bin, as a function of the latency from the alignment event. The excess over the flat baseline (red line) is the activity locked to the alignment event; 95 % of it lies within the shaded ± 10 ms window, and it has decayed to baseline before the window edge, so that activity beyond 10 ms is uncorrelated with the trigger. As a sanity check, the excess at negative latencies falls off within a few milliseconds, as expected for sparse firing in which an alignment event is rarely preceded by other activity. **b**, Similarity between two sequences as a function of latency. Repeats were paired as nearest neighbours using only the first 4 ms after their alignment event (partners at least 1 s apart in the recording), so that the similarity at all later latencies is measured out-of-sample. Similarity is the cosine similarity of the four-region activity in a 3 ms sliding window, for the paired repeats (blue) and for time-matched random pairs from the same hour (grey). The difference between the two decays to 10 % of its initial value by +8.8 ms, and less than 2 % of it remains at the window edge. Extending the window therefore adds baseline rather than structure.

Outliers

A fresh HDBSCAN run leaves about 20 % of the sequences unassigned, whereas the pipeline assigns every sequence to its nearest cluster centroid. With HDBSCAN's density-aware assignment, 20.1 % of the sequences would be outliers, but 98.0 % of the remainder receive the same cluster. Assigning all sequences keeps the pattern occurrence rates over time complete and avoids a density-dependent bias against rarer patterns.

Parameter stability

Varying `min_cluster_size` between half and twice the value of the main manuscript changes the number of clusters (76 at 1,000, 31 at 4,000) but not their quality: the silhouette of the assigned sequences stays between 0.83 and 0.88, and the ARI with the partition of the main manuscript between 0.56 and 0.59 (Table S5). These ARI

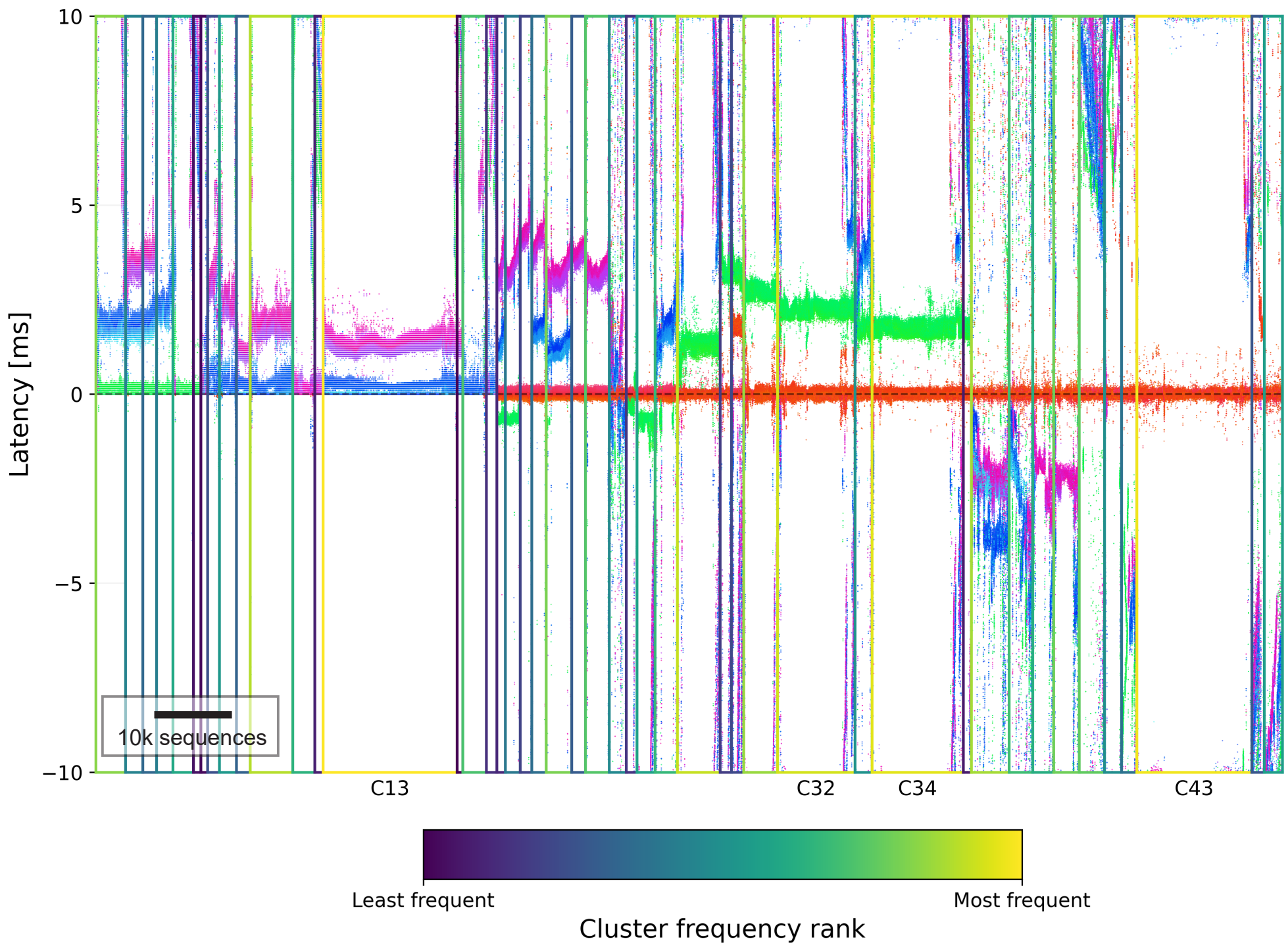

Fig. S42: **All clusters of the deduplicated sequence set.** Raster plot of 200,000 randomly sampled spatiotemporal spike sequences, sorted by the first principal component within each of the 46 clusters (labelled with C). Cluster rectangles are coloured by frequency rank (viridis: bright = most frequent). Scale bar: 10,000 sequences.

values are limited by the sequences HDBSCAN leaves unassigned; re-fitting the complete pipeline, including the nearest-centroid assignment, on independent subsamples reproduces the partition at ARI 0.98 (Fig. S48).

Other choices

Varying the number of principal components between 2 and 20 yields an ARI of 0.57 to 0.60 (Fig. S47). Over 12 bootstrap resamples, each re-clustered from scratch, the median cluster has a Jaccard similarity of 0.70 with its counterpart; 18 of 46 clusters exceed 0.75 and 11 fall below 0.5.

E.5 Functional connectivity over the recording

Delayed transfer entropy between the four regions was computed as for Fig. 2 on 10 min segments taken every 6 h over the whole long-term recording for network 2 of chip A and network 5 of chip C. Each network was compared with the network 6 h earlier and with the networks at all other time points (Fig. S49). This readout is independent of the clustering.

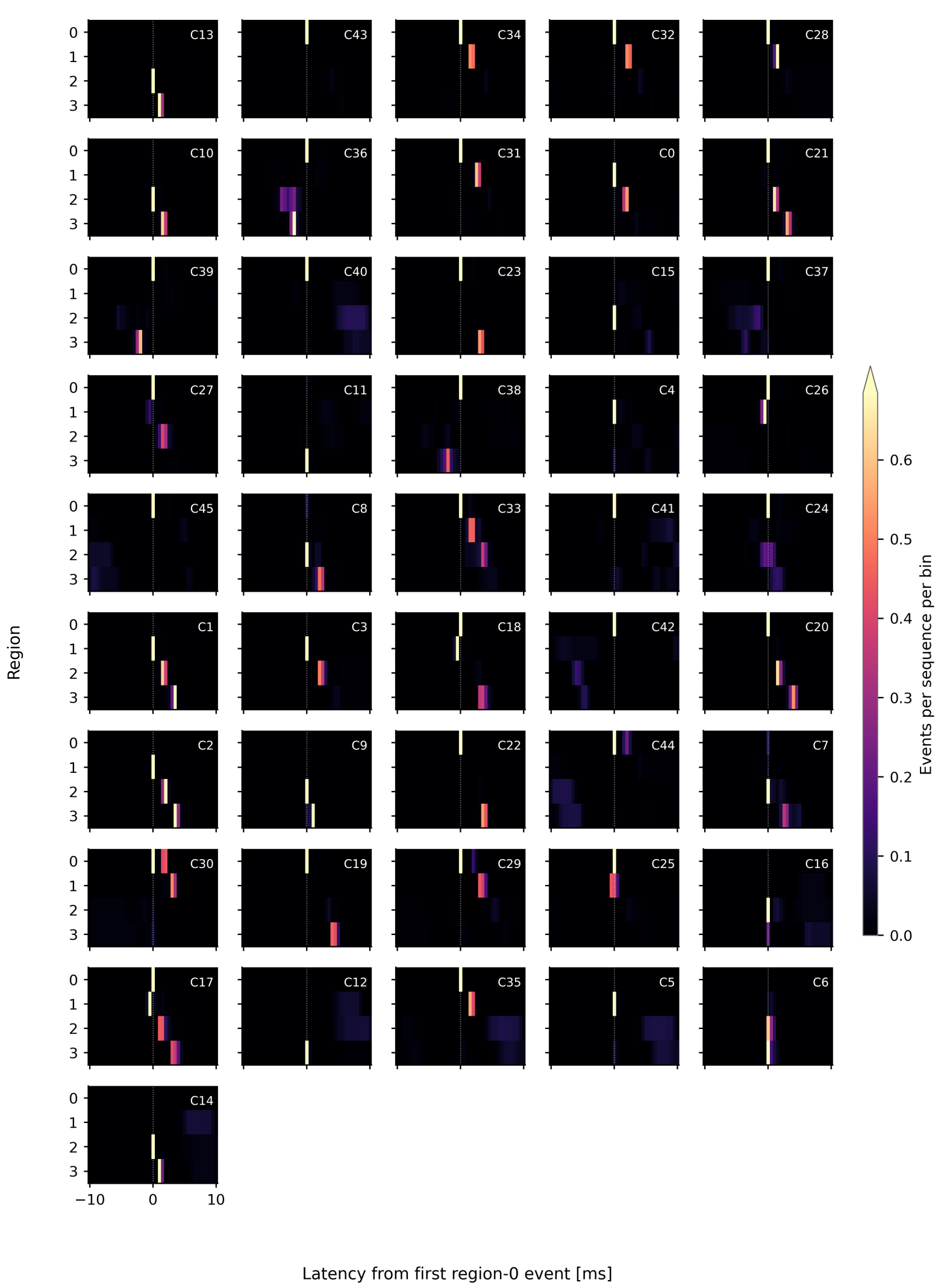

Fig. S43: **Mean spatiotemporal matrix of each cluster.** Network 2, chip A. Deduplicated sequence set. Mean spatiotemporal matrix of all 46 clusters, over all 15,130,738 sequences, one panel per cluster ordered by size and labelled with its cluster index.

E.6 Pattern analysis of further cultures

The full analysis, including deduplication, was repeated on two further cultures. Network 9 of chip D is the culture moved between conditions in Supplementary Note D.1, recorded from 49 DIV, which carries a microstructure of 12 networks with two seeding wells connected by ten parallel microchannels. Figs. S50 and S51 show its spike-time-triggered raster plots over two latency ranges, Fig. S52 the pattern repertoire over

Fig. S43: (continued) Each matrix is the four angular regions against latency from the alignment event (the first region-0 event, dotted line), in 0.5 ms bins centred on 0 ms. These are the region events as detected: the 0.75 ms Gaussian kernel that the clustering applies is *not* used here, so the panels show how sharply the events actually sit rather than the width of the kernel. Colour is the mean number of events a member of the cluster contributes to that region and bin, where 1.0 means every member fires there, on one scale for all panels, capped at 0.67. The bar at 0 ms is the alignment event itself, 1.0 in every cluster by construction; it is excluded when the cap is chosen and therefore saturates. Panels are in the same order and the same positions as the per-cluster separation figure, so the two can be read against each other panel for panel.

Table S4: Alternative clustering algorithms, run on the same sequences in the same five-component space, for the deduplicated sequence set used in Fig. 4 and, for comparison, for the sequence set without deduplication. ARI is measured against the partition of the respective set.

	Clusters		ARI	
Algorithm	dedup.	w/o dedup.	dedup.	w/o dedup.
k-means	46	45	0.73	0.75
Gaussian mixture	46	45	0.72	0.72
Ward agglomerative	46	45	0.73	0.72
HDBSCAN (hdbscan package)	45	44	0.57	0.62
HDBSCAN (scikit-learn)	44	44	0.56	0.62

Table S5: HDBSCAN re-run at half, one and two times the `min_cluster_size` of the main manuscript on the deduplicated sequence set, on a common pool of 200,000 sequences (the value is rescaled to the pool). Silhouette is computed over the assigned sequences only, since HDBSCAN's outlier label is not a cluster. ARI is measured against the partition of Fig. 4 on the same pool.

`min_cluster_size`	Clusters	Outliers	Silhouette	ARI
1,000	76	18.3%	0.879	0.59
2,000 (main manuscript)	45	20.6%	0.854	0.56
4,000	31	20.4%	0.828	0.56

time, and Fig. S53 the t-SNE embedding of the sequences. Network 5 of chip C is the additional network of Supplementary Note D.6, recorded for 14 days; its pattern analysis after deduplication is shown in Figs. S54 and S55. Over its shorter recording, this network shows similar trajectories to chip A.

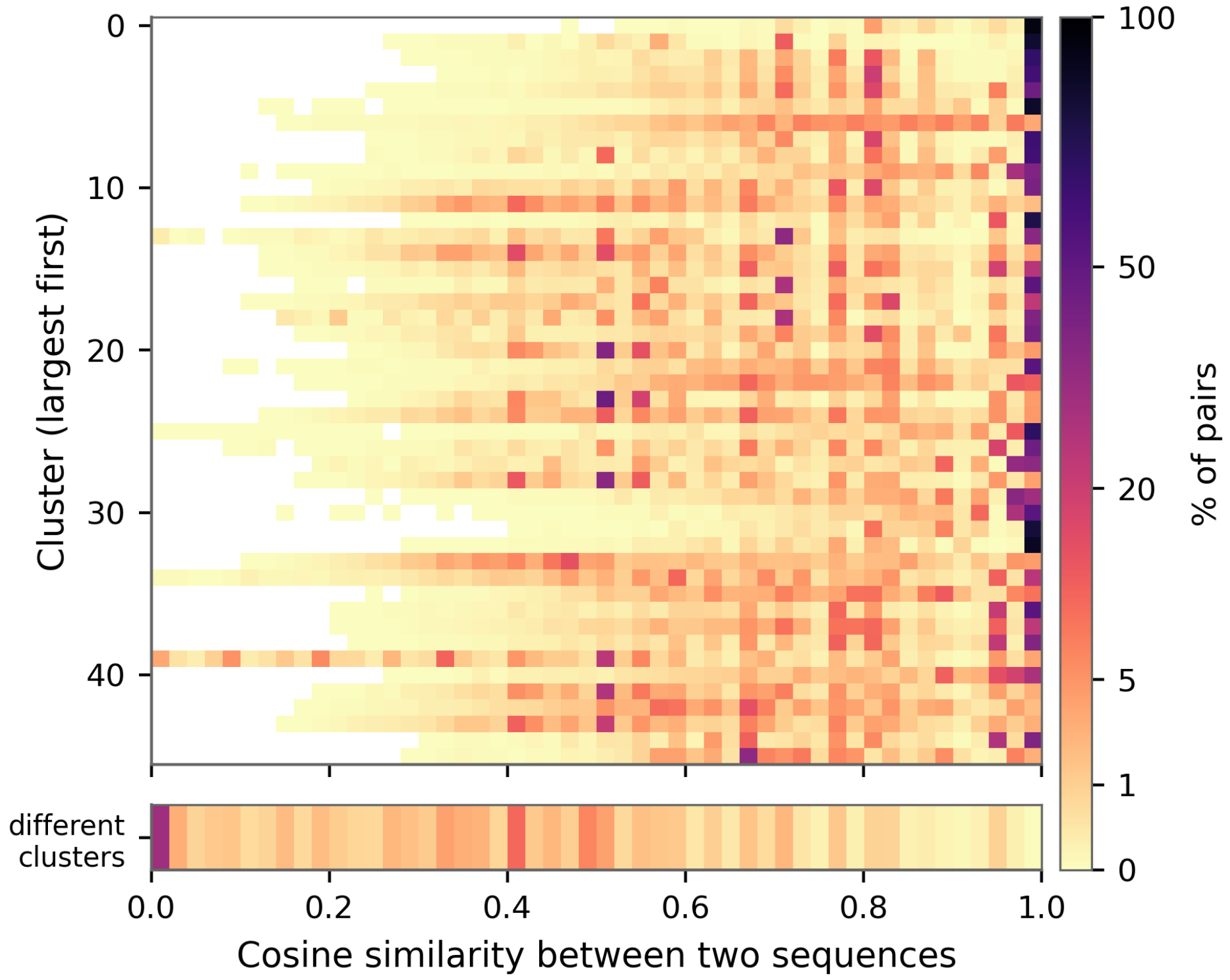

Fig. S44: **Cosine similarity of sequences within and between clusters.** Network 2, chip A. Deduplicated sequence set. For each of the 46 clusters, the distribution of the cosine similarity between two of its members in the 160-dimensional encoding the clustering used (up to 2,500 members sampled per cluster; every row sums to 100 %, rows ordered by cluster size). The bottom row is the null: pairs drawn from *different* clusters Taking the null's 95th percentile as the bar and weighting by cluster size, 77.5 % of same-cluster pairs are more similar than that, and 78 % in the median cluster; the size-weighted median similarity within a cluster is 0.89. The colour scale is square-root; the encoding is discrete, so a large share of each cluster's pairs are identical and fall in the single rightmost bin.

References

Duru J, Maurer B, Ruff T, et al (2024) A modular and flexible open source cell incubator system for mobile and stationary use. HardwareX 20:e00571. https://doi.org/10.1016/j.ohx.2024.e00571, URL http://dx.doi.org/10.1016/j.ohx.2024.e00571

Maurer B, Fassbind S, Ruff T, et al (2026) Inkube: an all-in-one solution for neuron culturing, electrophysiology, and fluidic exchange. Lab on a Chip https://doi.org/10.1039/d5lc00971e, URL http://dx.doi.org/10.1039/D5LC00971E

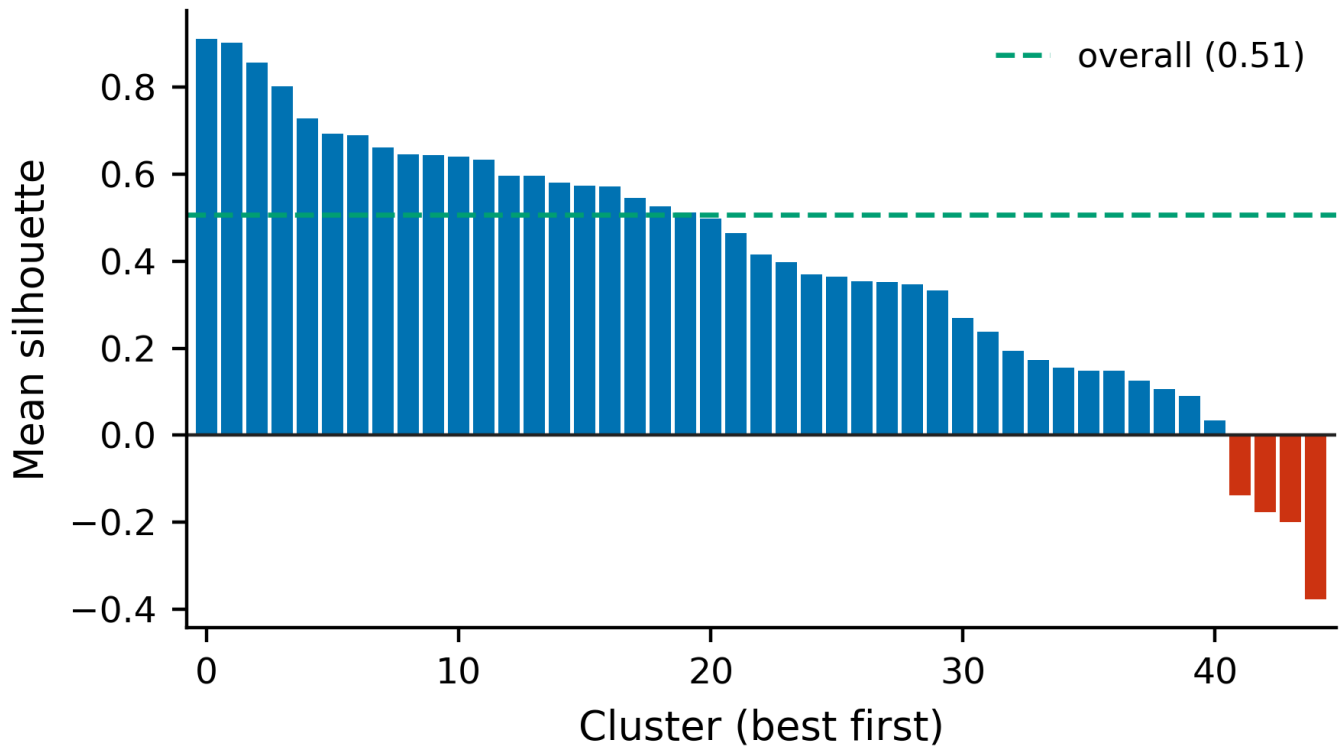

Fig. S45: **Silhouette of each cluster.** Network 2, chip A, deduplicated sequence set. Mean silhouette of each of the 46 clusters, sorted, on a draw of 50,000 sequences in the five-component space the clustering used. Silhouette runs from −1 (in the wrong cluster) through 0 (on the boundary) to +1 (perfectly placed). The dashed line is the value for the partition as a whole (0.48). Six clusters are negative (red).

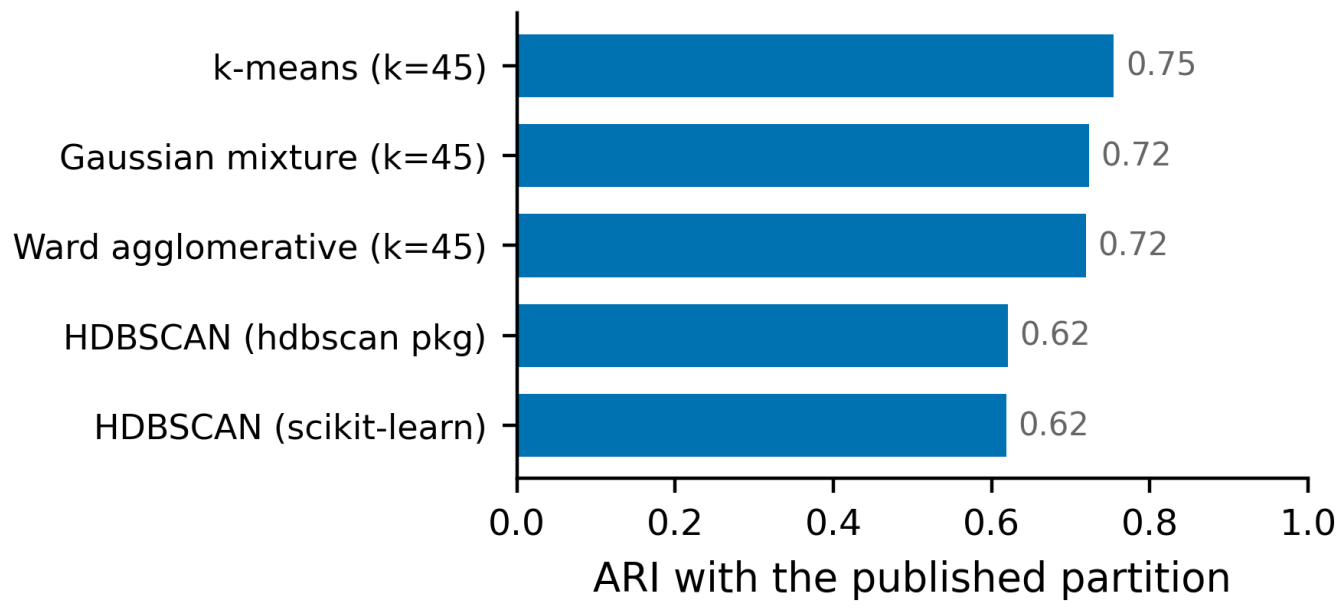

Fig. S46: **Agreement of alternative clustering algorithms with the partition.** Network 2, chip A, deduplicated sequence set. Adjusted Rand index between the partition used in Fig. 4 and each of five alternative algorithms run on the same sequences in the same five-component space. ARI is 1 for identical partitions and 0 for the agreement expected by chance.

Wagenaar DA, Pine J, Potter SM (2006) An extremely rich repertoire of bursting patterns during the development of cortical cultures. BMC Neuroscience 7(1). https://doi.org/10.1186/1471-2202-7-11, URL http://dx.doi.org/10.1186/1471-2202-7-11

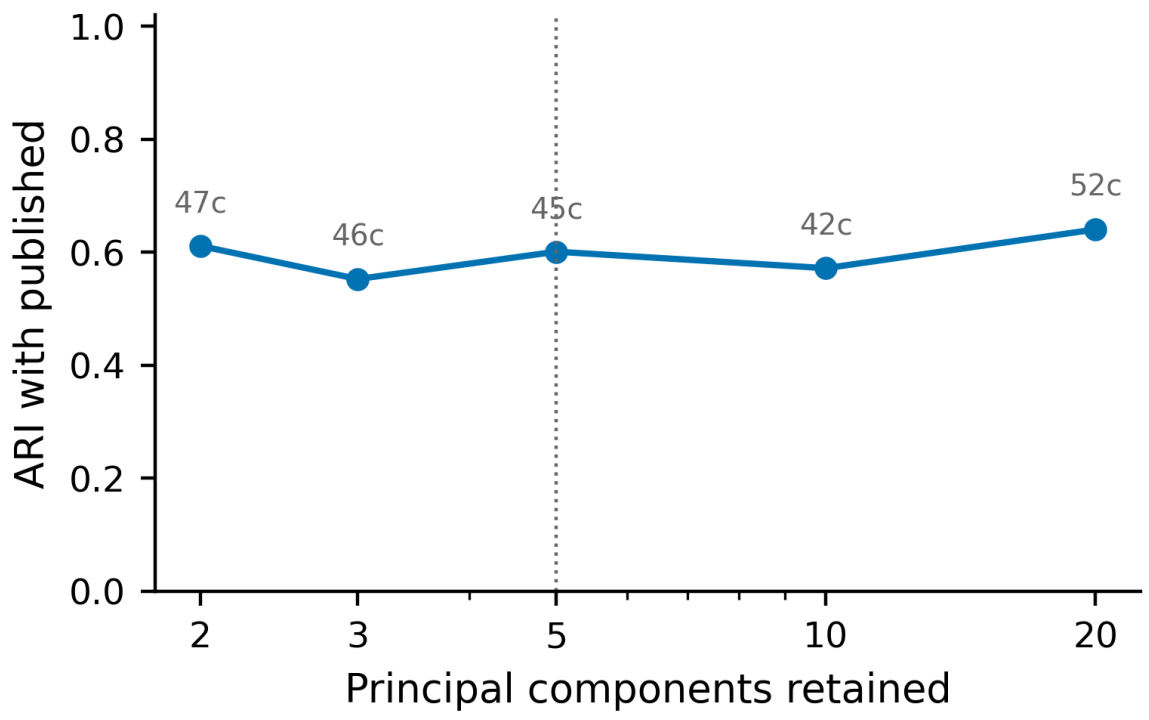

Fig. S47: **Dependence of the clustering on the number of principal components.** Network 2, chip A, deduplicated sequence set. HDBSCAN re-run on 2, 3, 5, 10 and 20 principal components of the same encoding, scored by ARI against the five-component partition; labels give the number of clusters found and the dotted line marks the choice used in Fig. 4. Agreement stays between 0.57 and 0.60 and the cluster count between 40 and 47 across a tenfold range, over which the variance explained rises from 43 % to 90 %.

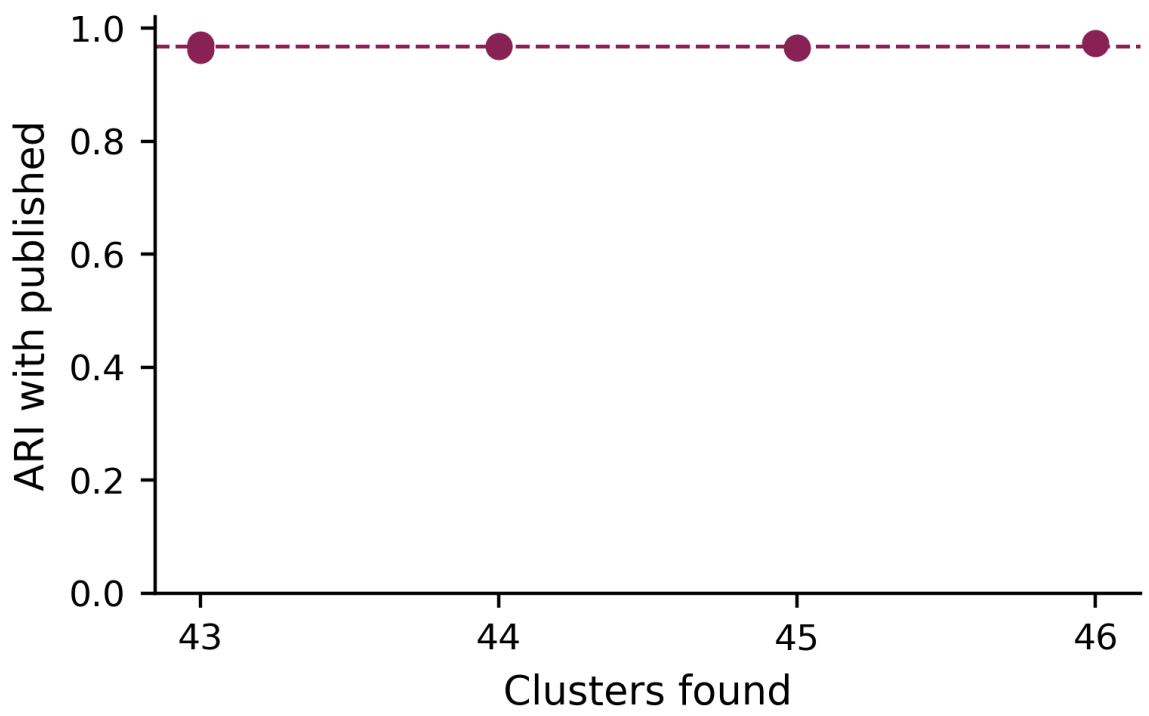

Fig. S48: **Re-fitting of the clustering on independent subsamples.** Network 2, chip A, deduplicated sequence set. The pipeline of Fig. 4 was run again from scratch on 5 independent random subsamples of the same size, each scored by ARI against the partition of Fig. 4 (dashed line: mean 0.98). The cluster count varies between 44 and 48 while agreement stays at 0.98 ± 0.01, indicating that the variation concerns the splitting of a few marginal clusters. Splitting the recording into interleaved hours between DIV 10 and 24 and fitting each half separately gives ARI 0.55 across halves in both directions. Three of the 5 runs found the same number of clusters and plot almost on top of each other.

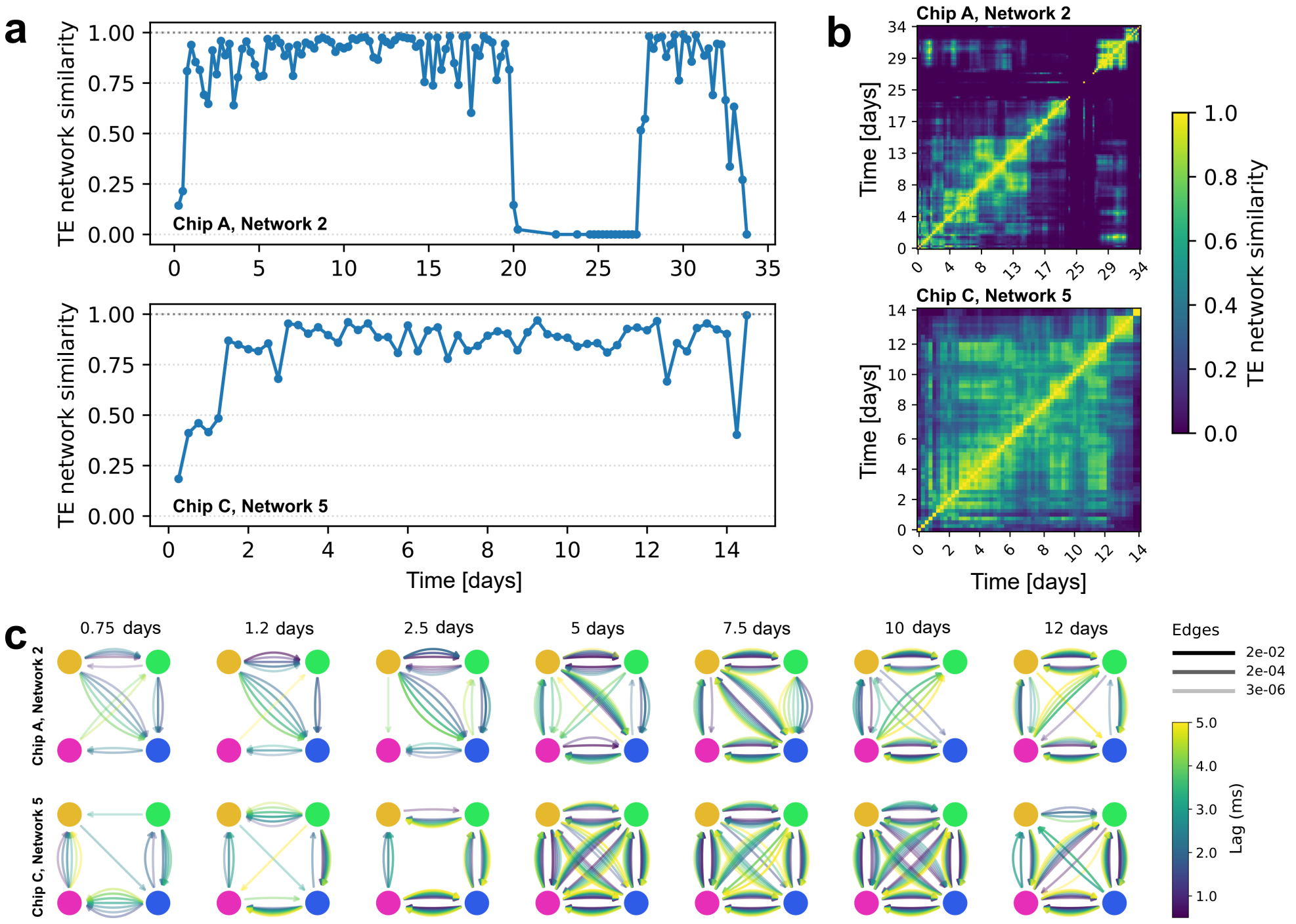

Fig. S49: **Functional connectivity over the recording.** Delayed transfer entropy between the four regions, computed as for Fig. 2 (Methods) on 10 min segments taken every 6 h, for chip A / network 2 (top) and chip C / network 5 (bottom). **a**, Similarity of each transfer-entropy network to the network 6 h earlier. On the timescale of a few hours, the networks are stable. **b**, All-to-all cosine similarity matrices of the same networks. Similarity remains high throughout; most rows appear multimodal rather than centred on the diagonal, indicating recurring periods of higher similarity rather than a uniform drift. **c**, Network graphs at selected time points; node colour is region, edge width is transfer entropy and edge colour is interaction lag.

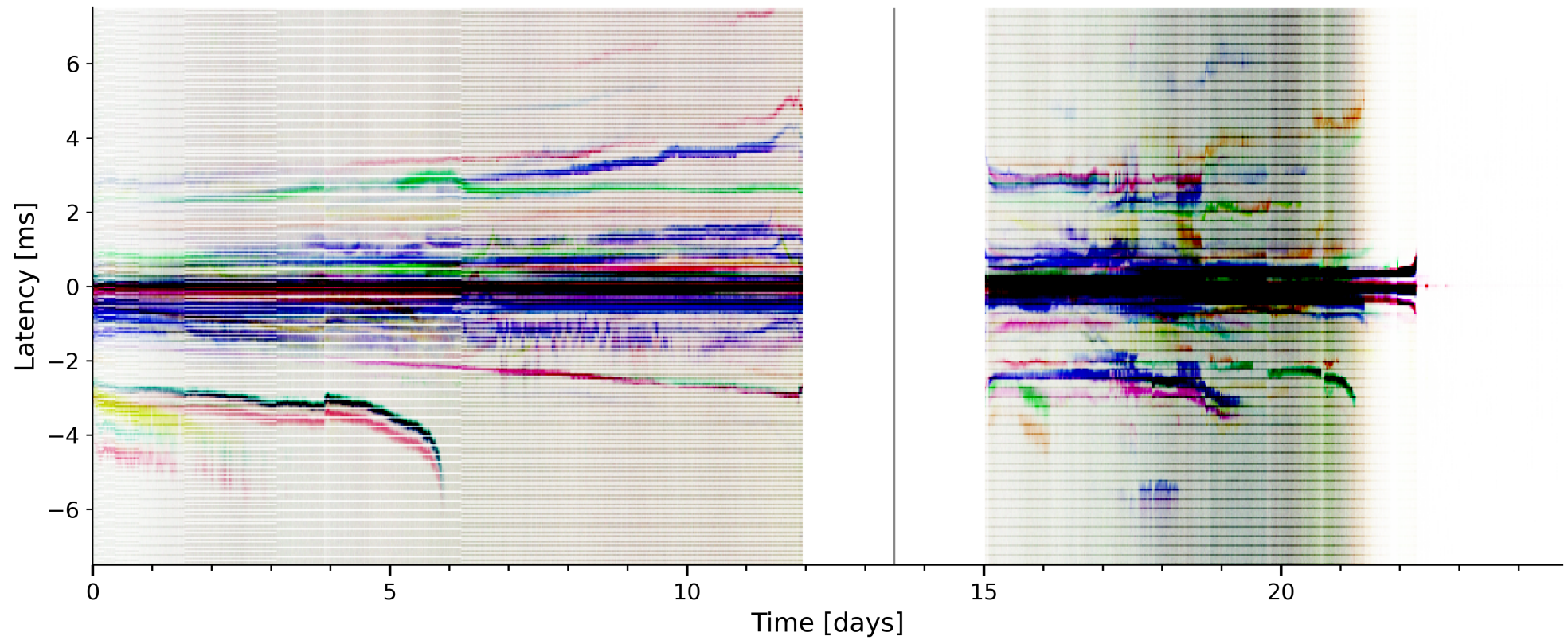

Fig. S50: **Spike-time-triggered raster plot of a culture on a different microstructure.** Chip D, network 9 (the culture of Fig. S27); recording started at 49 DIV (day 0). Spike-time-triggered raster plot as in Fig. 3c–e: latency of spikes on the network electrodes relative to the alignment spike against recording time, over ±7.5 ms, colour-coded by electrode position, in 1,800 s bins with pixel saturation at 3,600 spikes per bin (2 Hz). Several patterns are held simultaneously and persist over days, as in network 2 of chip A. Recording was paused between days 12 and 15 (61 to 64 DIV), when the culture was kept in a standard incubator; from day 15 onwards it was recorded under a commercial membrane lid in a standard incubator, where activity became unstable and ceased at around day 23 (72 DIV).

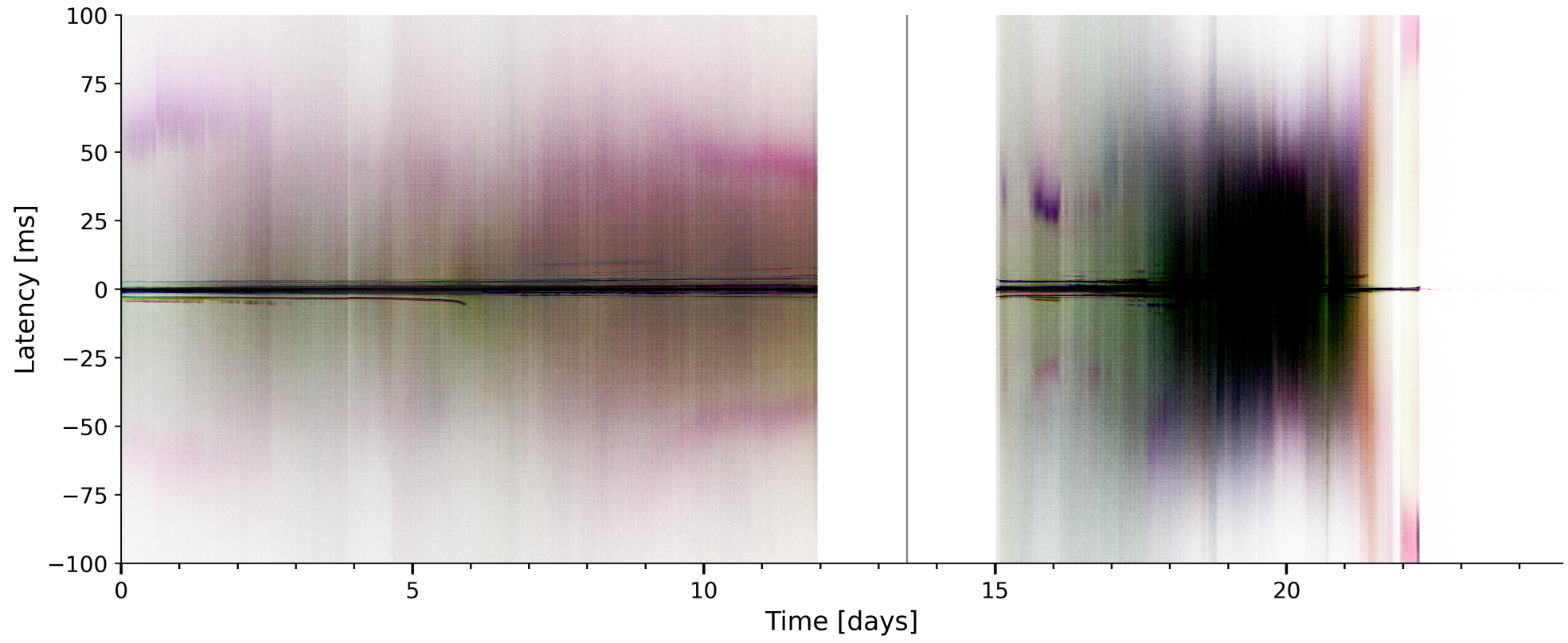

Fig. S51: **Spike-time-triggered raster plot of the same culture over a wider latency range.** Chip D, network 9, recording started at 49 DIV; as Fig. S50 but over ±100 ms, with pixel saturation at 776 spikes per bin. The sharp and stable structure is confined to the short-latency band used in the analysis; at longer latencies only broad, weak bands (around ±50 ms, compare Fig. 3c) and an unstructured background are visible.

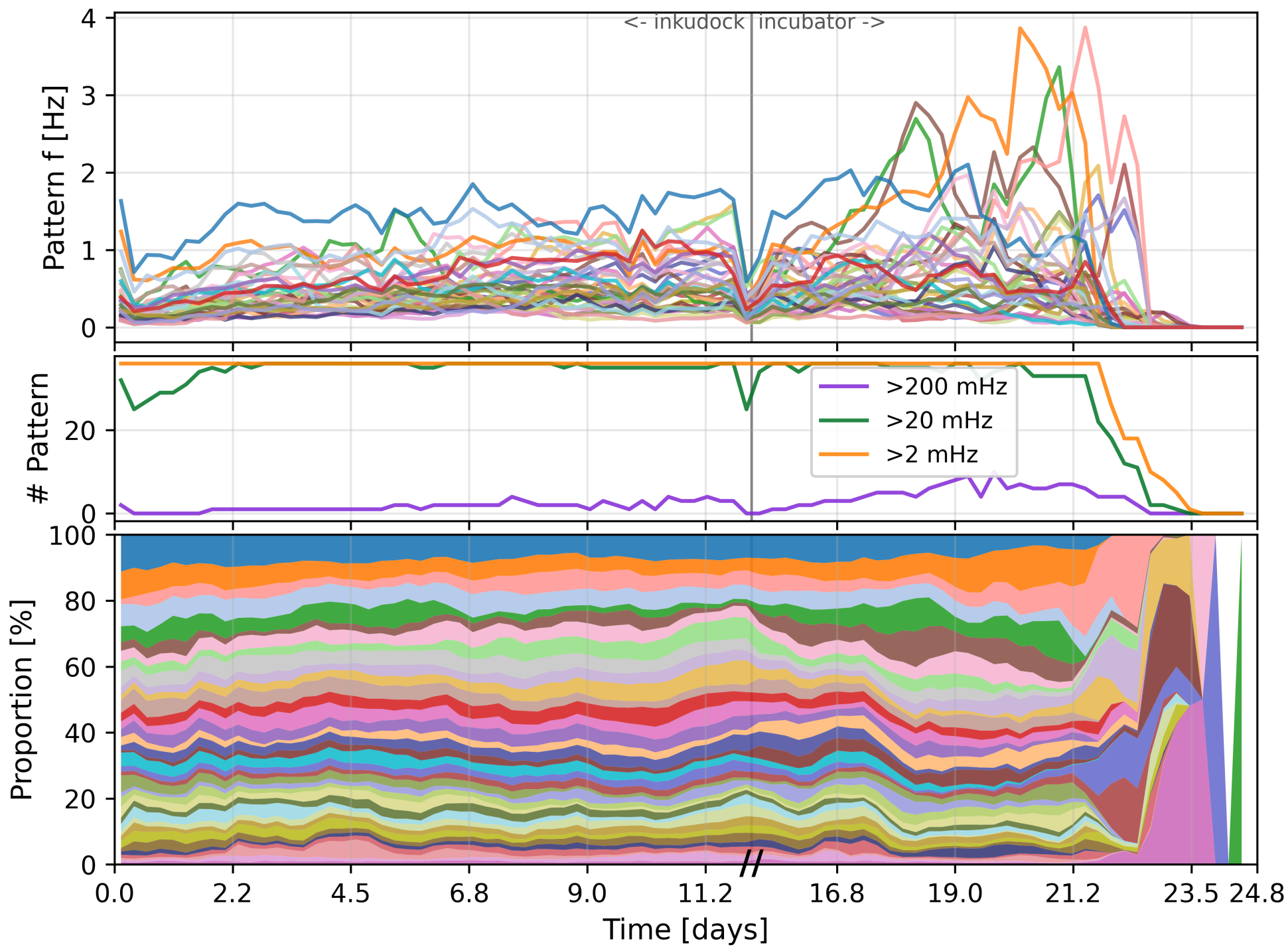

Fig. S52: **Pattern occurrence in the culture on a different microstructure.** Chip D, network 9, recording started at 49 DIV (day 0); deduplicated sequence set, analysed as in Fig. 4f–h. Top, occurrence rate of each cluster over the recording. Middle, number of patterns exceeding 2, 20 and 200 mHz. Bottom, the proportion of all sequences accounted for by each cluster. The vertical line marks the transfer from inkudock to a standard incubator (recording paused between days 12 and 15, i.e. 61 to 64 DIV, indicated by the axis break). After the transfer, the pattern rates first dip, then rise between days 17 and 21 (66 to 70 DIV), before activity collapses and ceases at around day 23 (72 DIV), as does the firing rate of the same culture (Fig. S27).

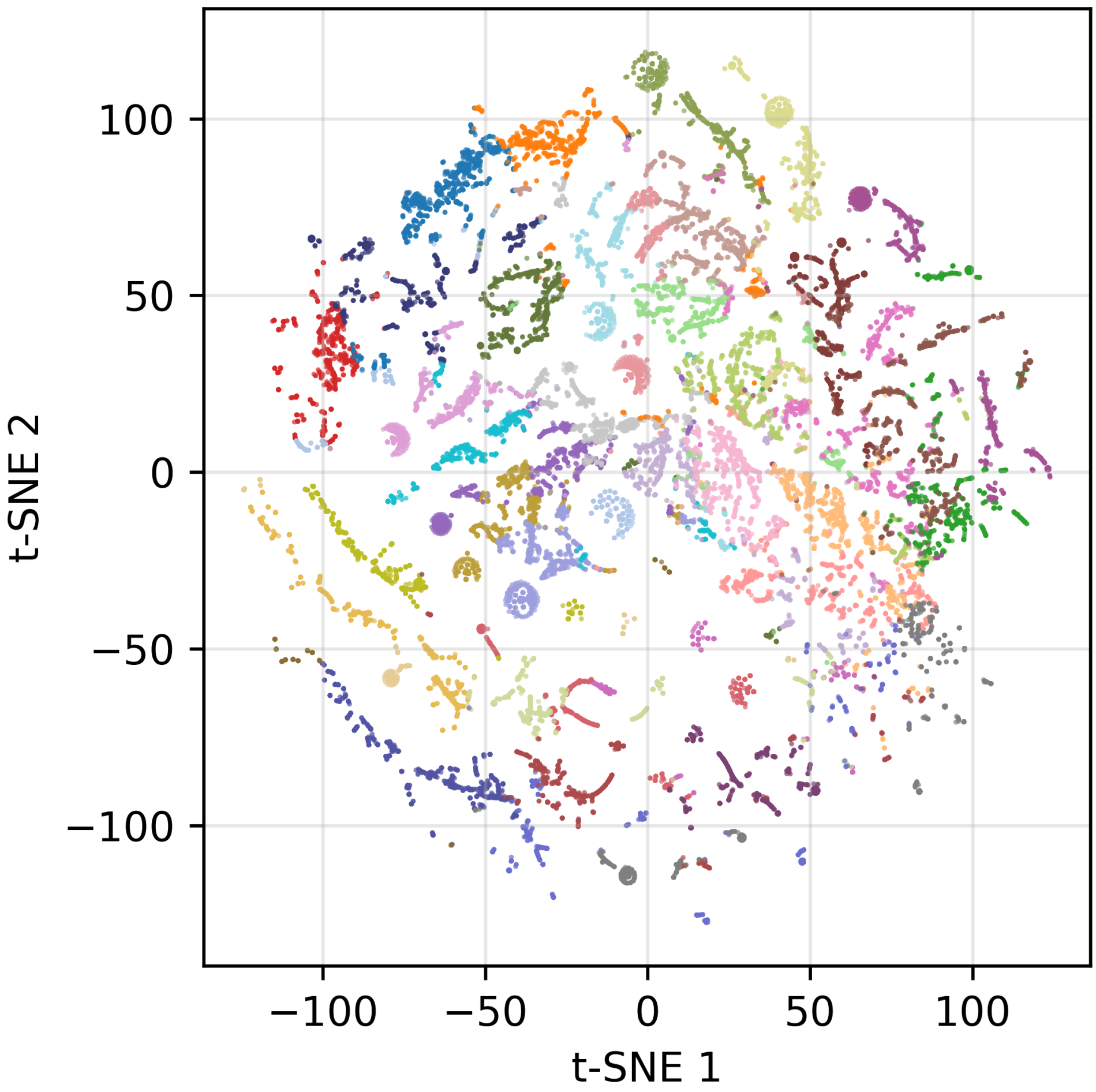

Fig. S53: **t-SNE embedding of the sequences of the culture on a different microstructure.** Chip D, network 9, recording started at 49 DIV; deduplicated sequence set. t-SNE embedding of the sequence encoding, coloured by HDBSCAN cluster. The clusters form discrete, well-separated groups, as for chip A (Fig. 4e).

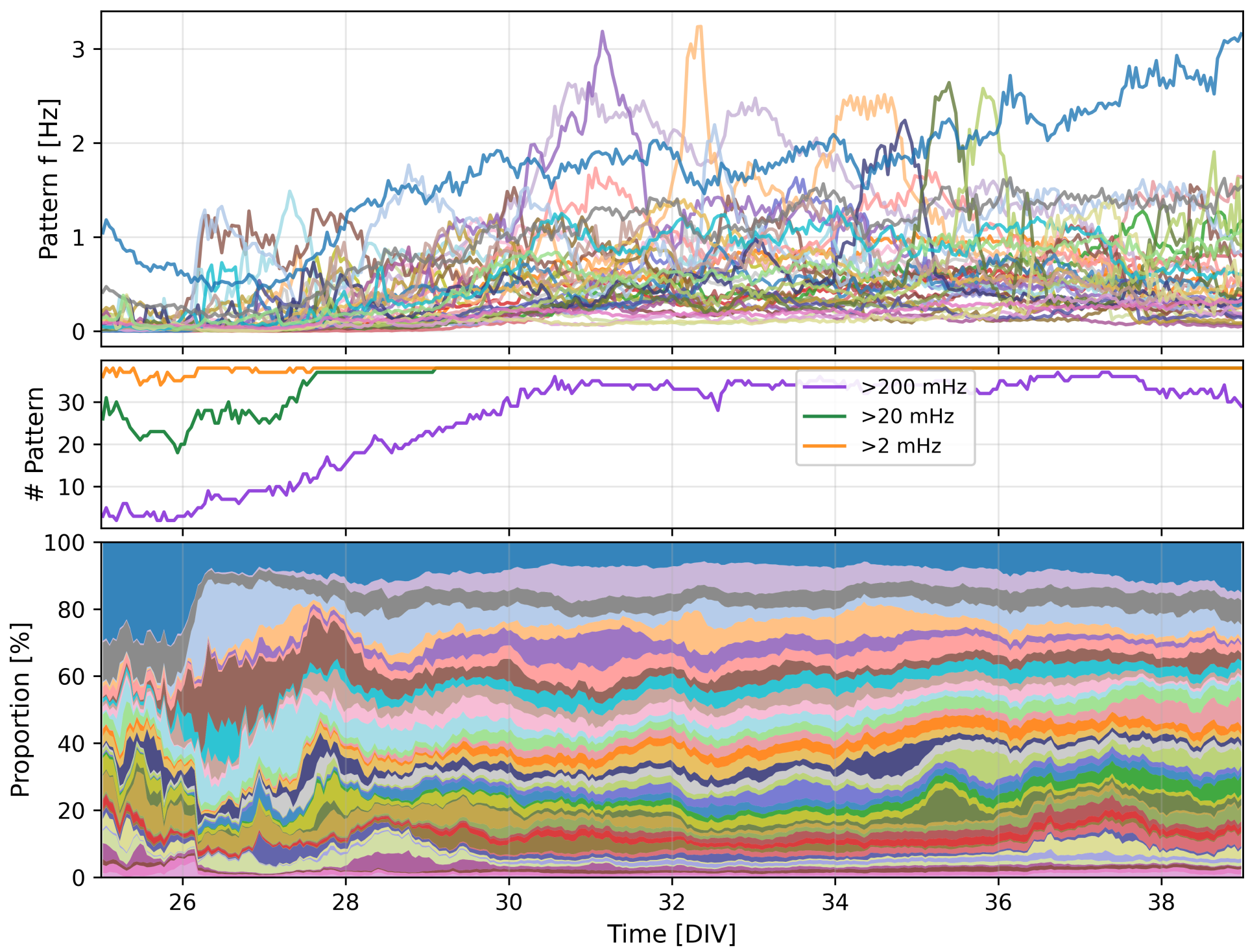

Fig. S54: **Pattern occurrence in the additional network.** Chip C, network 5 (Fig. S38), deduplicated sequence set, analysed as in Fig. 4f–h. Deduplication (Methods) retained 48 % of the sequences of this network; the partition agrees with the one obtained without deduplication at ARI 0.61.

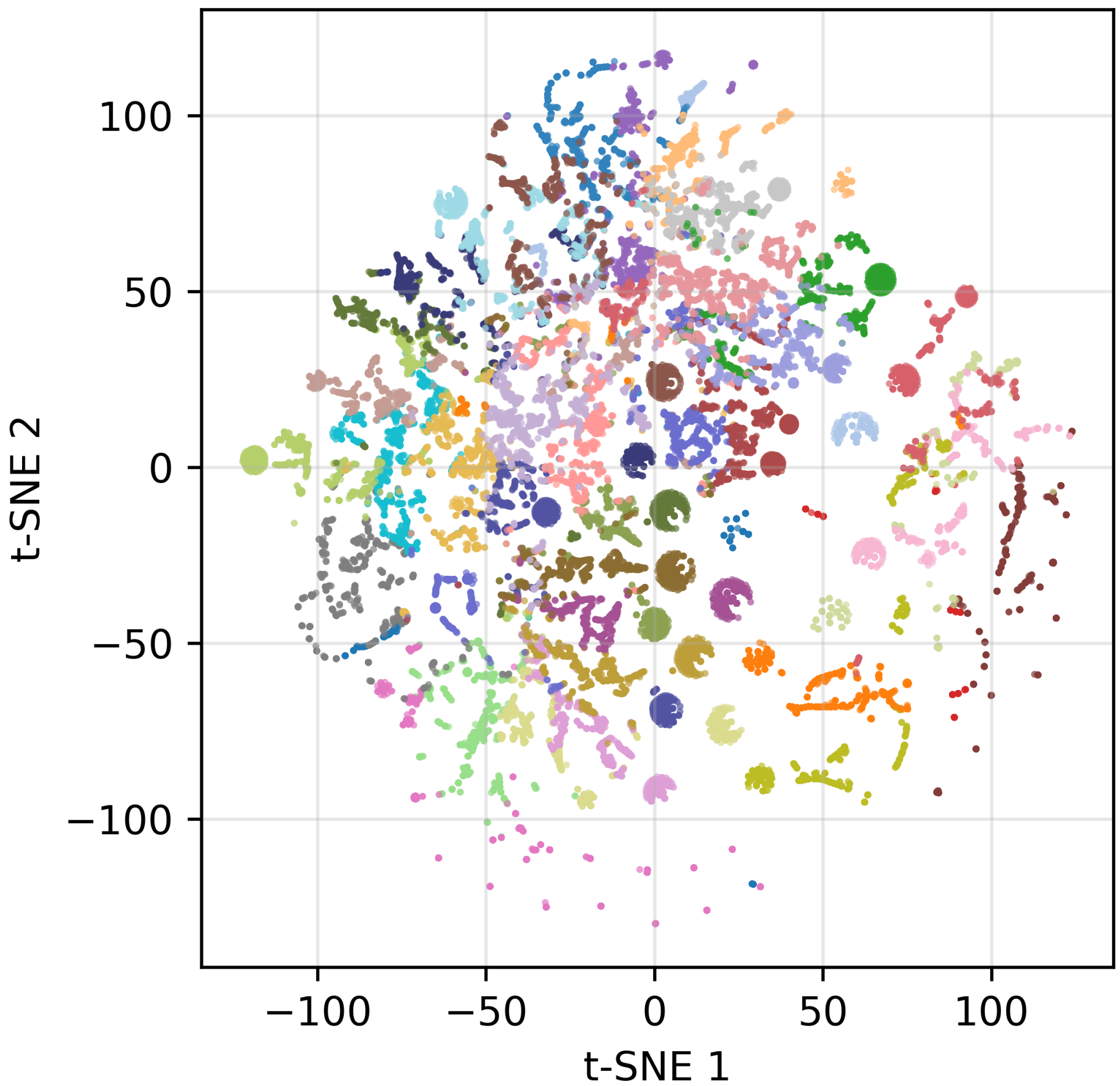

Fig. S55: **t-SNE embedding of the sequences of the additional network.** Chip C, network 5, deduplicated sequence set; t-SNE embedding of the sequence encoding, coloured by HDBSCAN cluster (38 clusters). Validation as in Figs. S45–S48: silhouette 0.13 (k-means reference 0.38), ARI of alternative algorithms with the partition 0.35 to 0.42, ARI between two interleaved halves of the recording 0.11 and 0.13, median bootstrap Jaccard 0.40.
